# A Kinetic Self‐Sorting Approach to Heterocircuit [3]Rotaxanes

**DOI:** 10.1002/anie.201606640

**Published:** 2016-09-07

**Authors:** Edward A. Neal, Stephen M. Goldup

**Affiliations:** ^1^ School of ChemistryUniversity of SouthamptonHighfieldSouthamptonSO17 1BJUK; ^2^ School of Biological and Chemical SciencesQueen Mary University of LondonUK

**Keywords:** active templates, cycloaddition, CuAAC, rotaxanes, self-sorting

## Abstract

In this proof‐of‐concept study, an active‐template coupling is used to demonstrate a novel kinetic self‐sorting process. This process iteratively increases the yield of the target heterocircuit [3]rotaxane product at the expense of other threaded species.

The synthesis of heterocircuit rotaxanes in which several different rings are threaded onto a single axle is complicated significantly by the potential for sequence isomerism: even with just two distinct macrocycles, a heterocircuit [3]rotaxane with a non‐centrosymmetric axle exists as two isomers, and the stereochemical complexity rises as the number of rings increases.^1^, ^2^ Although the controlled synthesis of heterocircuit [*n*]catenanes is relatively well‐developed,^3^ including Stoddart and co‐worker's landmark synthesis of olympiadane in 1994,^4^ there remain relatively few examples of the synthesis of hetero[*n*]rotaxanes with control over the relative order of the macrocyclic components.^5^

Conceptually, the simplest approach to the synthesis of heterocircuit [*n*]rotaxanes is to employ an axle with multiple orthogonal binding sites for the various rings that are then installed in a stepwise manner. However, this raises the synthetic complexity of the thread, and only limited, stereochemically trivial examples have been reported.^6^ Alternative stepwise approaches to heterocircuit [*n*]rotaxanes have been developed; Sauvage and co‐workers reported the coupling of kinetically inert pseudorotaxane complexes,^7^ and in 2010 Leigh and co‐workers used an iterative clipping of macrocycles around a single binding site to produce both stereoisomers of a [3]rotaxane in a stepwise manner.^8^

Self‐sorting, in which multiple components selectively assemble themselves into complex architectures,^9^ is a particularly successful and attractive approach for the synthesis of complex supramolecular systems^10^, ^11^, ^12^ and materials.^13^ In 2008, Schalley and co‐workers demonstrated a self‐sorting method for stereospecific [3]rotaxane synthesis by using the steric requirements of threading (rather than the affinity of the macrocycles for a given binding site) to determine the arrangement of rings on the axle, and this approach has since been extended to more complicated architectures.^14^, ^15^ More recently, Stoddart and co‐workers introduced the “cooperative capture” method,^16^ in which the synergistic binding of guests by cucurbiturils and cyclodextrins,^17^ or cucurbiturils and Ogoshi's pillarenes,^18^ produces pre‐organized heterocircuit pseudorotaxanes, which are then captured using Steinke's catalytic self‐threading reaction,^19^ in excellent yield with high stereospecificity.

Self‐sorting reactions typically operate under thermodynamic control, allowing the system to correct errors, although the trajectory to achieve equilibrium can be kinetically complex.12c,12d, 15d, 20 In keeping with this, previous reports of self‐sorting [*n*]rotaxane synthesis rely on passive templates to direct the assembly of a thermodynamically preferred pseudorotaxane complex before covalent bond formation kinetically traps the interlocked assembly. Leigh's active template (AT) approach to mechanical bond formation is unusual in that the mechanical bond can, in theory, be formed solely under kinetic control; the covalent bond forming reaction simply takes place faster through the cavity of the macrocycle.^21^ As a consequence, an unusual feature of AT‐derived products is that they often retain only weak attractive interactions between the covalent subcomponents and thus are typically unstable with respect to dethreading.

This suggests an opportunity to develop self‐sorting reactions that are governed only by the kinetic stability of the products. Herein we report the realization of this proposal: a stereoselective four‐component coupling in which kinetic self‐sorting amplifies the yield of a target heterocircuit [3]rotaxane.

Our proposed AT self‐sorting process (Figure 1) requires two macrocycles, **I** and **II**, with different internal diameter and two half‐threads, **III** and **IV**, of different steric bulk. Focusing on the fate of the larger of the two rings (**I**, blue), four threaded products are possible: [2]rotaxane **V**, homocircuit [3]rotaxane **VII**, and stereoisomeric heterocircuit [3]rotaxanes **VIII** and **IX**. If only one of the half‐threads is bulky enough to retain the larger macrocycle this mixture is simplified as macrocycle **I** can dethread in all cases except product **VIII**, in which its path is blocked by smaller macrocycle **II**, which is in turn held in place by the smaller stopper, an example of Schalley's cascade stoppering.14a Crucially, the escape of macrocycle **I** renders it available for further AT coupling, suggesting that this “ratcheted” self‐sorting process will amplify the yield of **VIII** above the natural selectivity of the reaction. Overall, [3]rotaxane **VIII** is the only stable interlocked product derived from **I**, alongside the products of dethreading (recovered **I** non‐interlocked thread **X** and [2]rotaxane **VI**) and the products of direct AT couplings of macrocycle **II**.

**Figure 1:**
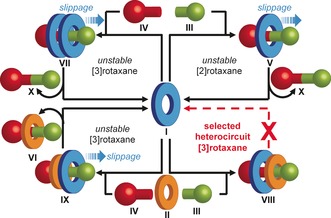
Proposed self‐sorting approach to [3]rotaxanes.

We previously observed homocircuit [3]rotaxane formation in high yield when certain bipyridine macrocycles^22^ were used in Leigh's^23^ AT Cu‐mediated alkyne–azide cycloaddition^24^ (AT‐CuAAC) reaction.^25^ Thus, to develop our proof‐of‐concept self‐sorting process, we set out to explore the mixed‐macrocycle AT‐CuAAC reaction in the presence of macrocycles **1** and **2** (Scheme 1), which have previously been shown to have significantly different propensities for [3]rotaxane formation.^25^

**Scheme 1:**
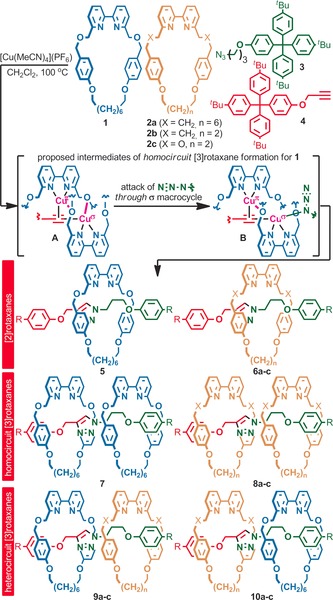
The AT‐CuAAC reaction with macrocycles **1** and **2**. Reagents and conditions: 0.5 equiv of each macrocycle, 1.2 equiv **3** and **4**, 0.96 equiv [Cu(MeCN)_4_](PF_6_), CH_2_Cl_2_, 100 °C (μW). R=(4‐^*t*^Bu‐C_6_H_4_)_3_C.

Initial experiments were performed with bulky azide **3** and alkyne **4** to assess the behavior of the macrocycles in the absence of dethreading. Under these conditions, macrocycle **1** produces a mixture of [2]rotaxane **5** and [3]rotaxane **7** (Table 1, entry 1). By contrast, macrocycle **2 a** produces almost exclusively [2]rotaxane **6 a** with only trace quantities of [3]rotaxane **8 a** (entry 2), whereas macrocycles **2 b** (entry 3) and **2 c** (entry 4) only form the singly interlocked product. To account for the high yield of [3]rotaxane in the case of macrocycle **1**, we previously proposed a mechanistic pathway involving dinuclear reactive intermediates **A** and **B** (Scheme 1), in which one macrocyclic bipyridine coordinates to Cu(π) and the other to Cu(σ), with bridging O‐Cu interactions stabilizing the assembly.^25^, ^26^ The failure of macrocycles **2** to form significant quantities of [3]rotaxane was ascribed to the lack of Cu−O interactions to stabilize doubly threaded intermediates (**2 a**) and the inability of smaller macrocycles to coordinate Cu(π) in a threaded manner (**2 b** and **2 c**).

**Table 1:** Product distribution in the mixed‐macrocycle AT‐CuAAC reaction.^[a,b]^

Entry	Macrocycles	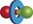	:	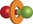	:	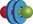	:	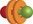	:	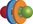	:	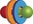
		**5**		**6**		**7**		**8**		**9**		**10**
1	**1** only	52	:	–	:	48	:	–	:	–	:	–
2	**2 a** only	–		98		–		2.0		**–**		–
3	**2 b** only	–		100		–		–		**–**		–
4	**2 c** only	–		100		–		–		**–**		–
5	**1**+**2 a**	35	:	47	:	13	:	0.53	:	3.2	:	1.4
6^[c]^	**1**+**2 a**	22	:	20	:	4.3	:	<0.25	:	2.0	:	1.0
7	**1**+**2 b**	34	:	54	:	5.6	:	–	:	6.7	:	–
8	**1**+**2 c**	33	:	49	:	8.3	:	–	:	9.7	:	–

[a] Reagents and conditions as in Scheme 1. [b] Ratios determined by ^1^H NMR analysis of crude reaction mixtures. [c] Competition experiment with 0.6 equiv each of **3** and **4**; balance of material is recovered **1** and **2 a**.

When macrocycles **1** and **2 a** were employed together (entry 5), a complex mixture was produced consisting of all possible interlocked products. Careful ^1^H NMR analysis of the crude mixture revealed a number of key points. First, under these conditions, simple [2]rotaxanes **5** and **6 a** were formed in unequal quantities, possibly indicating a difference in reactivity between **1** and **2 a**. However, when the reaction was run under competition conditions (entry 6), [2]rotaxanes **5** and **6 a** were formed in near‐equal amounts, suggesting that the [2]rotaxane pathway is equally favored for both macrocycles. Second, although macrocycle **2 a** alone forms only trace quantities of [3]rotaxane **8 a**, in the presence of **1**, **2 a** is competitively recruited into the [3]rotaxane pathway and the combined yield of heterocircuit rotaxanes **9 a** and **10 a** is of the same order of magnitude as homocircuit [3]rotaxane **7**. Finally, heterocircuit rotaxanes **9** and **10** were formed in an unequal ratio of isomers, suggesting that there is a significant preference in the position macrocycles **1** and **2 a** occupy in the mixed‐macrocycle equivalents of intermediates **A** and **B**. Careful chromatography allowed the major isomer to be isolated and identified by ROESY NMR as [3]rotaxane **9 a** (Supporting Information, Figure S3), suggesting that the intermediate in which macrocycle **1** coordinates to Cu(π) is favored.

In keeping with the inability of **2 b** to coordinate Cu(π), the reaction of **1** with **2 b** (entry 7) led to a simpler product mixture containing [2]rotaxanes **5** and **6 b**, homocircuit product **7** and single heterocircuit [3]rotaxane isomer **9 b**. Finally, when **1** and **2 c** are employed (entry 8), both of which contain the key benzylic ether unit, the quantity of the sole heterocircuit stereoisomer **9 c** increases significantly, further suggesting that the heterocircuit pathway is enhanced when both macrocycles contribute stabilizing Cu−O interactions. Chromatography allowed **9 c** to be isolated in 20 % yield based on **1** and the stereochemistry confirmed by ROESY NMR (Supporting Information, Figure S5).

Given the complete stereoselectivity and reasonable yield of doubly interlocked product **9 c** observed in the reaction of macrocycles **1** and **2 c**, we selected this combination for investigation under self‐sorting conditions. As the sole heterocircuit product formed in the reaction between azide **3** and alkyne **4** is that in which larger macrocycle **1** is situated on the side of the axle derived from the alkyne component, we investigated the reaction of macrocycles **1** and **2 c** in the presence of alkyne **4** and smaller azides **11** (Scheme 2).

**Scheme 2:**
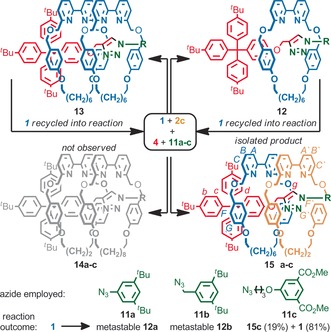
Effect of azide structure in the self‐sorting synthesis of [3]rotaxanes **15**. Conditions as in Scheme 1. Ratios determined by ^1^H NMR analysis of crude reaction mixtures. In all cases, the balance of **2 c** is converted quantitatively into the corresponding [2]rotaxane.

Although molecular modeling^27^ indicated that the 3,5‐di‐^*t*^Bu‐benzene moiety is smaller than the internal cavity of macrocycle **1**, the reaction of azides **11 a** or **11 b** with alkyne **4** and macrocycles **1** and **2 c** led to complete consumption of macrocycle **1** to give metastable [2]rotaxanes **12 a** and **12 b** respectively, which slowly reverted into macrocycle **1** and the non‐interlocked axle. No doubly interlocked products were isolated, suggesting these axles are too hindered to incorporate two macrocycles.^28^ Pleasingly, when azide **11 c** was employed which is both more flexible and less bulky, ^1^H NMR analysis of the crude reaction mixture (Figure 2 b) revealed only the expected products: [3]rotaxane **15 c** (19 %) and recovered **1** (81 %), alongside the corresponding non‐interlocked thread, and the [2]rotaxane of macrocycle **2 c**. [3]Rotaxane **14 c** was not observed. The order of the macrocyclic components on the axle of **15 c** was again confirmed unambiguously by ROESY NMR analysis (Supporting Information, Figure S6).^29^

**Figure 2:**
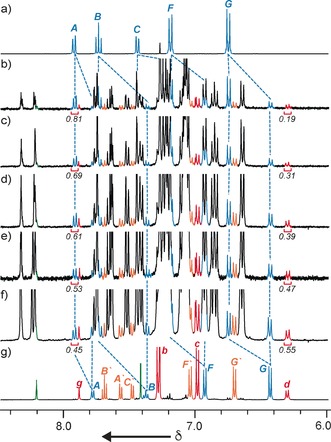
Partial ^1^H NMR stack plot (400 MHz, 298 K, CDCl_3_) with selected signals assigned and integrated of a) macrocycle **1**, g) heterocircuit rotaxane **15 c**, and b)–f) the crude product mixtures after 1–5 rounds of AT‐CuAAC coupling, respectively. See Scheme 2 for labeling.

The numerical similarity between the conversions of **1** into heterocircuit [3]rotaxanes **9 c** (20 %, Table 1, entry 8) and **15 c** (19 %) suggests that significant de‐threading of **1** from pseudorotaxanes **12 c** and **13 c**, and thus self‐sorting, does not take place competitively on the time scale of the AT‐CuAAC reaction.^30^ However, when an additional portion of **2 c**, **4**, **11 c** and Cu^I^ was added and the AT‐CuAAC reaction repeated, ^1^H NMR analysis indicated the conversion of **1** to **15 c** increased to 31 % (Figure 2 c; Figure 3), demonstrating that macrocycle **1** is indeed released from products **12 c** and **13 c** to be recycled into the reaction network. In this manner, over three further iterations (Figure 2 d–f; Figure 3), the conversion of **1** into [3]rotaxane **15 c** was increased to 55 %, demonstrating that self‐sorting is indeed in operation, increasing the conversion of **1** to a sole interlocked product. Finally, we compared our iterative self‐sorting method with the analogous all‐in‐one reaction. Repeating the AT‐CuAAC reaction of **1** in the presence of an excess **2 c**, **4** and **11** led to 28 % yield of **1** to target [3]rotaxane **15 c**, demonstrating the enhanced efficiency of the iterative kinetic self‐sorting process.

**Figure 3:**
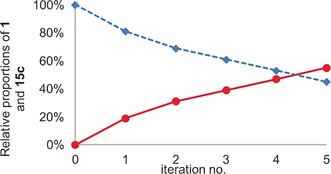
Relative proportions of macrocycle **1** (blue) and [3]rotaxane **15 c** (red) in the crude reaction mixture as a function of reaction iteration.

In conclusion, by taking advantage of the bimetallic mechanism of the AT‐CuAAC reaction, two different macrocycles can be incorporated into a [3]rotaxane product. Furthermore, by judicious choice of macrocycles, a single stereoisomer of the possible heterocircuit products is formed exclusively, raising the synthetic utility of this reaction and lending significant weight to our previous mechanistic hypothesis.^25^ By adapting the reaction to include a self‐sorting element based on the kinetic stabilities of the possible products, the yield of this complex interlocked target can be selectively amplified. Challenges still remain; the yield of the heterocircuit target without self‐sorting remains low, albeit acceptable. Furthermore, although the self‐sorting concept has been demonstrated, the reaction becomes less efficient after the first round of AT‐CuAAC possibly due to Cu^I^ coordination hindering the escape of macrocycle **1**.^30^ Studies are on‐going to understand and address these remaining hurdles. Having demonstrated the potential for kinetic self‐sorting in AT reactions we believe that there is significant potential to develop novel synthetic approaches that harness the detailed mechanisms of AT couplings and/or similar self‐sorting processes.^31^ These approaches may find wider application in the synthesis of complex, multicomponent interlocked products through the application of reactions in which two, or perhaps more, distinguishable catalytic centers are involved in the key covalent bond forming step.

## Experimental Section

General procedure for the synthesis of heterocircuit [3]rotaxanes **10** and **15 c**: Macrocycle **1** (0.5 equiv), macrocycle **2** (0.5 equiv), azide (1.20 equiv), alkyne (1.20 equiv), and [Cu(MeCN)_4_]PF_6_ (0.96 equiv) were dissolved in CH_2_Cl_2_ and the solution was stirred at 100 °C (under microwave radiation) for 2 h. The solution was allowed to return to RT, diluted with CH_2_Cl_2_ (50 mL), and washed with NH_3_–EDTA_aq_. The aqueous layer was extracted with CH_2_Cl_2_. The organic extracts were combined, dried (MgSO_4_), and the volume reduced in vacuo.

## Supporting information

As a service to our authors and readers, this journal provides supporting information supplied by the authors. Such materials are peer reviewed and may be re‐organized for online delivery, but are not copy‐edited or typeset. Technical support issues arising from supporting information (other than missing files) should be addressed to the authors.

Supplementary
