# Supplementary material for: A Kinetic Self‐Sorting Approach to Heterocircuit [3]Rotaxanes
Source: Angew Chem Int Ed Engl. 2016 Sep 7;55(40):12488–93. doi: 10.1002/anie.201606640 (PMC5113769; doi:10.1002/anie.201606640)
Supplement: Supplementary file 1 — Supplementary [file ANIE-55-12488-s001.pdf]

Angewandte Chemie
Eine Zeitschrift der Gesellschaft Deutscher Chemiker

Supporting Information

A Kinetic Self-Sorting Approach to Heterocircuit [3]Rotaxanes

*Edward A. Neal and Stephen M. Goldup**

anie_201606640_sm_miscellaneous_information.pdf

Supporting Information

Contents

1. General Experimental Information 2
2. General Experimental Procedures 2
3. Experimental Procedures 3
4. Development of the Kinetic Self-Sorting Reaction 16
5. ROESY NMR Analysis of Heterocircuit [3]Rotaxanes 21
6. Characterisation Data 25
7. References 83

1. General Experimental Information

Unless otherwise stated, all reagents were purchased from commercial sources and used without further purification. All reactions were carried out under an atmosphere of N_2 using anhydrous solvents unless otherwise stated. Anhydrous THF, toluene, DMF, diethyl ether and methylene chloride were purchased from Acros. Otherwise solvents were used without further purification. Microwave-assisted reactions were undertaken on a CEM Discover SP reactor. Flash column chromatography was performed using a Biotage Isolera 4 automated chromatography system, employing Biotage ZIP or SNAP KP-SIL cartridges. Analytical NMR spectra were recorded on Bruker AV400, AVIII400, AVII400, AVIIIHD400, AVIIIHD500 or AV600 instrument, at a constant temperature of 300 K. ^{13}C-NMR were recorded as JMOD experiments (phased spectrum including quaternary Cs) or UDEFT (uniform driven equilibrium Fourier transform for quaternary enhancement). Chemical shifts are reported in parts per million from low to high field and referenced to residual solvent. Coupling constants (J) are reported in Hz. Standard abbreviations indicating multiplicity were used: s = singlet, d = doublet, t = triplet, q = quartet, quin. = quintet, m = multiplet, br = broad, app = apparent. All melting points were determined using a Sanyo Gallenkamp apparatus. Low and high resolution mass spectrometry was carried out by the mass spectrometry service at the University of Southampton using either a MaXis (Bruker Daltonics, Bremen, Germany) mass spectrometer equipped with a Time of Flight (TOF) analyser or solariX (Bruker Daltonics, Bremen, Germany) FT-ICR mass spectrometer equipped with a 4.7T superconducting magnet.

Petrol refers to the fraction of petroleum ether boiling in the range 40-60 °C. NH_3-EDTA refers to a saturated solution of the tetrasodium salt of ethylene diamine tetracarboxylic acid in 16% $NH_{3(aq)}$.

Macrocycle **1**,[1] macrocycle **2a**,[2] macrocycle **2b**,[3] macrocycle **2c**,[1] azide **3**,[4] alkyne **4**,[4] [2]rotaxane **5**,[1] [2]rotaxane **6a**,[2] [2]rotaxane **6b**,[3] [2]rotaxane **6c**,[1] [3]rotaxane **7**,[2] azide **11a**,[5] azide **11b**[3, 6] and thread **S1**[7] were synthesised according to literature procedures.

2. General Experimental Procedures

General Heterocircuit AT-CuAAC Rotaxanation Method

Macrocycle **1** (0.5 eq.), macrocycle **2** (0.5 eq.), azide (1.20 eq.), alkyne (1.20 eq.) and $[Cu(MeCN)_4]PF_6$ (0.96 eq.) were dissolved in CH_2Cl_2 and the resulting solution was stirred at 100 °C in a 150 W microwave reactor for 2 h. The solution was allowed to return to room temperature, diluted with further CH_2Cl_2 (50 mL), washed with NH_3-EDTA (50 mL). The organic layer was retained and the aqueous layer extracted with CH_2Cl_2 (2 × 50 mL portions). The organic extracts were combined, dried over $MgSO_4$ and reduced *in vacuo*.

[2]Rotaxane Formation Method

Alkyne (1.20 eq.), azide (1.20 eq.), $[Cu(MeCN)_4]PF_6$ (0.96 eq.), NEt^iPr_2 (4.8 µL) and macrocycle (1.00 eq.) were dissolved in THF and the resulting solution was stirred at 30 °C for 24 h. The solution was allowed to return to room temperature, diluted with CH_2Cl_2 (50 mL) and washed with NH_3-EDTA (50 mL). The organic layer was retained and the aqueous layer extracted with CH_2Cl_2 (2 × 50 mL). The organic extracts were combined, dried over $MgSO_4$ and reduced *in vacuo*.

Thread Formation Method

Alkyne (1.20 eq.), azide (1.20 eq.), $[Cu(MeCN)_4]PF_6$ (0.96 eq.) and N^iPr_2Et (4.8 µL) was dissolved in THF and the resulting solution was stirred at 30 °C for 1 hr. The solution was allowed to return to room temperature, diluted with CH_2Cl_2 (50 mL) and washed with NH_3-EDTA (50 mL). The organic layer was retained and the aqueous layer extracted with CH_2Cl_2 (2 × 50 mL). The organic extracts were combined, dried over $MgSO_4$ and reduced *in vacuo*.

3. Experimental Procedures

Synthesis of Heterocircuit [3]Rotaxane 9a

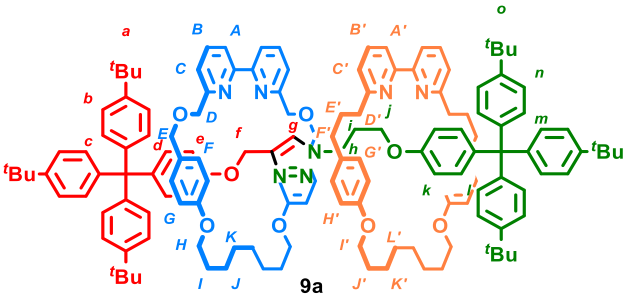

9a

[3]Rotaxane **9a** was isolated according to general heterocircuit rotaxane conditions with a deficit of half-thread components (competition conditions; table 1, entry 6) using macrocycle **1** (53.9 mg, 0.100 mmol), macrocycle **2a** (53.5 mg, 0.100 mmol), azide **3** (70.5 mg, 0.120 mmol), alkyne **4** (64.1 mg, 0.120 mmol) and $[Cu(MeCN)_4]PF_6$ (35.8 mg, 0.096 mmol), in CH_2Cl_2 (5 mL). Flash column chromatography (0-30% MeCN/1:1 hexane:CH_2Cl_2 (+0.25% EtOH)) afforded 2.6 mg (2.2%) of [3]rotaxane **9a** as a white foam: ^{1}H NMR (600 MHz, $CDCl_3$) δ ppm 7.88 (d, *J*=7.8, 2H, $H_{A'}$), 7.80 (d, *J*=7.8, 2H, H_A), 7.41 (t, *J*=7.8, 2H, H_B), 7.43 (t, *J*=7.8, 2H, $H_{B'}$), 7.31 (d, *J*=7.8, 2H, H_C), 7.23 - 7.29 (m, 12H, H_b and H_n), 7.05 (d, *J*=8.5, 12H, H_c and H_m), 7.03 (s, 1H, H_g), 6.96 (d, *J*=7.6, 2H, $H_{C'}$), 6.92 (d, *J*=8.5, 4H, H_F), 6.58 - 6.65 (m, 8H, $H_{G'}$, H_d and H_l), 6.49 (d, *J*=8.5, 4H, H_G), 6.42 (d, *J*=8.7, 4H, $H_{H'}$), 5.82 (d, *J*=8.9, 2H, H_e), 5.78 (d, *J*=8.9, 2H, H_k), 4.46 - 4.60 (m, 8H, H_D and H_E), 4.21 (s, 2H, H_f), 3.62 - 3.81 (m, 8H, H_H and $H_{I'}$), 3.58 (m, *J*=6.0, 2H, H_h), 2.78 - 2.88 (m, 6H, $H_{D'}$ and H_j), 2.44 (t, *J*=8.1, 4H, $H_{F'}$), 1.70 - 1.88 (m, 4H, $H_{E'}$), 1.47 - 1.61 (m, 8H, H_I and $H_{J'}$), 1.18 - 1.39 (m, 8H, H_J and $H_{K'}$), 1.33 (s, 27H, H_a or H_o), 1.34 (s, 27H, H_a or H_o), 1.09 - 1.18 (m, 10H, H_i, H_J and H_K); ^{13}C NMR (150 MHz, $CDCl_3$) δ ppm 161.8 (C), 158.8 (C), 158.6 (C), 157.2 (C), 156.8 (C), 156.0 (C), 156.0 (C), 155.5 (C), 148.3 (C), 148.3 (C), 146.1 (C), 144.5 (C), 144.5 (C), 142.5 (C), 139.0 (C), 137.0 (CH), 137.0 (CH), 134.2 (C), 131.7 (CH), 131.6 (CH), 131.0 (CH), 130.9 (CH), 129.8 (CH), 129.5 (C), 129.1 (CH), 124.2 (CH), 124.2 (CH), 123.1 (CH), 122.5 (CH), 120.8 (CH), 119.9 (CH), 119.2 (CH), 114.7 (CH), 114.7 (CH), 112.8 (CH), 112.8 (CH), 72.5 (CH_2), 71.6 (CH_2), 67.8 (CH_2), 67.8 (CH_2), 63.7 (CH_2), 63.5 (C), 63.5 (C), 61.2 (CH_2), 47.1 (CH_2), 38.3 (CH_2), 34.5 (C), 34.5 (C), 34.4 (CH_2), 32.9 (CH_2), 31.6 (CH_3), 31.6 (CH_3), 29.9 (CH_2), 29.5 (CH_2), 29.2 (CH_2), 28.9 (CH_2), 28.9 (CH_2), 26.0 (CH_2), 25.9 (CH_2); LRMS (ESI+) 1102.2 m/z $[M+2H]^{2+}$.

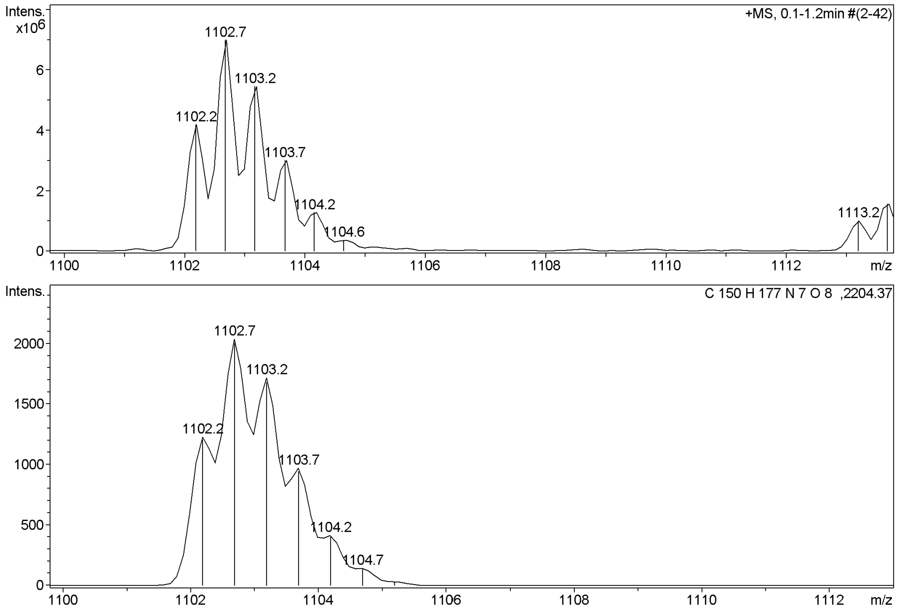

Synthesis of Heterocircuit [3]Rotaxane 9b

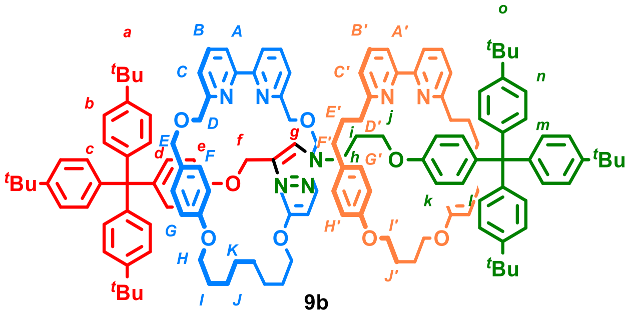

9b

[3]Rotaxane **9b** was made according to the general heterocircuit rotaxane conditions, using macrocycle **1** (27.0 mg, 0.050 mmol), macrocycle **2b** (23.8 mg, 0.050 mmol), azide **3** (70.5 mg, 0.120 mmol), alkyne **4** (64.1 mg, 0.120 mmol) and $[Cu(MeCN)_4]PF_6$ (17.9 mg, 0.048 mmol), in CH_2Cl_2 (2.5 mL). Flash column chromatography (0-30% MeCN/1:1 hexane:CH_2Cl_2 (+0.25% EtOH)) afforded 1.6 mg (1.5 %) of [3]rotaxane **9b** as a white foam: ^{1}H NMR (500 MHz, $CDCl_3$) δ ppm 7.96 (s, 1H, H_g), 7.73 (d, *J*=7.8, 2H, H_A), 7.51 (d, *J*=7.8, 2H, $H_{B'}$), 7.34 (d, *J*=7.8, 2H, $H_{A'}$), 7.31 (d, *J*=7.8, 2H, H_B), 7.23 - 7.28 (m, 14H, H_C, H_b and H_n), 7.10 (d, *J*=8.6, 6H, H_c or H_m), 7.05 (d, *J*=7.8, 2H, $H_{C'}$), 6.95 (d, *J*=8.9, 6H, H_c or H_m), 6.89 (d, *J*=8.6, 4H, H_F), 6.86 (d, *J*=8.9, 2H, H_d), 6.74 (d, *J*=8.6, 4H, $H_{G'}$), 6.62 (d, *J*=8.6, 4H, $H_{H'}$), 6.40 (d, *J*=8.8, 4H, H_G), 6.26 (d, *J*=8.9, 2H, H_e), 6.26 (d, *J*=9.0, 2H, H_l), 5.37 (d, *J*=9.0, 2H, H_k), 4.40 - 4.55 (m, 8H, H_D and H_E), 4.22 - 4.30 (m, 2H, one of $H_{I'}$), 4.16 (s, 2H, H_f), 3.97 - 4.05 (m, 2H, one of $H_{I'}$), 3.45 – 3.66 (m, 6H, H_H and H_h), 3.17 (t, *J*=6.1, 2H, H_j), 2.61 – 2.69 (m, 2H, one of $H_{D'}$), 2.45 – 2.60 (m, 6H, one of $H_{D'}$ and $H_{F'}$), 1.60 – 2.16 (m, 8H, $H_{E'}$ and $H_{J'}$), 1.00 – 1.40 (m, 6H, H_I and H_i), 1.34 (s, 27H, H_a or H_o), 1.31 (s, 27H, H_a or H_o), 0.65 – 1.00 (m, 8H, H_J and H_K); ^{13}C NMR (125 MHz, $CDCl_3$) δ ppm 162.8 (C), 158.7 (C), 158.4 (C), 158.0 (C), 157.6 (C), 156.5 (C), 155.7 (C), 155.2 (C), 148.4 (C), 148.0 (C), 144.6 (C), 144.5 (C), 142.7 (C), 138.7 (C), 138.4 (C), 136.9 (CH), 136.8 (CH), 133.3 (C), 131.8 (CH), 131.3 (CH), 131.0 (CH), 130.9 (CH), 129.9 (CH), 129.6 (C), 129.6 (CH), 124.2 (CH), 124.2 (CH), 124.1 (CH), 121.5 (CH), 120.5 (CH), 120.1 (CH), 119.6 (CH), 115.2 (CH), 114.6 (CH), 113.1 (CH), 112.4 (CH), 72.3 (CH_2), 71.2 (CH_2), 67.6 (CH_2), 66.7 (CH_2), 64.4 (CH_2), 63.2 (C), 63.0 (C), 61.2 (CH_2), 46.8 (CH_2), 37.6 (CH_2), 35.1 (C), 35.1 (C), 33.0 (CH_2), 33.6 (CH_2), 31.7 (CH_3), 31.6 (CH_3), 29.5 (CH_2), 29.2 (CH_2), 28.9 (CH_2), 25.8 (CH_2), 25.0 (CH_2); LRMS (ESI+) 1074.2 m/z $[M+2H]^{2+}$.

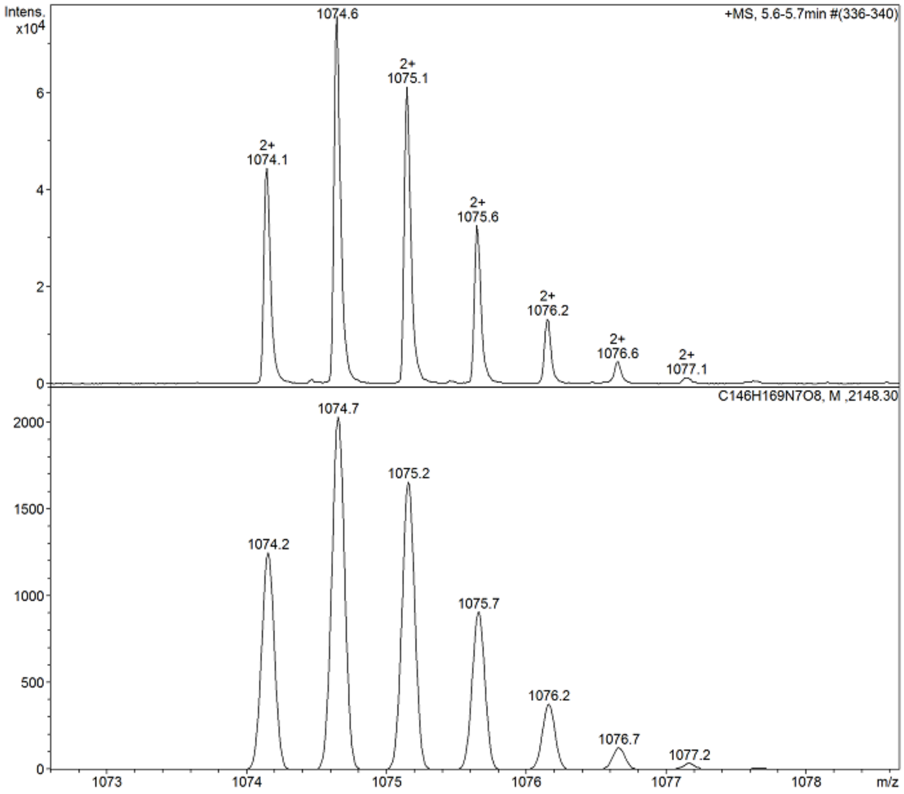

Synthesis of Heterocircuit [3]Rotaxane 9c

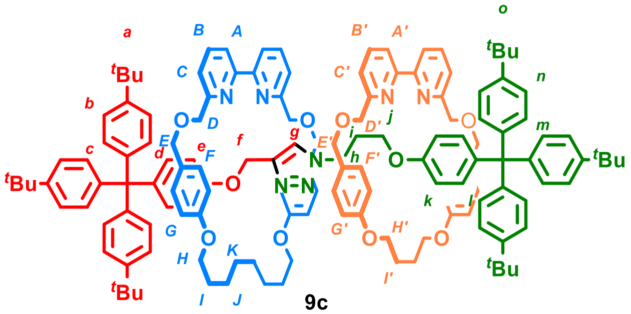

9c

[3]Rotaxane **9c** was made according to the general heterocircuit rotaxane conditions, using macrocycle **1** (27.0 mg, 0.050 mmol), macrocycle **2c** (24.1 mg, 0.050 mmol), azide **3** (70.5 mg, 0.120 mmol), alkyne **4** (64.1 mg, 0.120 mmol) and $Cu(MeCN)_4.PF_6$ (17.9 mg, 0.048 mmol), in CH_2Cl_2 (2.5 mL). Flash column chromatography (0-30% MeCN/1:1 hexane:CH_2Cl_2 (+0.25% EtOH)) afforded11.6 mg (20%) of [3]rotaxane **9c** as a white foam: ^{1}H NMR (600 MHz, $CDCl_3$) δ ppm 7.92 (s, 1H, H_g), 7.75 (d, *J*=7.8, 2H, H_A), 7.63 (d, *J*=7.8, 2H, $H_{B'}$), 7.51 (d, *J*=7.8, 2H, $H_{A'}$), 7.46 (d, *J*=7.8, 2H, $H_{C'}$), 7.36 (d, *J*=7.8, 2H, H_B), 7.20 - 7.26 (m, 14H, H_C, H_b and H_n), 7.12 (d, *J*=8.9, 6H, H_c or H_m), 6.96 (d, *J*=8.9, 6H, H_c or H_m), 6.91 - 6.95 (m, 10H, H_d, $H_{F'}$ and H_F), 6.64 (d, *J*=8.8, 4H, $H_{G'}$), 6.42 (d, *J*=8.8, 4H, H_G), 6.33 (d, *J*=.8, 2H, H_e), 6.26 (d, *J*=8.8, 2H, H_l), 5.35 (d, *J*=8.8, 2H, H_k), 4.40 - 4.64 (m, 12H, H_D, H_E and $H_{D'}$ or $H_{E'}$), 4.30 - 4.35 (m, 2H, one of $H_{H'}$), 4.17 – 4.30 (m, 4H, $H_{D'}$ or $H_{E'}$), 4.19 (s, 2H, H_f), 4.00 – 4.07 (m, 2H, one of $H_{H'}$), 3.59 – 3.64 (m, 2H, one of H_H), 3.46 – 3.51 (m, 2H, one of H_H), 3.31 – 3.36 (m, 2H, H_h), 3.04 (t, *J*=6.6, 2H, H_j), 2.03 – 2.11 (m, 2H, one of $H_{I'}$), 1.89 – 1.96 (m, 2H, one of $H_{I'}$), 1.29 – 1.39 (m, 4H, H_I), 1.35 (s, 27H, H_a or H_o), 1.31 (s, 27H, H_a or H_o), 0.76 – 1.00 (m, 10H, H_J, H_K and H_i); ^{13}C NMR (125 MHz, $CDCl_3$) δ ppm 159.3 (C), 159.1 (C), 158.7 (C), 158.5 (C), 156.5 (C), 155.7 (C), 155.2 (C), 154.3 (C), 148.4 (C), 148.1 (C), 144.6 (C), 144.5 (C), 142.7 (C), 139.0 (C), 138.4 (C), 137.4 (CH), 136.8 (CH), 131.9 (CH), 131.3 (CH), 131.0 (CH), 130.9 (CH), 130.5 (CH), 130.0 (CH), 129.6 (C), 129.0 (C), 124.2 (CH), 124.2 (CH), 124.1 (CH), 121.3 (CH), 120.1 (CH), 120.0 (CH), 119.6 (CH), 115.3 (CH), 114.6 (CH), 113.1 (CH), 112.3 (CH), 73.0 (CH_2), 72.4 (CH_2), 71.1 (CH_2), 71.0 (CH_2), 67.6 (CH_2), 66.8 (CH_2), 64.4 (CH_2), 63.2 (C), 63.0 (C), 61.3 (CH_2), 46.5 (CH_2), 34.5 (2 x C), 31.7 (CH_3), 31.6 (CH_3), 29.9 (CH_2), 29.2 (CH_2), 28.9 (CH_2), 25.8 (CH_2), 24.9 (CH_2); LRMS (ESI+) 1076.1 m/z $[M+2H]^{2+}$.

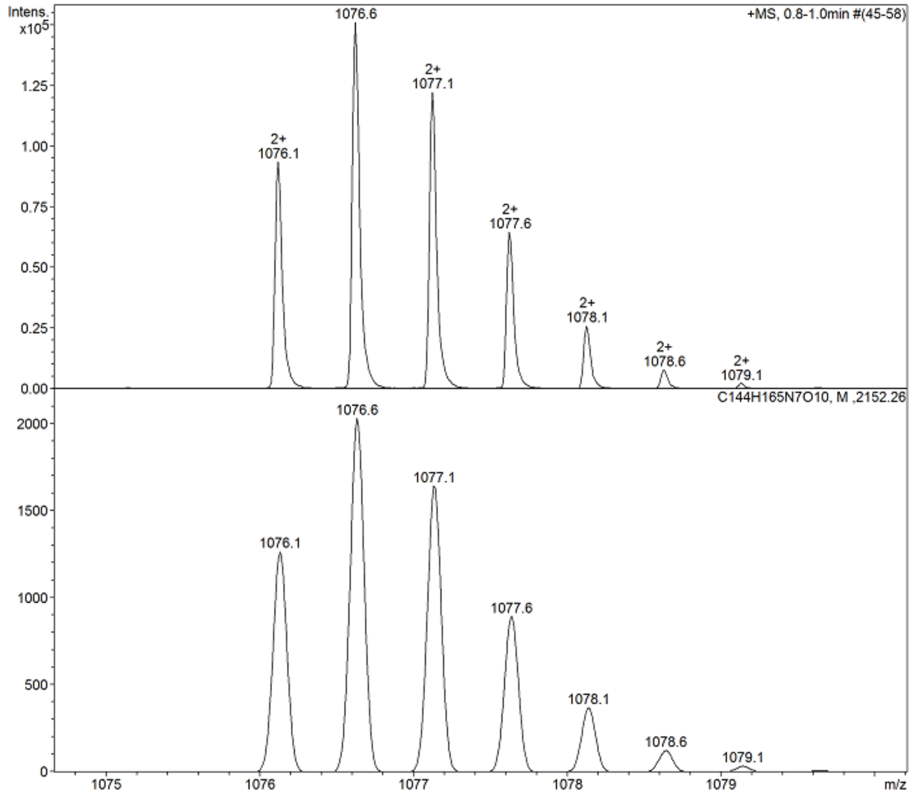

Dimethyl 5-(3-bromopropoxy)isophthalate (S14)

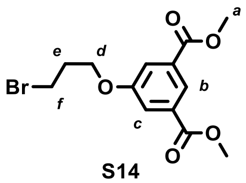

S14

A solution of dimethyl 5-hydroxyisophthalate (**S9**, 2.10 g, 10 mmol), 1,3-dibromopropane (6.06 g, 30 mmol) and K_2CO_3 (4.15 g, 30 mmol) in acetone (100 mL) was stirred at reflux for 16 h. The suspension was allowed to cool before dilution with H_2O (100 mL) and extraction with CH_2Cl_2 (3 x 100 mL). The organics were combined, dried ($MgSO_4$) and reduced *in vacuo*. Column chromatography (0-20% EtOAc/Petrol) afforded the target material (1.88g, 57%) as a white solid: m.p. 60-62 °C; ^{1}H NMR (500 MHz, $CDCl_3$) δ ppm 8.29 (t, *J*=1.5, 1H, H_b), 7.76 (d, *J*=1.5, 2H, H_c), 4.20 (t, *J*=5.8, 2H, H_d), 3.94 (s, 6H, H_a), 3.62 (t, *J*=6.4, 2H, H_f), 2.35 (app. quin, *J*=6.1, H_e); ^{13}C NMR (125 MHz, $CDCl_3$) δ ppm 166.2 (C), 159.0 (C), 132.0 (C), 123.4 (CH), 120.0 (CH), 66.0 (CH_2), 52.6 (CH_3), 32.3 (CH_2), 29.8 (CH_2); HRMS (ESI+) m/z 352.9994 $[M+Na]^+$ (calc. for $C_{13}H_{15}BrNaO_5$ 352.9995).

Dimethyl 5-(3-azidopropoxy)isophthalate (11c)

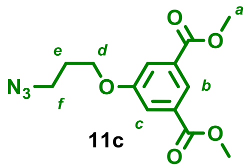

11c

A solution of dimethyl 5-(3-bromopropoxy)isophthalate (**S14**, 1.80 g, 5.4 mmol) and NaN_3 (0.70 g, 10.8 mmol, 2 eq.) in DMF (3 mL) was stirred at 80 °C for 8 h. The solution was diluted with H_2O (50 mL) and extracted with CH_2Cl_2 (50 mL). The organic layer was dried ($MgSO_4$) and reduced *in vacuo* to afford a pale yellow oil. Column chromatography (0-50% EtOAc/Petrol) afforded the target material (1.44 g, 90%) as a colourless oil: ^{1}H NMR (500 MHz, $CDCl_3$) δ ppm 8.29 (t, *J*=1.5, 1H, H_b), 7.75 (d, *J*=1.5, 2H, H_c), 4.14 (t, *J*=5.9, 2H, H_d), 3.94 (s, 6H, H_a), 3.54 (t, *J*=6.6, 2H, H_f), 2.08 (app. quin, *J*=6.3, H_e); ^{13}C NMR (125 MHz, $CDCl_3$) δ ppm 166.2 (C), 158.9 (C), 132.0 (C), 123.4 (CH), 119.9 (CH), 65.3 (CH_2), 52.6 (CH_3), 48.2 (CH_2), 28.8 (CH_2); HRMS (ESI+) m/z 316.0906 $[M+]^+$ (calc. for $C_{13}H_{15}N_3NaO_5$ 316.0904).

Synthesis of [2]Rotaxane S3

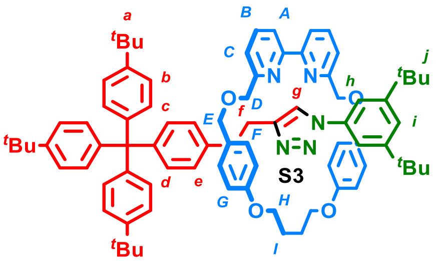

[2]Rotaxane **S3** was made according to the general [2]rotaxane synthesis conditions, using alkyne **4** (16.3 mg, 0.030 mmol), azide **11a** (6.9 mg, 0.030 mmol), macrocycle **2c** (11.3 mg, 0.025 mmol), $[Cu(MeCN)_4]PF_6$ (0.9 mg, 2.5 µmol) and N^iPr_2Et (4.8 µL) in THF (0.5 mL). Flash column chromatography (0-30% MeCN/1:1 hexane:CH_2Cl_2 (+0.25% EtOH)) afforded the target (22.2 mg, 71%) as a white foam: ^{1}H NMR (500 MHz, $CDCl_3$) δ ppm 9.74 (s, 1H, H_g), 7.64 (t, J=7.8, 2H, H_B), 7.59 (d, J=1.6, 2H, H_h), 7.54 (d, J=7.8, 2H, H_A), 7.40 (d, J=7.8, 2H, H_C), 7.30 (t, J=1.6, 1H, H_i), 7.24 (d, J=8.7, 6H, H_b), 7.08 (d, J=8.7, 6H, H_c), 6.85 (d, J=9.0, 2H, H_d), 6.72 (d, J=8.6, 4H, H_F), 6.55 (d, J=8.6, 4H, H_G), 6.36 (d, J=9.0, 2H, H_e), 4.27 - 4.49 (m, 8H, H_H and (H_D or H_E)), 4.24 (s, 2H, H_f), 3.92 - 4.06 (m, 4H, H_D or H_E), 2.10 - 2.16 (m, 4H, H_I), 1.31 (s, 27H, H_a), 1.21 (s, 18H, H_j); ^{13}C NMR (125 MHz, $CDCl_3$) δ ppm 159.5 (C), 159.1 (C), 156.5 (C), 155.7 (C), 152.0 (C), 148.3 (C), 144.5 (C), 143.1 (C), 138.9 (C), 137.2 (C), 137.2 (CH), 131.7 (CH), 130.9 (CH), 129.6 (CH), 128.4 (C), 124.6 (CH), 124.1 (CH), 121.4 (CH), 120.8 (CH), 120.2 (CH), 115.2 (CH), 114.7 (CH), 113.4 (CH), 73.0 (CH_2), 70.4 (CH_2), 66.7 (CH_2), 63.2 (C), 60.9 (CH_2), 35.2 (C), 34.4 (C), 31.6 (CH_3), 31.4 (CH_3), 24.9 (CH_2); LRMS (ESI+) m/z 1256.8 $[M+H]^+$.

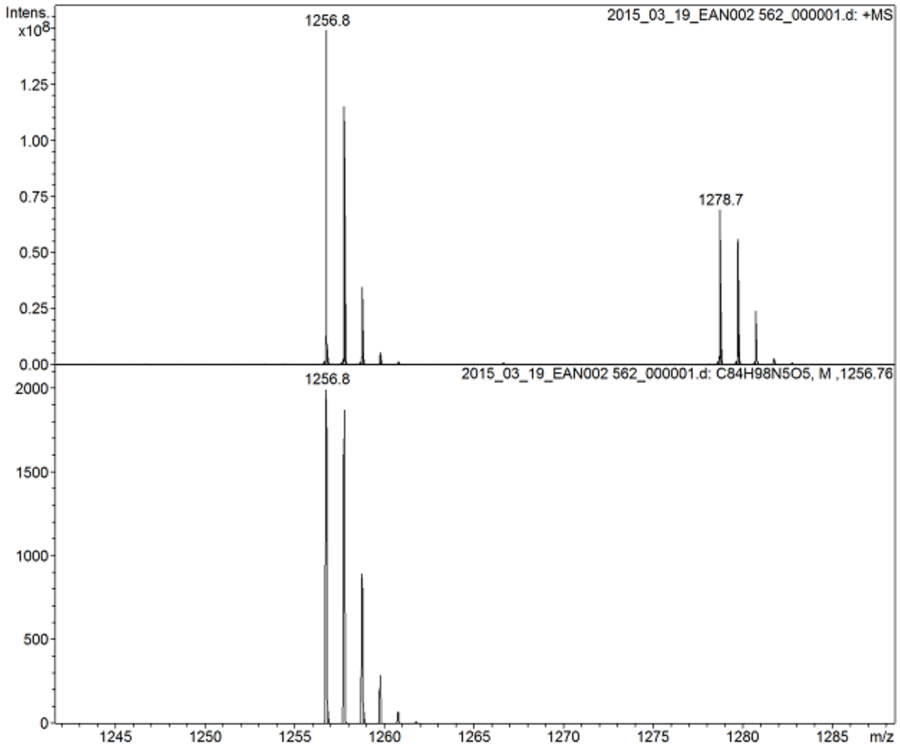

Synthesis of [2]Rotaxane S4

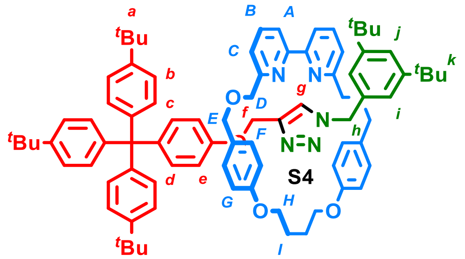

[2]Rotaxane **S4** was made according to the general [2]rotaxane synthesis conditions, using alkyne **4** (16.3 mg, 0.030 mmol), azide **11b** (7.4 mg, 0.030 mmol), macrocycle **2c** (11.3 mg, 0.025 mmol), $[Cu(MeCN)_4]PF_6$ (0.9 mg, 2.5 μmol) and N^iPr_2Et (4.8 μL) in THF (0.5 mL). Flash column chromatography (0-30% MeCN/1:1 hexane:CH_2Cl_2 (+0.25% EtOH)) afforded the target (21.6 mg, 68%) as a white foam: ^{1}H NMR (500 MHz, $CDCl_3$) δ ppm 8.31 (s, 1H, H_g), 7.57 (t, *J*=7.7, 2H, H_B), 7.46 (d, *J*=7.7, 2H, H_A), 7.39 (t, *J*=1.8, 1H, H_j), 7.37 (d, *J*=7.8, 2H, H_C), 7.24 (d, *J*=8.8, 6H, H_b), 7.14 (d, *J*=1.8, 2H, H_i), 7.06 (d, *J*=8.8, 6H, H_c), 6.74 (d, *J*=9.0, 2H, H_d), 6.69 (d, *J*=8.5, 4H, H_F), 6.48 (d, *J*=8.5, 4H, H_G), 6.18 (d, *J*=9.0, 2H, H_e), 5.13 (s, 2H, H_h), 4.25 - 4.49 (m, 6H, one of H_H and (H_D or H_E)), 4.00 (s, 2H, H_f), 3.93 - 4.14 (m, 6H, one of H_H and (H_D or H_E)), 1.85 - 2.10 (m, 4H, H_I), 1.31 (s, 27H, H_a), 1.24 (s, 18H, H_j); ^{13}C NMR (125 MHz, $CDCl_3$) δ ppm 159.4 (C), 159.1 (C), 156.1 (C), 156.0 (C), 151.5 (C), 148.2 (C), 144.6 (C), 143.5 (C), 138.4 (C), 137.0 (CH), 134.5 (C), 131.4 (CH), 130.9 (CH), 129.9 (CH), 128.6 (C), 125.2 (CH), 124.1 (CH), 123.2 (CH), 123.1 (CH), 120.6 (CH), 120.3 (CH), 115.3 (CH), 113.5 (CH), 72.8 (CH_2), 70.5 (CH_2), 66.8 (CH_2), 63.1 (C), 60.7 (CH_2), 54.3 (CH_2), 35.0 (C), 34.4 (C), 31.6 (CH_3), 31.5 (CH_3), 25.0 (CH_2); LRMS (ESI+) m/z 1270.8 $[M+H]^+$.

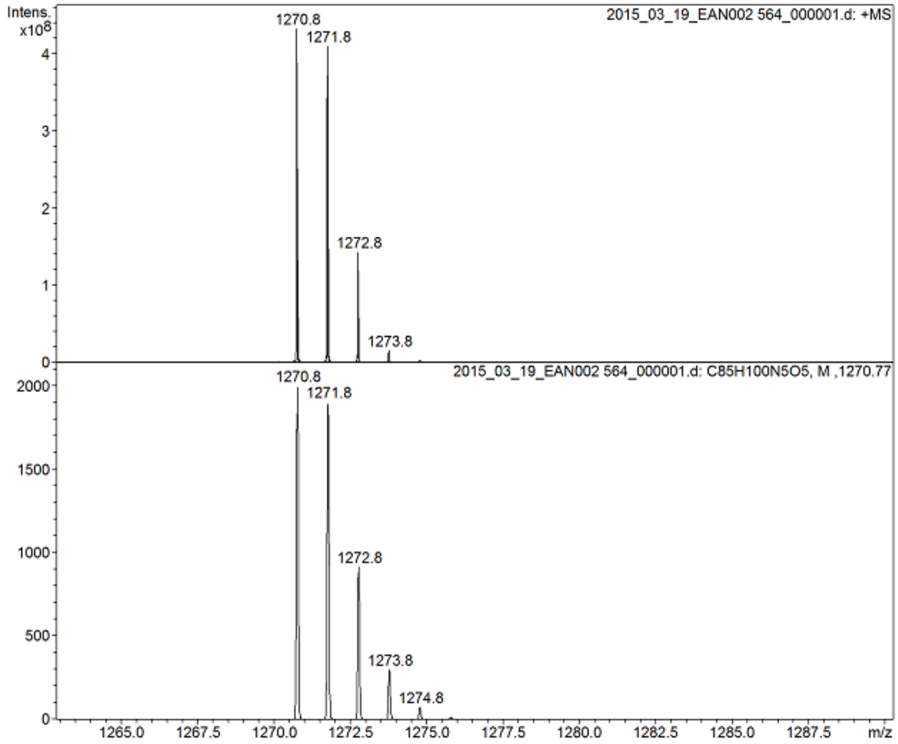

Synthesis of [2]Rotaxane S5

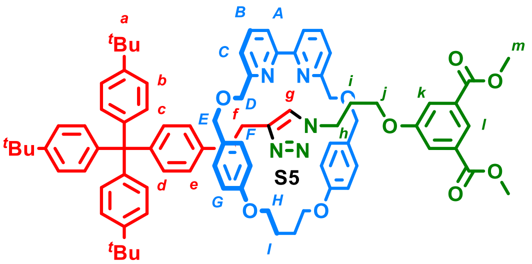

[2]Rotaxane **S5** was made according to the general [2]rotaxane synthesis conditions, using alkyne **4** (16.3 mg, 0.030 mmol), azide **11c** (8.8 mg, 0.030 mmol), macrocycle **2c** (11.3 mg, 0.025 mmol), $[Cu(MeCN)_4]PF_6$ (0.9 mg, 2.5 µmol) and N^iPr_2Et (4.8 µL) in THF (0.5 mL). Flash column chromatography (0-30% MeCN/1:1 hexane:CH_2Cl_2 (+0.25% EtOH)) afforded the target (29.3 mg, 89%) as a white foam: ^{1}H NMR (500 MHz, $CDCl_3$) δ ppm 8.22 (s, 1H, H_g), 8.20 (t, *J*=1.4, 1H, H_l), 7.64 (t, *J*=7.8, 2H, H_B), 7.51 (d, *J*=7.8, 2H, H_A), 7.44 (d, *J*=1.4, 2H, H_k), 7.41 (d, *J*=7.8, 2H, H_C), 7.20 (d, *J*=8.7, 6H, H_b), 7.09 (d, *J*=9.0, 2H, H_d), 7.06 (d, *J*=8.7, 6H, H_c), 6.93 (d, *J*=8.7, 4H, H_F), 6.87 (d, *J*=9.0, 2H, H_e), 6.65 (d, *J*=8.7, 4H, H_G), 5.06 (s, 2H, H_f), 4.29 - 4.61 (m, 6H, one of H_H and (H_D or H_E)), 4.14 - 4.28 (m, 4H, H_D or H_E), 3.99 - 4.09 (m, 2H, one of H_H), 3.95 (s, 6H, H_m), 3.17 - 3.23 (m, 2H, H_h), 3.13 (t, *J*=6.1, H_j), 1.87 - 2.13 (m, 4H, H_I), 1.29 (s, 27H, H_a), 0.95 - 1.03 (m, 2H, H_i); ^{13}C NMR (125 MHz, $CDCl_3$) δ ppm 166.3 (C), 159.2 (C), 158.8 (C), 158.8 (C), 156.5 (C), 156.4 (C), 148.4 (C), 144.2 (C), 142.8 (C), 140.0 (C), 137.3 (CH), 132.4 (CH), 131.5 (C), 130.9 (CH), 130.5 (CH), 129.2 (C), 125.1 (CH), 124.2 (CH), 122.7 (CH), 121.4 (CH), 121.1 (CH), 120.0 (CH), 115.1 (CH), 113.4 (CH), 73.0 (CH_2), 71.2 (CH_2), 66.7 (CH_2), 65.1 (CH_2), 63.2 (C), 61.7 (CH_2), 52.5 (CH_3), 46.2 (CH_2), 34.4 (C), 31.5 (CH_3), 28.3 (CH_2), 24.8 (CH_2); LRMS (ESI+) m/z 1318.7 $[M+H]^+$.

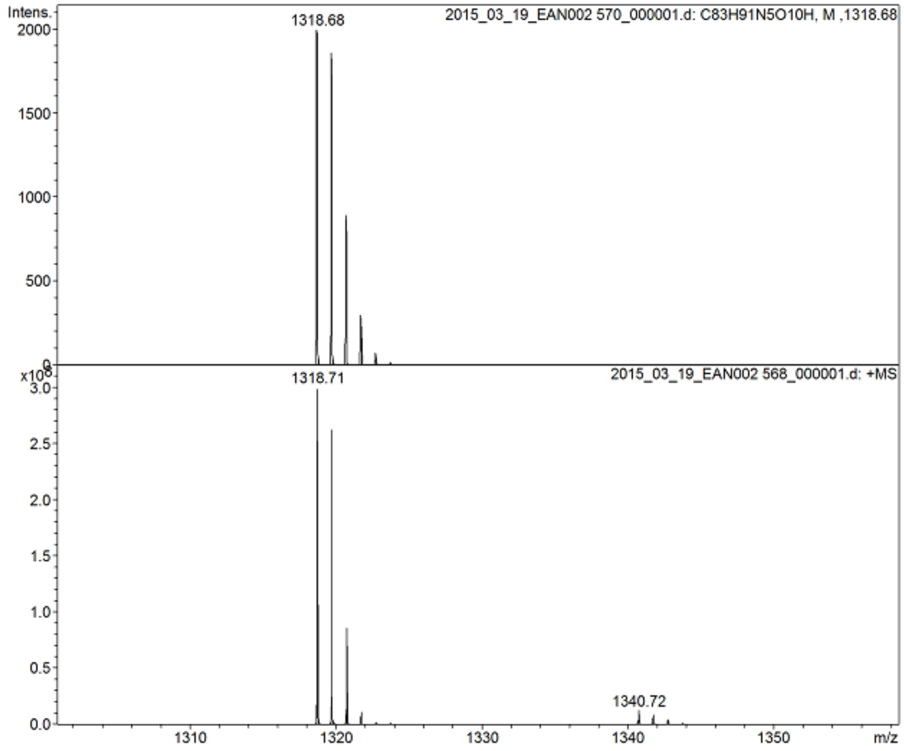

Synthesis of Thread Triazole S6

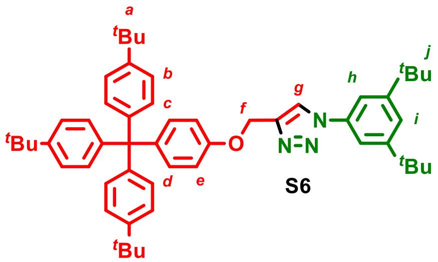

S6

Thread triazole **S6** was made according to the general thread triazole synthesis conditions, using alkyne **4** (16.3 mg, 0.030 mmol), azide **11a** (6.9 mg, 0.030 mmol), $[Cu(MeCN)_4]PF_6$ (0.9 mg, 2.5 µmol) and N^iPr_2Et (4.8 µL) in THF (0.5 mL). Flash column chromatography (0-30% MeCN/1:1 hexane:CH_2Cl_2 (+0.25% EtOH)) afforded the target (12.6 mg, 54%) as a white foam: ^1H NMR (500 MHz, $CDCl_3$) δ ppm 8.04 (s, 1H, H_i), 7.51 (app. s, 3H, H_h and H_g), 7.24 (d, *J*=8.4, 6H, H_b), 7.12 (d, *J*=9.0, 2H, H_d), 7.08 (d, *J*=8.4, 6H, H_c), 6.91 (d, *J*=9.0, 2H, H_e), 5.31 (s, 2H, H_f), 1.37 (s, 18H, H_j), 1.30 (s, 27H, H_a); ^{13}C NMR (125 MHz, $CDCl_3$) δ ppm 156.3 (C), 153.0 (C), 148.5 (C), 145.1 (C), 144.2 (C), 140.5 (C), 136.9 (C), 132.5 (CH), 130.9 (CH), 124.2 (CH), 123.3 (CH), 121.6 (CH), 115.8 (CH), 113.4 (CH), 63.2 (C), 62.3 (CH_2), 35.3 (C), 34.4 (C), 31.5 (CH_3), 31.5 (CH_3); HRMS (ESI+) m/z 774.5369 $[M+H]^+$ (calc. for $C_{54}H_{68}N_3O$ 774.5357).

Synthesis of Thread Triazole S7

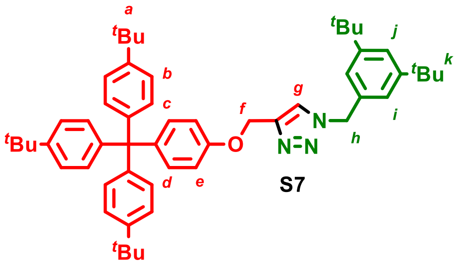

S7

Thread triazole **S7** was made according to the general thread triazole synthesis conditions, using alkyne **4** (16.3 mg, 0.030 mmol), azide **11b** (7.4 mg, 0.030 mmol), $[Cu(MeCN)_4]PF_6$ (0.9 mg, 2.5 µmol) and N^iPr_2Et (4.8 µL) in THF (0.5 mL). Flash column chromatography (0-30% MeCN/1:1 hexane:CH_2Cl_2 (+0.25% EtOH)) afforded the target (12.9 mg, 55%) as a white foam: ^1H NMR (500 MHz, $CDCl_3$) δ ppm 7.54 (br. s, 1H, H_g), 7.42 (t, *J*=1.5, 1H, H_j), 7.22 (d, *J*=8.6, 6H, H_b), 7.11 (d, *J*=1.5, 2H, H_i), 7.04 - 7.10 (m, 8H, H_d and H_c), 6.83 (d, *J*=9.0, 2H, H_e), 5.51 (s, 2H, H_h), 5.17 (s, 2H, H_f), 1.30 (s, 27H, H_a), 1.30 (s, 18H, H_k); ^{13}C NMR (125 MHz, $CDCl_3$) δ ppm 156.3 (C), 152.0 (C), 148.5 (C), 144.8 (C), 144.2 (C), 140.3 (C), 133.7 (C), 132.5 (CH), 130.9 (CH), 124.2 (CH), 123.0 (CH), 122.7 (CH), 122.6 (CH), 113.3 (CH), 63.2 (C), 62.3 (CH_2), 55.1 (CH_2), 35.0 (C), 34.4 (C), 31.5 (CH_3), 31.5 (CH_3); HRMS (ESI+) m/z 810.5344 $[M+Na]^+$ (calc. for $C_{55}H_{69}N_3NaO$ 810.5333).

Synthesis of Thread Triazole S8

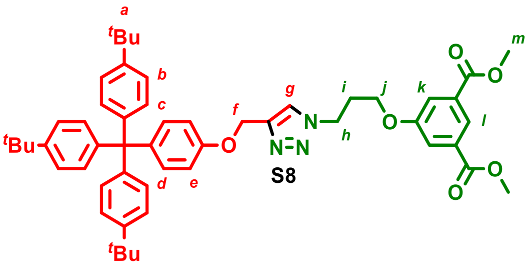

S8

Thread triazole **S8** was made according to the general thread triazole synthesis conditions, using alkyne **4** (16.3 mg, 0.030 mmol), azide **11c** (8.8 mg, 0.030 mmol), $[Cu(MeCN)_4]PF_6$ (0.9 mg, 2.5 μmol) and N^iPr_2Et (4.8 μL) in THF (0.5 mL). Flash column chromatography (0-30% MeCN/1:1 hexane:CH_2Cl_2 (+0.25% EtOH)) afforded the target (20.1 mg, 80%) as a white foam: ^{1}H NMR (500 MHz, $CDCl_3$) δ ppm 8.30 (t, *J*=1.5, 1H, H_l), 7.75 (d, *J*=1.5, 2H, H_k), 7.62 (br. s, 1H, H_g) 7.23 (d, *J*=8.4, 6H, H_b), 7.10 (d, *J*=9.0, 2H, H_d), 7.07 (d, *J*=8.4, 6H, H_c), 6.84 (d, *J*=9.0, 2H, H_e), 5.17 (s, 2H, H_f), 4.61 (t, *J*=6.8, 2H, H_h), 4.09 (t, *J*=5.6, 2H, H_k), 3.92 (s, 6H, H_m), 2.46 (app. quin., *J*=6.3, 2H, H_i), 1.30 (s, 27H, H_a); ^{13}C NMR (125 MHz, $CDCl_3$) δ ppm 166.1 (C), 158.6 (C), 156.3 (C), 148.5 (C), 144.2 (C), 144.0 (C), 140.4 (C), 132.5 (CH), 132.1 (C), 130.9 (CH), 130.8 (CH), 124.2 (CH), 123.6 (CH), 119.9 (CH), 113.4 (CH), 64.8 (CH_2), 63.2 (C), 62.2 (CH_2) , 52.6 (CH_3), 47.2 (CH_2), 34.4 (C), 31.5 (CH_3), 29.9 (CH_2); HRMS (ESI+) m/z 836.4638 $[M+H]^+$ (calc. for $C_{53}H_{62}N_3O_6$ 836.4633).

Synthesis of Heterocircuit [3]Rotaxane 15c

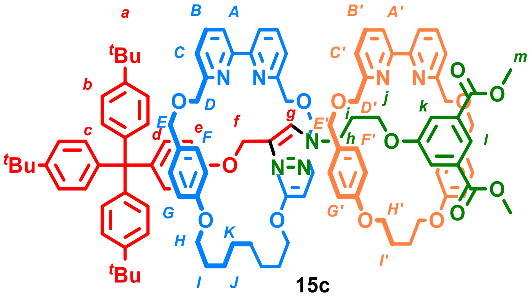

15c

Heterocircuit [3]Rotaxane **15c** was made according to the general heterocircuit rotaxanation conditions, using macrocycle **1** (5.4 mg, 0.010 mmol), macrocycle **2c** (4.8 mg, 0.010 mmol), azide **11c** (7.0 mg, 0.024 mmol), alkyne **4** (13.0 mg, 0.024 mmol) and $[Cu(MeCN)_4]PF_6$ (7.1 mg, 0.0096 mmol), in CH_2Cl_2 (1 mL). This afforded 19% conversion of macrocycle **1** to the target **15c** by ^{1}H NMR, of which an analytical sample was isolated by flash column chromatography (0-30% MeCN/1:1 hexane:CH_2Cl_2 (+0.25% EtOH)) as a white foam: ^{1}H NMR (500 MHz, $CDCl_3$) δ ppm 8.18 (t, *J*=1.5, 1H, H_l), 7.86 (s, 1H, H_g), 7.76 (d, *J*=7.7, 2H, H_A), 7.67 (t, *J*=7.9, 2H, $H_{B'}$), 7.55 (d, *J*=7.9, 2H, $H_{A'}$), 7.46 (d, *J*=7.9, 2H, $H_{C'}$), 7.40 (d, *J*=1.5, 2H, H_k), 7.36 (t, *J*=7.7, 2H, H_B), 7.25 - 7.30 (m, 8H, H_b and H_C), 7.03 (d, *J*=8.7, 4H, $H_{F'}$), 6.98 (d, *J*=8.7, 6H, H_c), 6.92 (d, *J*=8.7, 4H, H_F), 6.70 (d, *J*=8.7, 4H, $H_{G'}$), 6.44 (d, *J*=8.7, 4H, H_G), 6.30 (d, *J*=9.0, 2H, H_d), 5.40 (d, *J*=9.0, 2H, H_e), 4.42 - 4.67 (m, 12H, six of H_D, H_E, $H_{D'}$ or $H_{E'}$), 4.22 - 4.37 (m, 6H, one of $H_{H'}$ and two of H_D, H_E, $H_{D'}$ or $H_{E'}$), 4.20 (s, 2H, H_f), 3.99 - 4.07 (m, 2H, one of $H_{H'}$), 3.94 (s, 6H, H_m), 3.64 (dt, *J*=9.8, 6.1, 2H, one of H_H) 3.51 (dt, *J*=9.6, 6.8, 2H, one of H_H), 3.34 - 3.39 (m, 2H, H_h), 3.10 (t, *J*=6.3, 2H, H_j), 1.87 - 2.16 (m, 4H, $H_{I'}$), 1.33 – 1.48 (m, 4H, H_I), 1.35 (s, 27H, H_a), 0.92 - 1.06 (m, 6H, H_J and H_i), 0.81 - 0.91 (m, 4H, H_K); ^{13}C NMR (125 MHz, $CDCl_3$) δ ppm 166.4 (C), 159.3 (C), 158.9 (C), 158.8 (C), 158.7 (C), 158.5 (C), 156.6 (C), 155.7 (C), 155.3 (C), 148.1 (C), 144.6 (C), 142.9 (C), 138.5 (C), 137.4 (CH), 136.8 (CH), 131.3 (CH), 131.3 (C), 131.0 (CH), 130.6 (CH), 129.9 (CH), 129.6 (C), 129.3 (C), 124.3 (CH), 124.1 (CH), 122.6 (CH), 121.5 (CH), 121.1 (CH), 120.2 (CH), 120.1 (CH), 119.6 (CH), 115.2 (CH), 114.6 (CH), 112.4 (CH), 73.1 (CH_2), 72.4 (CH_2), 71.2 (CH_2), 71.1 (CH_2), 67.6 (CH_2), 66.8 (CH_2), 65.2 (CH_2), 63.0 (C), 61.3 (CH_2), 52.4 (CH_3), 46.3 (CH_2), 34.5 (C), 31.7 (CH_3), 29.2 (CH_2), 28.9 (CH_2), 28.9 (CH_2), 25.8 (CH_2), 24.9 (CH_2); LRMS (ESI+) m/z 1857.9 $[M+H]^+$.

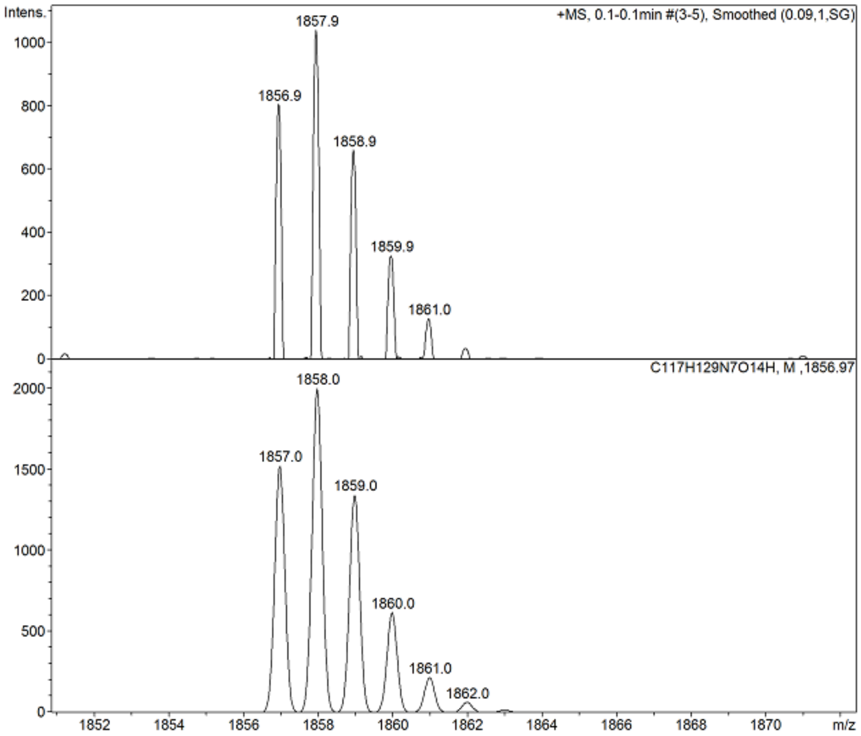

Dimethyl 5-(propargyloxy)isophthalate (S10)

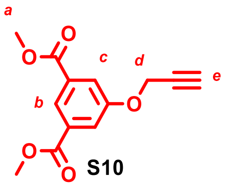

S10

To a solution of dimethyl 5-hydroxyisophthalate (**S9**, 2.10 g, 10 mmol) and K_2CO_3 (6.91 g, 50 mmol) in acetone (100 mL) was added propargyl bromide (80% wt./toluene, 1.67 mL, 2.23 g, 15 mmol). The solution was then stirred at reflux for 16 h, allowed to cool then diluted with H_2O (250 mL). The solution was then extracted with CH_2Cl_2 (250 mL, 100 mL), the organic extracts combined, dried ($MgSO_4$) and reduced *in vacuo* to afford the target material (2.38 g, 96%) as a white solid: m.p. 96-98 °C; ^1H NMR (500 MHz, $CDCl_3$) δ ppm 8.32 (t, *J*=1.5, 1H, H_b), 7.83 (d, *J*=1.5, 2H, H_c), 4.78 (d, *J*=2.5, 2H, H_d), 3.94 (s, 6H, H_a), 2.55 (t, *J*=2.5, 1H, H_e); ^{13}C NMR (125 MHz, $CDCl_3$) δ ppm 166.1 (C), 157.7 (C), 132.0 (C), 124.0 (CH), 120.5 (CH), 77.7 (CH), 76.4 (C), 56.4 (CH_2), 52.6 (CH_3); HRMS (ESI+) m/z 271.0582 $[M+Na]^+$ (calc. for $C_{13}H_{12}NaO_5$ 271.0577).

Attempted Synthesis of [3]Rotaxane S12

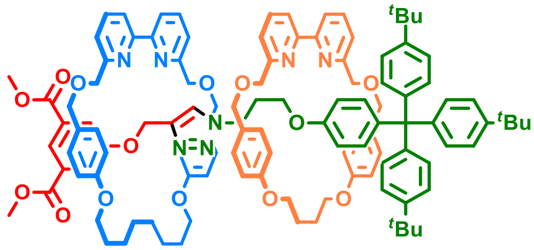

S12 - *not observed*

The synthesis of [3]Rotaxane **S12** was *attempted* according to the aforementioned heterocircuit rotaxanation conditions, using macrocycle **1** (5.4 mg, 0.010 mmol), macrocycle **2c** (5.4 mg, 0.010 mmol), azide **3** (14.1 mg, 0.024 mmol), alkyne **S10** (6.0 mg, 0.024 mmol) and $[Cu(MeCN)_4]PF_6$ (7.1 mg, 0.0192 mmol), in CH_2Cl_2 (1 mL).

After work-up, only [2]rotaxane **S11** (*vide infra*), triazole **S13** (*vide infra*) and macrocycles were observed.

Synthesis of [2]Rotaxane S11

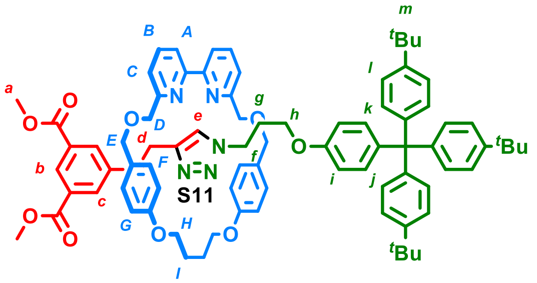

[2]Rotaxane **S11** was made according to the general [2]rotaxane synthesis conditions, using alkyne **S10** (7.4 mg, 0.030 mmol), azide **3** (17.6 mg, 0.030 mmol), macrocycle **2c** (11.3 mg, 0.025 mmol), $[Cu(MeCN)_4]PF_6$ (0.9 mg, 2.5 µmol) and N^iPr_2Et (4.8 µL) in THF (0.5 mL). Flash column chromatography (0-30% MeCN/1:1 hexane:CH_2Cl_2 (+0.25% EtOH)) afforded the target (33.3 mg, quant.) as a white foam: ^{1}H NMR (500 MHz, $CDCl_3$) δ ppm 8.34 (s, 1H, H_e), 8.20 (t, *J*=1.4, 1H, H_b), 7.69 (d, *J*=1.4, 2H, H_c), 7.63 (t, *J*=7.8, 2H, H_B), 7.48 (d, *J*=7.8, 2H, H_A), 7.40 (d, *J*=7.8, 2H, H_C), 7.24 (d, *J*=8.7, 6H, H_l), 7.11 (d, *J*=8.7, 6H, H_k), 7.00 (d, *J*=9.0, 2H, H_j), 6.89 (d, *J*=8.7, 4H, H_F), 6.65 (d, *J*=8.7, 4H, H_G), 6.48 (d, *J*=9.0, 2H, H_e), 4.95 (s, 2H, H_d), 4.37 - 4.58 (m, 4H, H_D or H_E), 4.30 - 4.37 (m, 2H, one of H_H), 4.06 - 4.17 (m, 6H, one of H_H and (H_D or H_E)), 3.99 - 4.09 (m, 2H, one of H_H), 3.91 (s, 6H, H_a), 3.33 - 3.39 (m, 2H, H_f), 3.27 (t, *J*=6.1, H_h), 1.91 - 2.13 (m, 4H, H_I), 1.31 (s, 27H, H_a), 1.15 - 1.23 (m, 2H, H_g); ^{13}C NMR (125 MHz, $CDCl_3$) δ ppm 166.2 (C), 159.2 (C), 158.9 (C), 158.6 (C), 156.5 (C), 156.2 (C), 148.4 (C), 144.4 (C), 141.7 (C), 139.4 (C), 137.3 (CH), 132.1 (CH), 131.7 (C), 130.9 (CH), 130.3 (CH), 128.9 (C), 125.2 (CH), 124.2 (CH), 123.2 (CH), 121.3 (CH), 121.0 (CH), 120.2 (CH), 115.2 (CH), 113.1 (CH), 73.0 (CH_2), 71.0 (CH_2), 66.6 (CH_2), 64.5 (CH_2), 63.2 (C), 62.3 (CH_2), 52.5 (CH_3), 46.6 (CH_2), 34.4 (C), 31.5 (CH_3), 28.4 (CH_2), 24.8 (CH_2); LRMS (ESI+) m/z 1318.7 $[M+H]^+$.

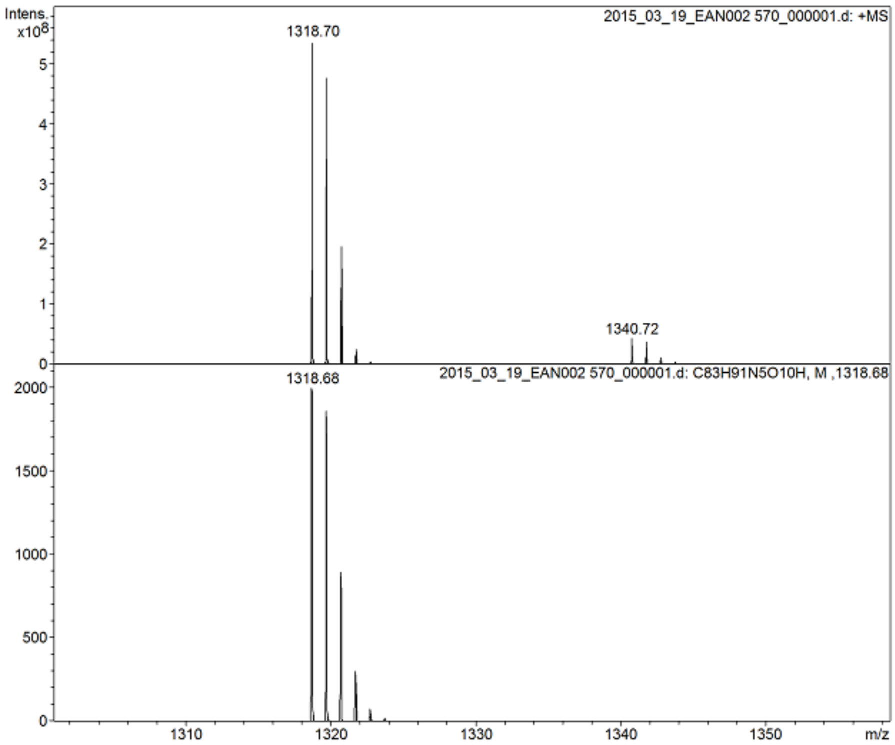

Synthesis of Thread Triazole S13

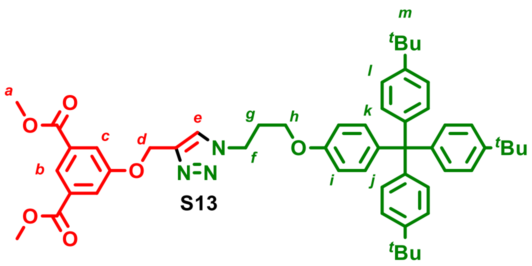

S13

Thread triazole **S13** was made according to the general thread triazolide synthesis conditions, using alkyne **S10** (7.4 mg, 0.030 mmol), azide **3** (17.6 mg, 0.030 mmol), $[Cu(MeCN)_4]PF_6$ (0.9 mg, 2.5 μmol) and N^iPr_2Et (4.8 μL) in THF (0.5 mL). Flash column chromatography (0-30% MeCN/1:1 hexane:CH_2Cl_2 (+0.25% EtOH)) afforded the target (16.3 mg, 65%) as a white foam: ^{1}H NMR (400 MHz, $CDCl_3$) δ ppm 8.31 (t, *J*=1.5, 1H, H_b), 7.84 (t, *J*=1.5, 2H, H_c), 7.66 (s, 1H, H_e), 7.23 (d, *J*=8.7, 6H, H_l), 7.03 - 7.13 (m, 8H, H_j and H_k), 6.75 (d, *J*=8.9, 2H, H_i), 5.28 (s, 2H, H_d), 4.60 (t, *J*=6.9, 2H, H_f), 3.97 (t, *J*=5.6, 2H, H_h), 3.92 (s, 6H, H_a), 2.41 (app. quin., *J*=6.3, 2H, H_g), 1.30 (s, 27H, H_m); ^{13}C NMR (100 MHz, $CDCl_3$) δ ppm 166.1 (C), 158.5 (C), 156.4 (C), 148.5 (C), 144.2 (C), 143.4 (C), 140.4 (C), 132.5 (CH), 132.1 (C), 130.9 (CH), 124.2 (CH), 123.7 (CH), 123.4 (CH), 120.3 (CH), 113.1 (CH), 64.0 (CH_2), 63.2 (C), 62.6 (CH_2), 52.6 (CH_3), 47.5 (CH_2), 34.4 (C), 31.5 (CH_3), 30.2 (CH_2); HRMS (ESI+) m/z 836.4631 $[M+H]^+$ (calc. for $C_{53}H_{62}N_3O_6$ 836.4633).

4. Development of the Kinetic Self-Sorting Reaction

4.1 Small Azide Screen for Kinetic Self-Sorting

In order to be suitable for the Kinetic Self-Sorting protocol, the azide stopper has to be both passable by large macrocycle **1** and impassable to small macrocycle **2c**. Three candidates were chosen as shown in **Figure S1**.

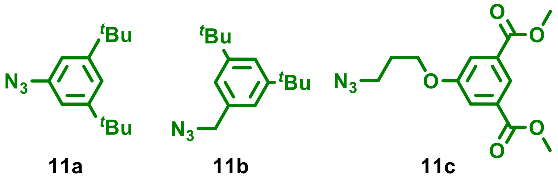

Figure S1. Azide stoppers screened for the Kinetic Self-Sorting protocol.

To test their applicability for the active template-copper-mediated azide-alkyne cycloaddition (AT-CuAAC) reaction, all three azides were reacted with large alkyne **4** and $[Cu(MeCN)_4]PF_6$ to form triazole threads (**Scheme S1**). After 30 minutes, thread formation was detected by ^{1}H NMR and the triazoles isolated and characterised. Repeating these experiments in the presence of small macrocycle **2c** duly afforded quantitative conversion to [2]rotaxanes **S3-S5** by ^{1}H NMR.

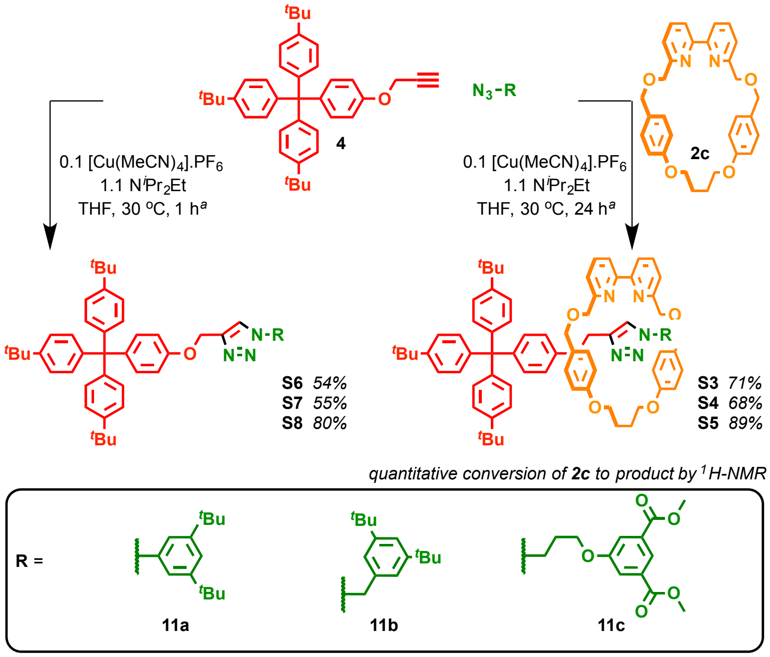

Scheme S1. Test of small azide candidate stoppers in CuAAC and AT-CuAAC reactions.

Satisfied that the azides were suitable for the AT-CuAAC reaction and capable of retaining the small macrocycle **2c**, test reactions were undertaken with each azide in the presence of large macrocycle **1** to attempt to form the heterocircuit [3]rotaxane (**Scheme S2**). When azides **11a** or **11b** were used, ^{1}H NMR analysis of the crude reaction mixture after workup with NH_3-EDTA indicated consumption of **1** and formation of metastable [2]pseudorotaxanes **12a** and **12b** respectively. [3]rotaxane **15a** was not observed in the case of **11a** whereas only a trace amounts (< 2.5%) of **15b** was observed in the case of **11b**.

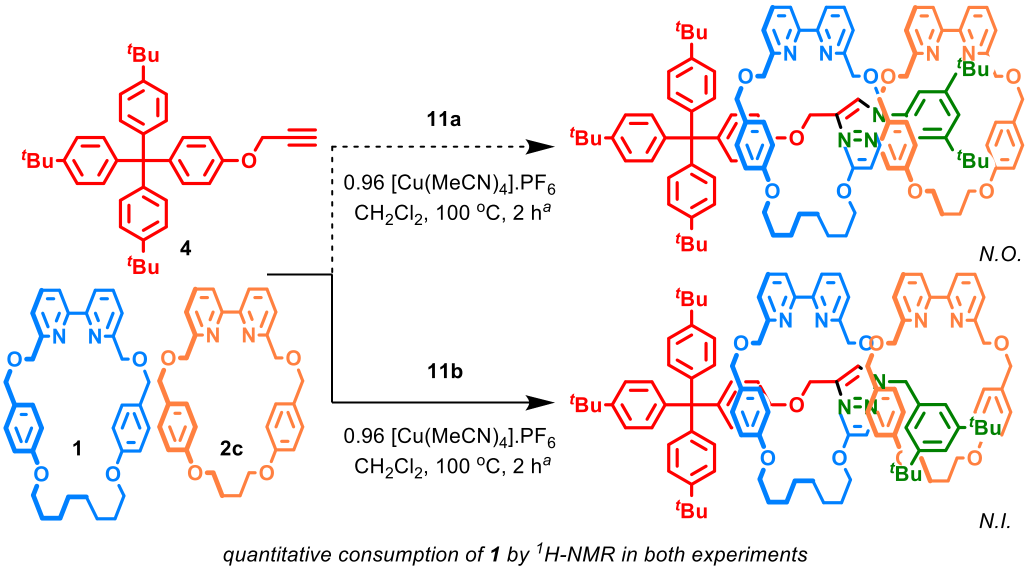

Scheme S2. Attempted synthesis of heterocircuit [3]rotaxanes from small azides **11a** and **11b**. N.O. = not observed. N.I. = not isolated (<2.5% conversion to **15b** by ^{1}H NMR).

The test reaction with azide **11c**, by contrast, afforded 19% conversion to heterocircuit [3]rotaxane alongside recovered macrocycle **1**, thread S8 and [2]rotaxane S5 (**Scheme S3**). No metastable [2]pseudorotaxane **12c** was observed. Heterocircuit [3]rotaxane **15c** was isolated by column chromatography.

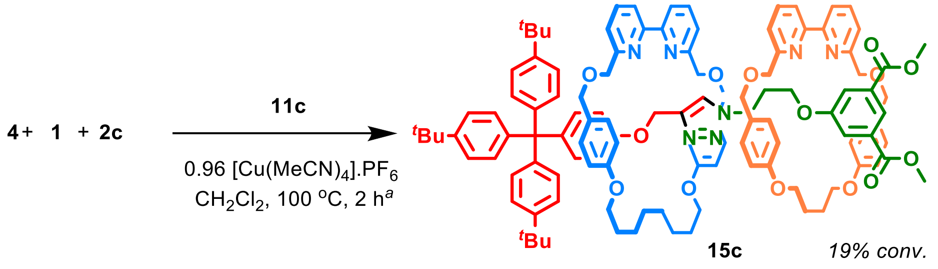

Scheme S3. Synthesis of heterocircuit [3]rotaxane **15c** from small azide **11c**.

As a result of these tests, ester azide **11c** was taken forward: small macrocycle **2c** is retained and large macrocycle **1** may pass, with no unexpected metastable products exist as with the other systems tested.

4.2 Analogous Reaction with Small Acetylene S10

To further validate the stereoselectivity of the reaction we repeated the self-sorting reaction using small acetylene **S10** and azide **3** (**Scheme S4**). To test the suitability of alkyne **S10** for the CuAAC reaction and AT-CuAAC reaction, it was reacted with large azide **3** with $[Cu(MeCN)_4]PF_6$ as a catalyst, either in the presence of absence of small macrocycle **2c**. Both [2]rotaxane **S11** and thread **S13** were made and isolated. The reaction of **S10** and **3** in the presence of **1** and **2c** only afforded thread **S13**, [2]rotaxane **S13** and recovered macrocycle **1** in keeping with our previous observations: the larger macrocycle, always held over the alkyne as per the proposed mechanism, is on the same side as the smaller steric barrier rendering any [3]rotaxane formed unstable to dethreading.

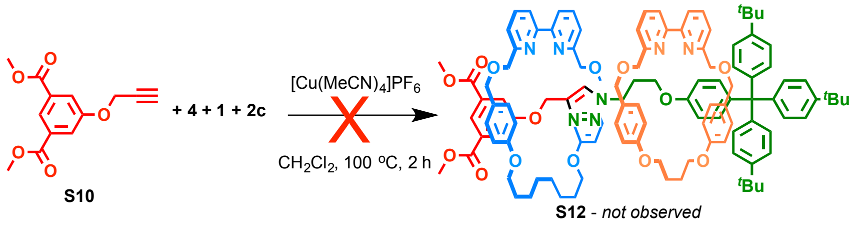

Scheme S4. Attempted synthesis of heterocircuit [3]rotaxane S12

4.3 Optimisation of alkyne and azide equivalents in the synthesis of 15c

We examined the optimal number of equivalents of **11c** and **4** to afford complete consumption of **2c** and maximise the yield of **15c**. The results suggest that the formation of heterocircuit [3]rotaxane **15** continues until smaller macrocycle **2c** is entirely consumed and this is achieved with 1 equivalent each (*vs* total macrocycle present) of the azide and alkyne component. The only practical benefit of this optimisation is to reduce the amount of thread **S8** and [2]rotaxane **12** by-products in the final reaction mixture. This ratio of components was used in the iterative self-sorting reaction.

Table S1. Optimisation of Feeds for the Kinetic Self-Sorting Protocol

Entry	Half-threads (eq.)	Consumption of **2c** (%)[a]	Conversion of **1** to **15c** (%)[a]
1	0.6	66	15
2	0.8	95	17
3	1.0	100	20
4	1.2	100	19

[a]Determined by ^{1}H NMR analysis of the crude reaction mixture.

4.4 Iterative Synthesis of Heterocircuit [3]Rotaxane 15c

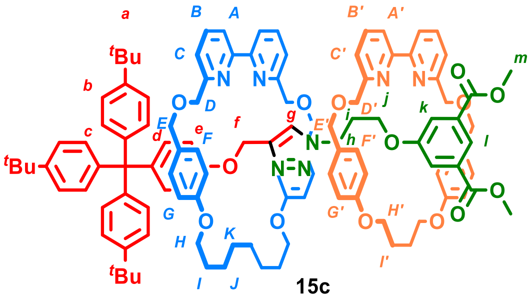

A solution of macrocycle **1** (10.8 mg, 0.020 mmol), macrocycle **2c** (9.6 mg, 0.020 mmol), azide **11c** (11.8 mg, 0.040 mmol), alkyne **4** (21.8 mg, 0.040 mmol) and $[Cu(MeCN)_4]PF_6$ (14.2 mg, 0.038 mmol) in CH_2Cl_2 (2 mL) was stirred at 100 °C in a 150W microwave reactor for 2 hours. The reaction mixture was then heated at 80 °C in an oil bath for 16 h, allowed to cool, aliquoted by microsyringe for analysis (50 µL; see note below) then solvent removed from the reaction vial *in vacuo*. A second portion of macrocycle **2c** (9.6 mg, 0.020 mmol), azide **11c** (11.8 mg, 0.040 mmol), alkyne **4** (21.8 mg, 0.040 mmol) and $[Cu(MeCN)_4]PF_6$ (7.1 mg, 0.019 mmol) in CH_2Cl_2 (2 mL) was added (see note below), the vial recapped and the solution stirred for a further 2 hours at 100 °C in a 150 W microwave reactor and then heated to 80 °C in an oil bath for 16 h followed by aliquotting and evaporation as before. This protocol of reaction, heating, aliquotting, evaporation and feeding was repeated for three further cycles. The final reaction solution was allowed to return to room temperature before dilution with further CH_2Cl_2 (50 mL) and washing with NH_3-EDTA (50 mL). The organic layer was retained and the aqueous layer extracted with CH_2Cl_2 (2 × 50 mL). The organic extracts were combined, dried over $MgSO_4$ and dried *in vacuo*. Column chromatography (0-30% MeCN/ 1:1 Hexane:CH_2Cl_2; +0.25% EtOH throughout) afforded the product as a white foam (13.9 mg, 42% taking into account aliquots removed), with characteristic data consistent with that stated above.

Notes

Aliquots for analysis were diluted with CH_2Cl_2 (1mL) then washed with NH_3-EDTA (1 mL). The organic layer was passed through $MgSO_4$, reduced *in vacuo* and analysed by ^{1}H NMR to determine the ratio between products using the characteristic signals of macrocycle **1** and [3]rotaxane **15c** at 7.94 ppm and 6.30 ppm respectively (see Fig 2 of manuscript).

Solutions of macrocycle 2c, azide 11c, alkyne 4 and were prepared as follows: macrocycle **2c** (10.4 mg, 0.024 mmol), azide **11c** (14.1 mg, 0.048 mmol), alkyne **4** (26.1 mg, 0.048 mmol) and $[Cu(MeCN)_4]PF_6$ (8.5 mg, 0.023 mmol) were dissolved in CH_2Cl_2 (2.4 mL) and stirred under an inert atmosphere for 10 minutes. A 2.0 mL portion of this solution was added to the residue of the previous iteration.

4.5 "All-in-One" Experiment for Comparison

A solution of macrocycle **1** (5.4 mg, 0.010 mmol), macrocycle **2c** (24.1 mg, 0.050 mmol), azide **11c** (35.2 mg, 0.120 mmol), alkyne **4** (65.1 mg, 0.120 mmol) and $[Cu(MeCN)_4]PF_6$ (21.5 mg, 0.0576 mmol) in CH_2Cl_2 (1 mL) was stirred at 100 °C in a 150W microwave reactor for 2 hours. The solution was allowed to return to room temperature before dilution with further CH_2Cl_2 (50 mL) and washing with NH_3-EDTA (50 mL). The organic layer was retained and the aqueous layer extracted with CH_2Cl_2 (2 × 50 mL). The organic extracts were combined, dried over $MgSO_4$ and dried *in vacuo*. ^{1}H NMR analysis of the crude reaction mixture revealed 28% conversion of macrocycle **1** of the target [3]rotaxane **15c**, based on their characteristic signals at 7.94 ppm and 6.30 ppm respectively (*vide supra*). Column chromatography (0-30% MeCN/ 1:1 Hexane:CH_2Cl_2; +0.25% EtOH throughout) afforded the product as a white foam (5.3 mg, 28%), with characteristic data consistent with that stated above.

4.6 Study of the stability of metastable [2]pseudorotaxane 12a

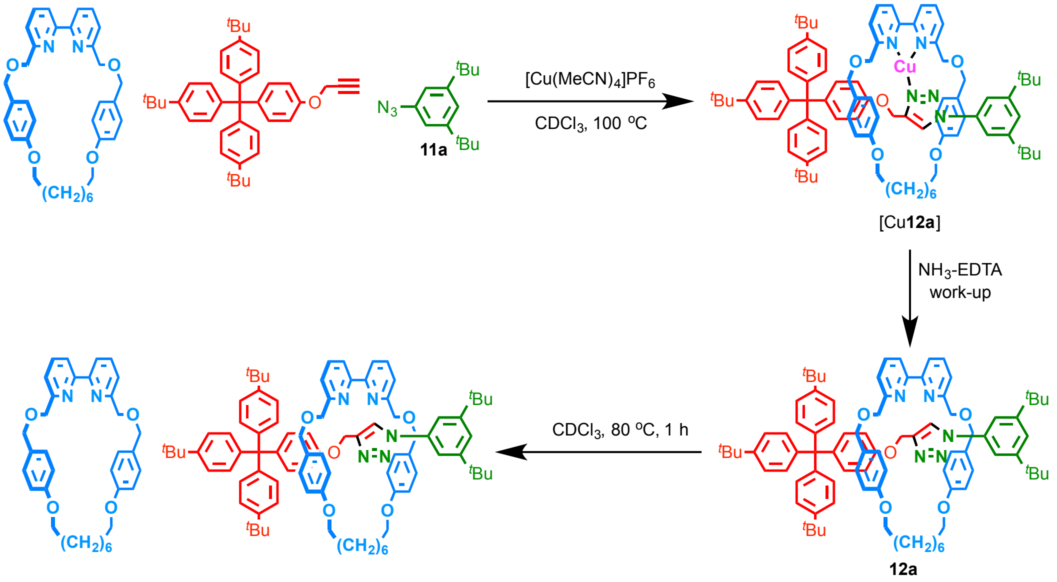

Scheme S5 – Formation of [Cu**12a**] and metastable [2]psuedorotaxane **12a**. 1.0 equiv. each **1**, 1.2 equiv. **4** and **12a**, 0.96 equiv. $[Cu(MeCN)_4]PF_6$ in $CDCl_3$.

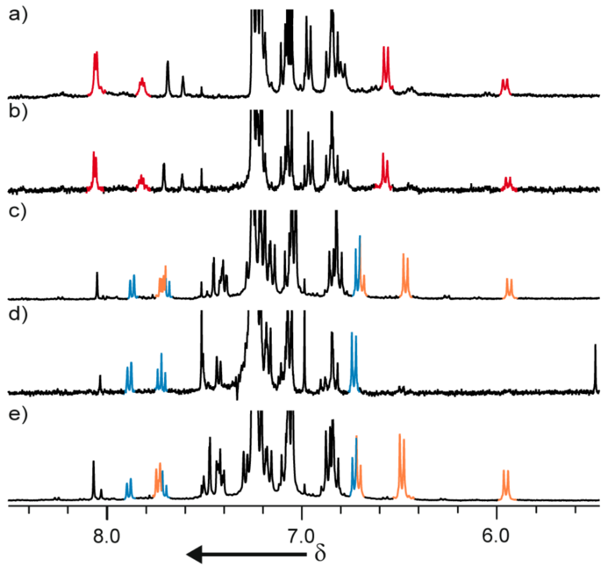

Figure S2 – Partial ^{1}H NMR (400 MHz, $CDCl_3$, 300 K) of the reaction mixture derived from **1**, **4** and **12a** a) immediately after the reaction reached completion *prior* to work-up; b) Sample *a* after 1 h further heating without work-up; c) sample a after work-up with NH_3-EDTA; d) sample *c* after 1 h further heating at 80 °C; e) Sample *a* after 18 hours heating and work-up with NH_3-EDTA. Principal signals identified of key compounds indicated as follows: [Cu**12a**] in red; macrocycle **1** in blue and [2]pseudorotaxane **12c** in orange.

Analysis of the reaction mixture prior to work-up (**Fig S2a**) indicated complete consumption of macrocycle **1** and the presence of a new interlocked species (signals shown in red). Analysis by MS revealed a mass ion corresponding to $[\mathbf{12a}+Cu+H]^{2+}$ suggesting that Cu remained coordinated. After aqueous work-up of a portion of the reaction mixture using NH_3-EDTA a new threaded species was observed (signals shown in orange), which was assigned as metastable [2]pseudorotaxane **12a** by analogy with previously isolated [2]rotaxanes, alongside some recovered **1** (blue). When this mixture was heated at 80 °C for 1 h the majority of the threaded species was destroyed, producing non-interlocked thread and macrocycle **1**. This demonstrated the instability of **12a** with respect to dethreading. In contrast, when the reaction mixture heated for a further hour at 80 °C no significant changes were observed; the species assigned as [Cu**12a**] persists and macrocycle **1** (or its copper complex [Cu**1**]) remains absent. Furthermore, even after heating the reaction mixture for 16 h at 80 °C, aqueous work-up with NH_3-EDTA confirmed the presence of **12a**. Taken together, these results not only confirm the presence of a metastable threaded product both before an after work-up, they strongly suggest that Cu stabilises the threaded species.

These results shed some light on the diminished efficiency of the self-sorting process in later rounds of AT-CuAAC; although in the first step 19% of macrocycle **1** is consumed, in later iterations the reaction appears to consume 12-8% of the remaining macrocycle. It seems likely that, although [2]pseudorotaxane **12c** and [3]pseudorotaxane **13c** dethread rapidly in the absence, Cu coordination stabilises the threaded species prior to work up and thus reduces the amount of free **1** available in the reaction mixture.

5. ROESY NMR Analysis of Heterocircuit [3]Rotaxanes

ROESY NMR of Heterocircuit [3]Rotaxane 9a

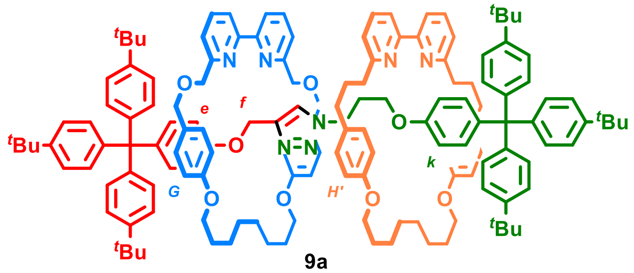

9a

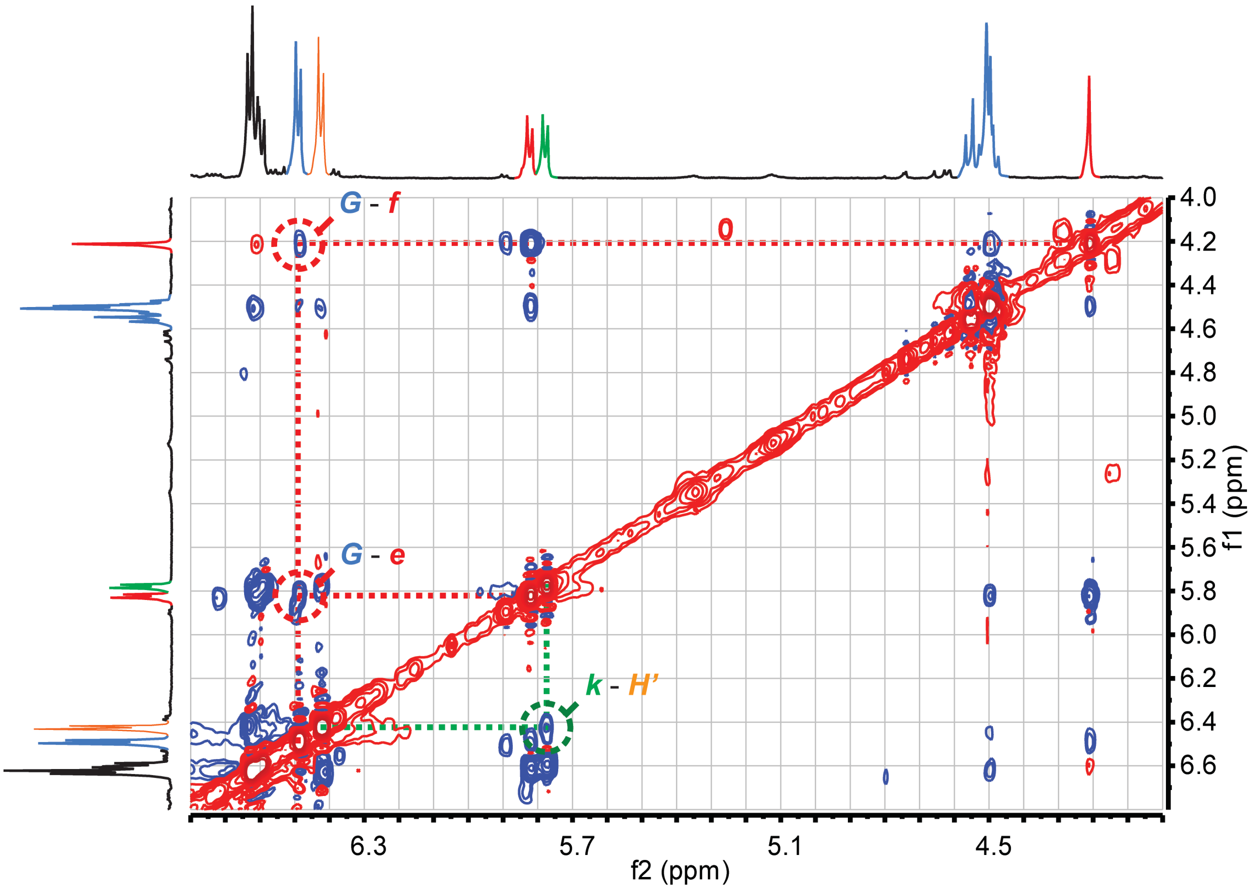

Figure S3 – Highlight of ROESY NMR of Heterocircuit [3]Rotaxane 9a (Full in Spectrum in Characteristic Data)

ROESY NMR of Heterocircuit [3]Rotaxane 9b

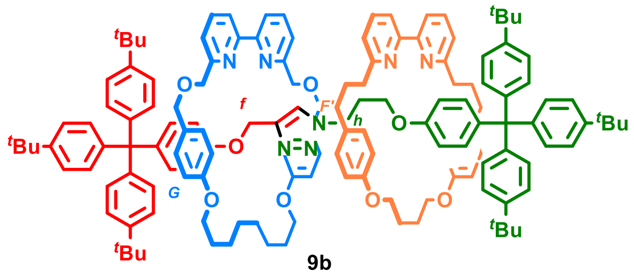

9b

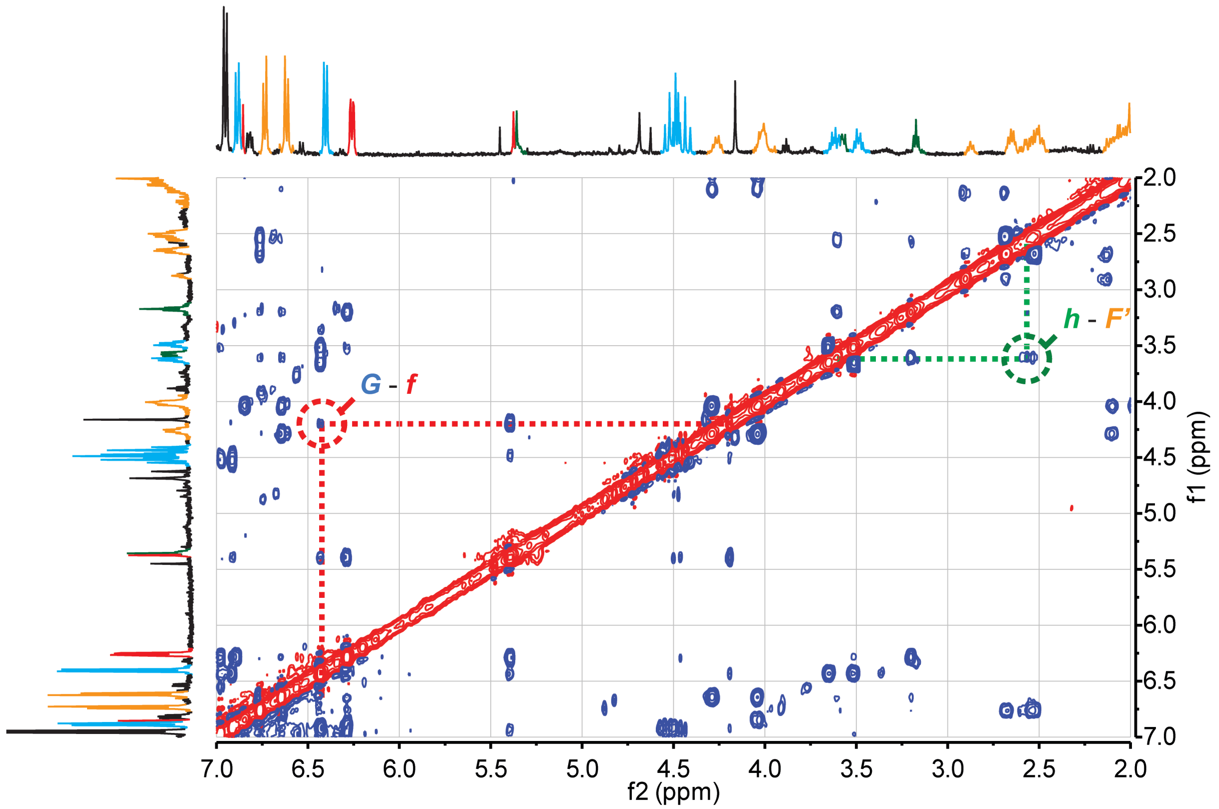

Figure S4 – Highlight of ROESY NMR of Heterocircuit [3]Rotaxane 9b (Full Spectrum in Characteristic Data)

ROESY NMR of Heterocircuit [3]Rotaxane 9c

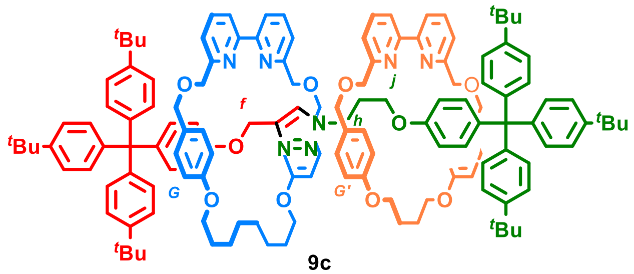

9c

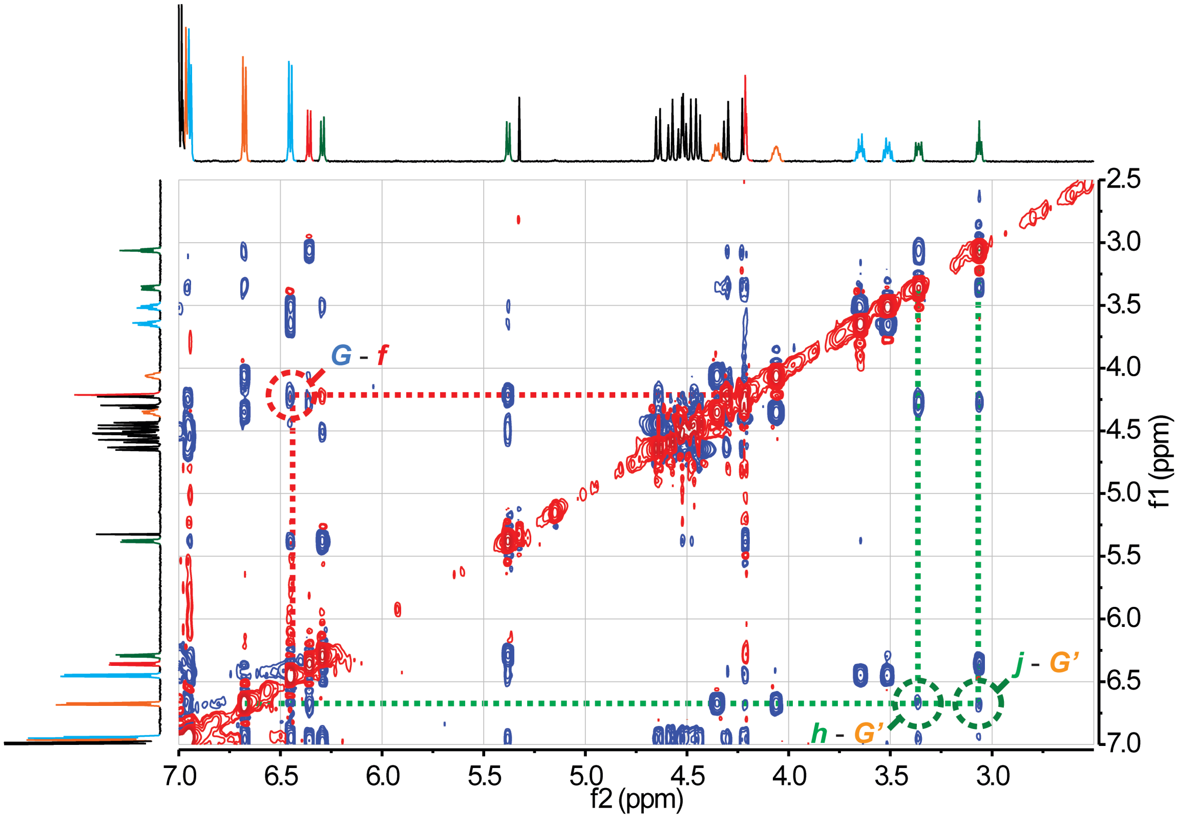

Figure S5 – Highlight of ROESY NMR of Heterocircuit [3]Rotaxane 9c (Full Spectrum in Characteristic Data)

ROESY NMR of Heterocircuit [3]Rotaxane 15c

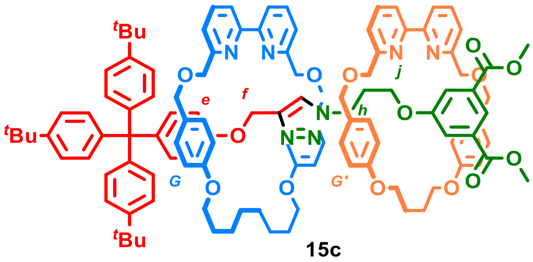

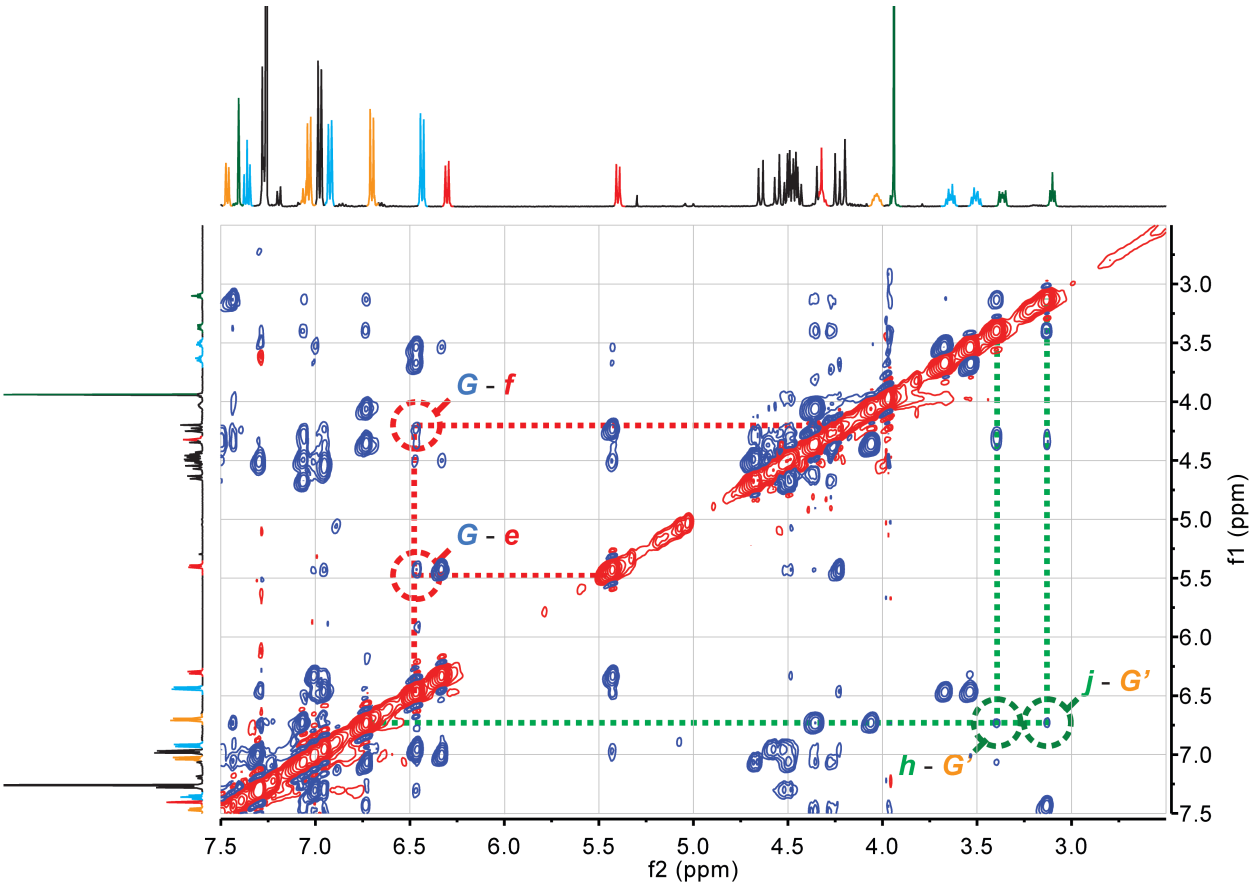

Figure S6 – Highlight of ROESY NMR of Heterocircuit [3]Rotaxane 15c (Full in Characteristic Data)

6. Characterisation Data

[3]Rotaxane 9a: ^{1}H NMR ($CDCl_3$, 600 MHz, 300 K)

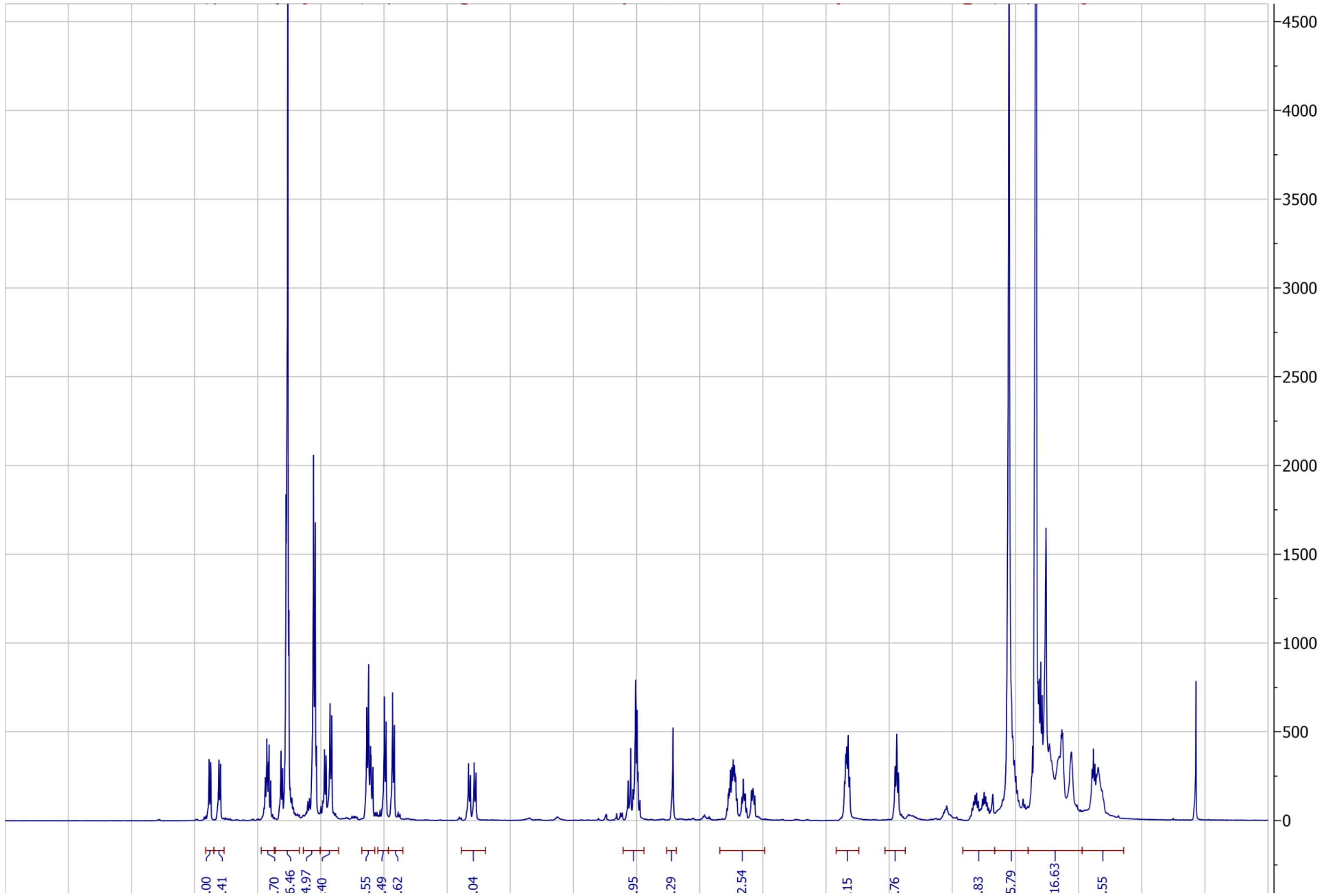

[3]Rotaxane 9a: ^{13}C NMR ($CDCl_3$, 150 MHz, 300 K)

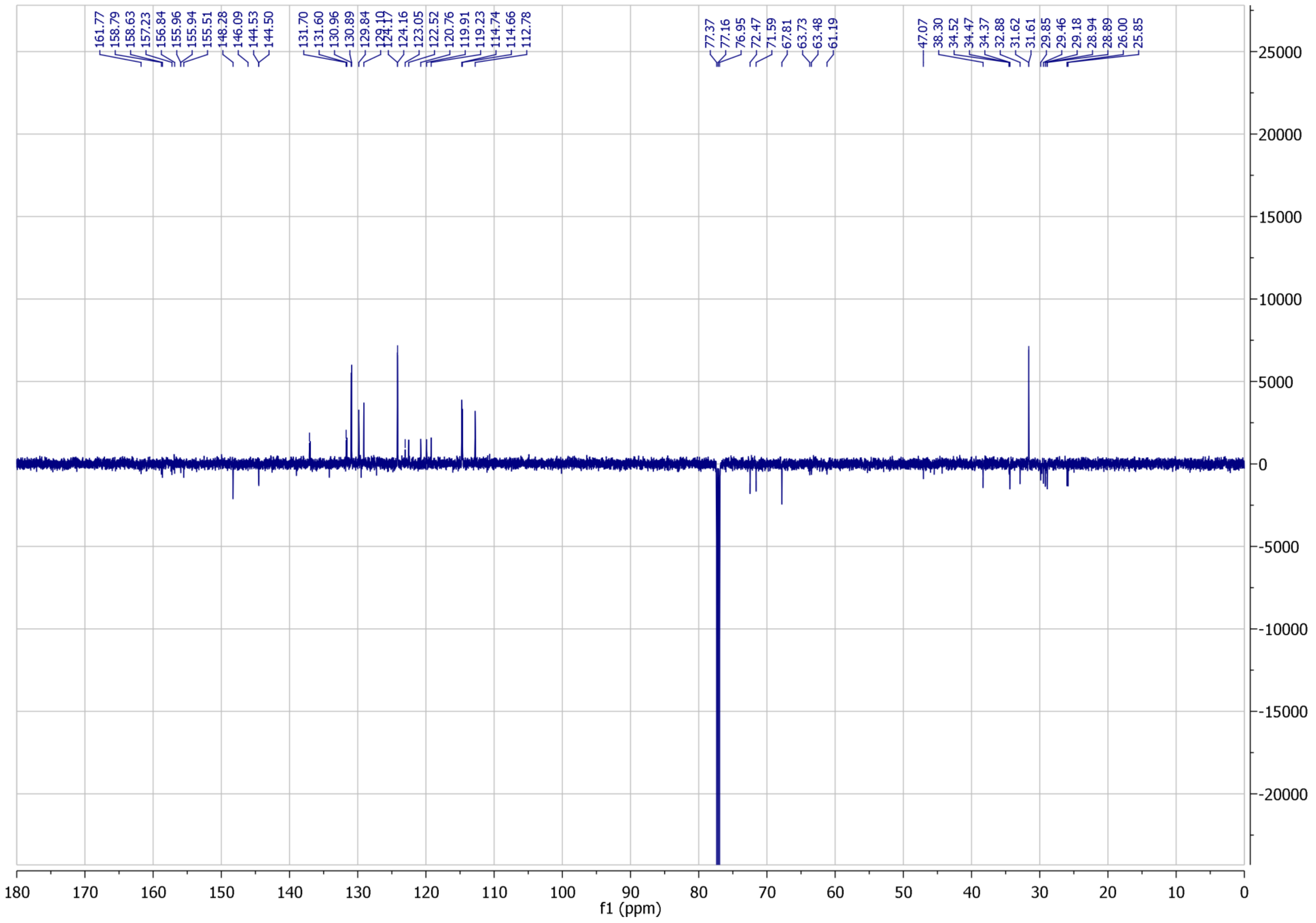

[3]Rotaxane 9a: COSY NMR ($CDCl_3$, 600 MHz, 300 K)

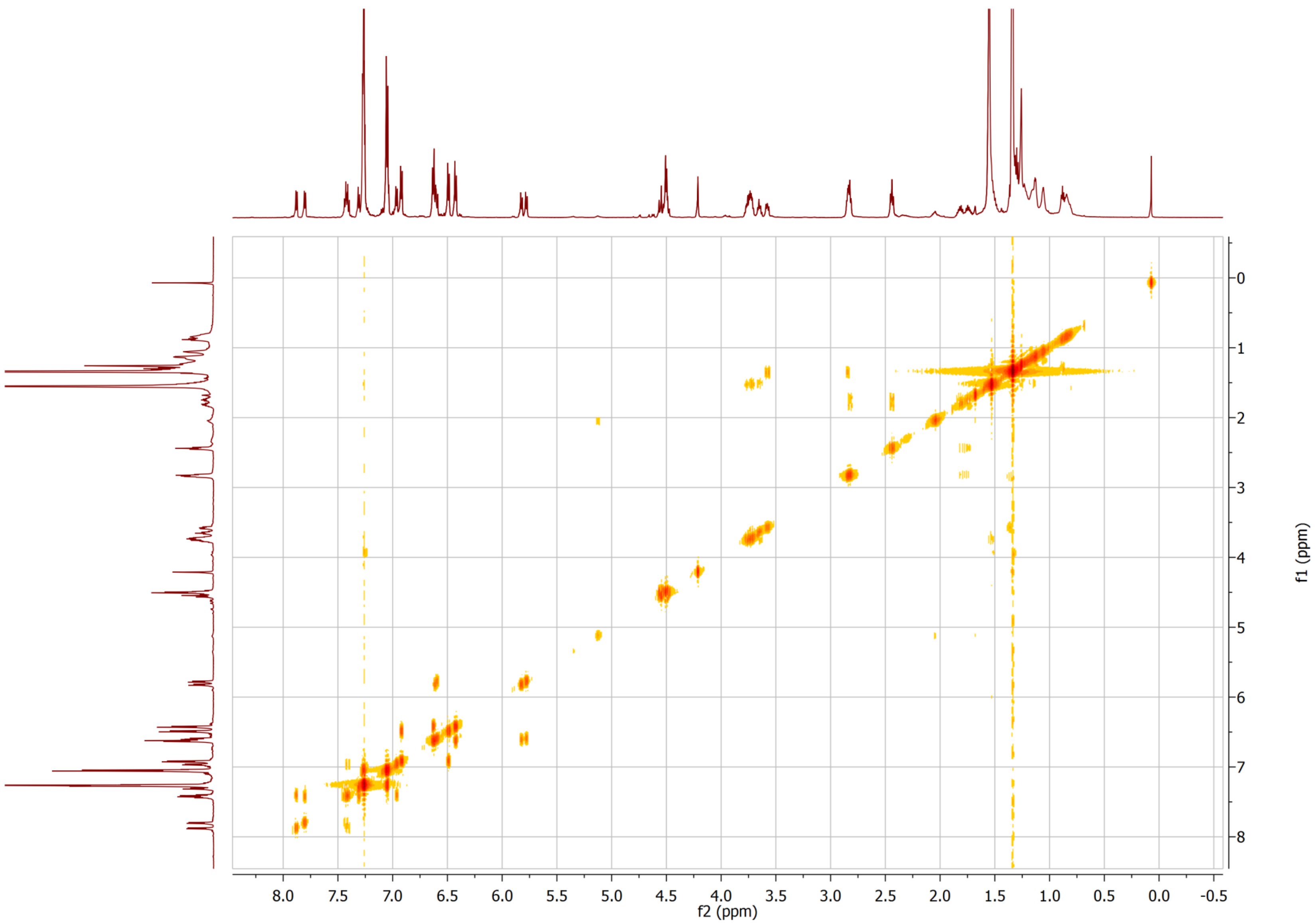

[3]Rotaxane 9a: HSQC NMR ($CDCl_3$, 600 MHz, 300 K)

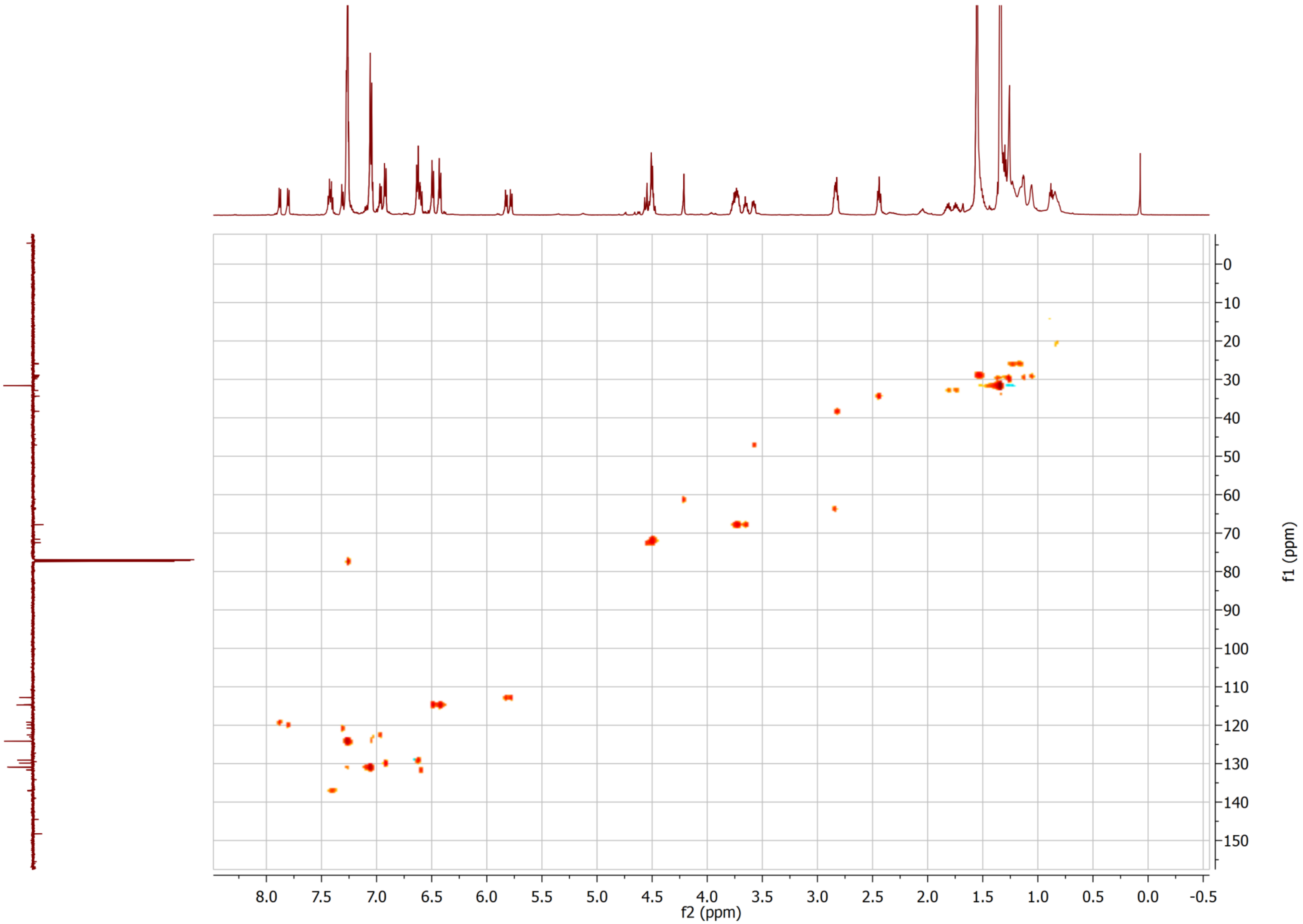

[3]Rotaxane 9a: HMBC NMR ($CDCl_3$, 600 MHz, 300 K)

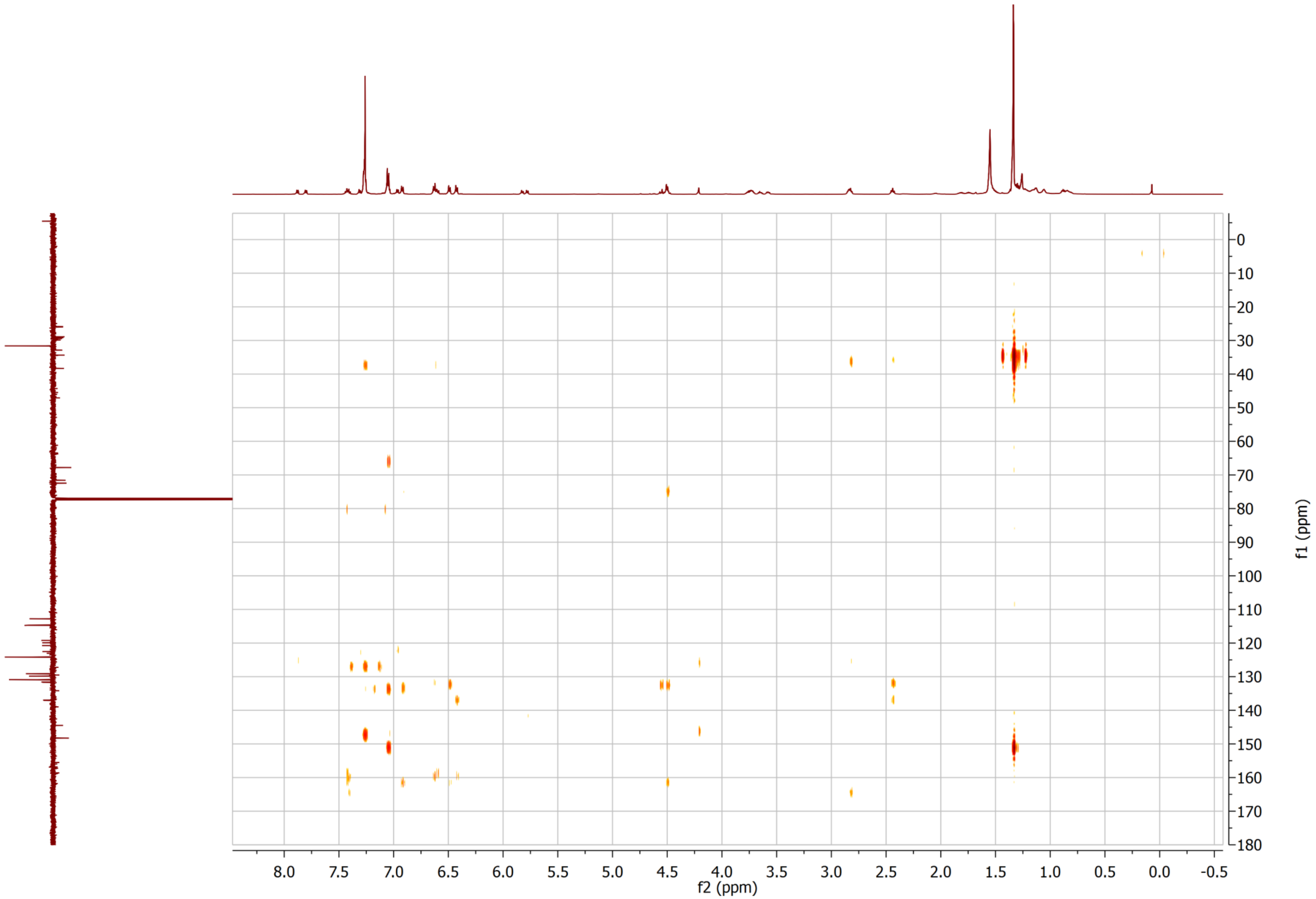

[3]Rotaxane 9a: ROESY NMR ($CDCl_3$, 600 MHz, 300 K)

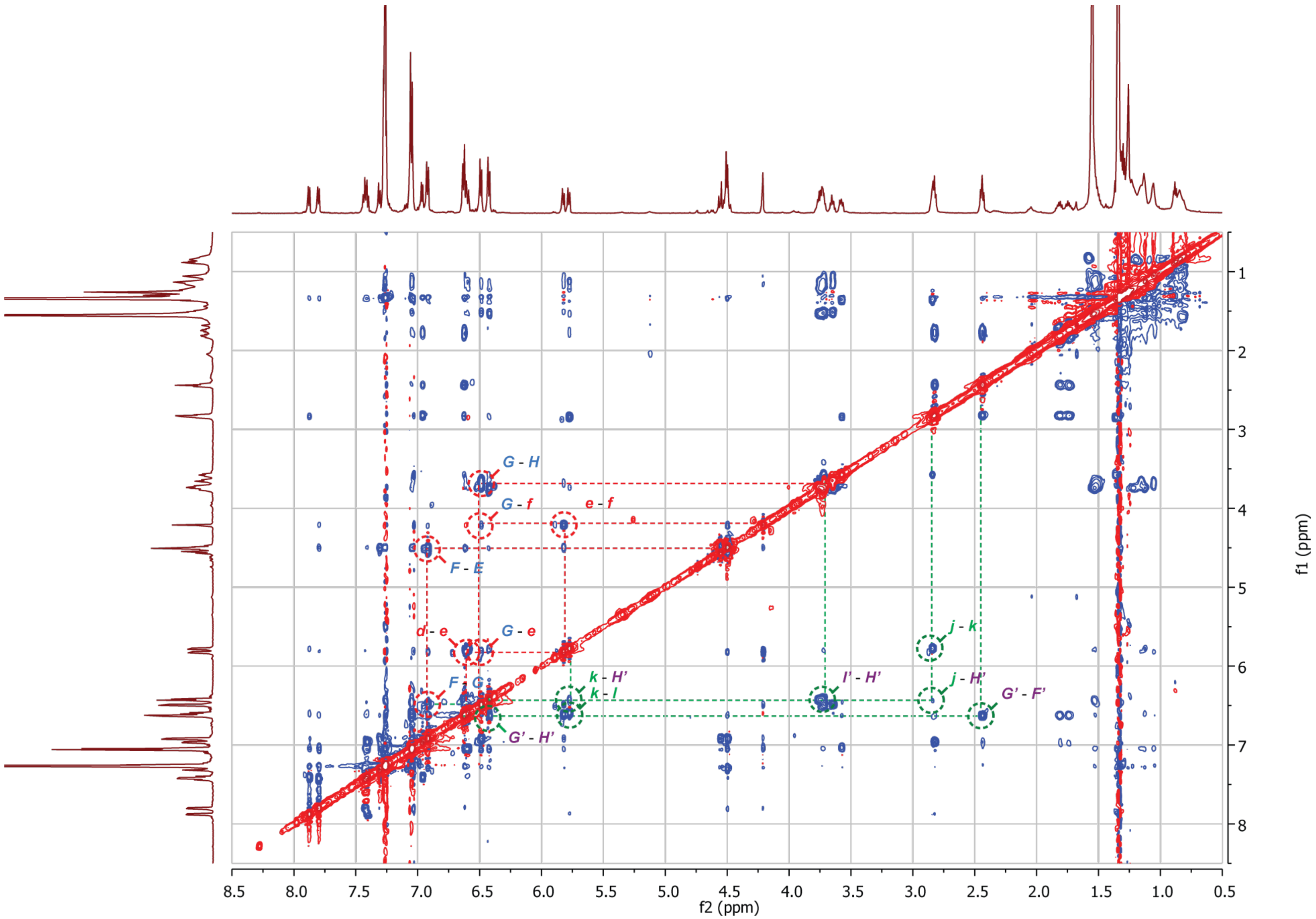

[3]Rotaxane 9b: ^{1}H NMR ($CDCl_3$, 500 MHz, 300 K)

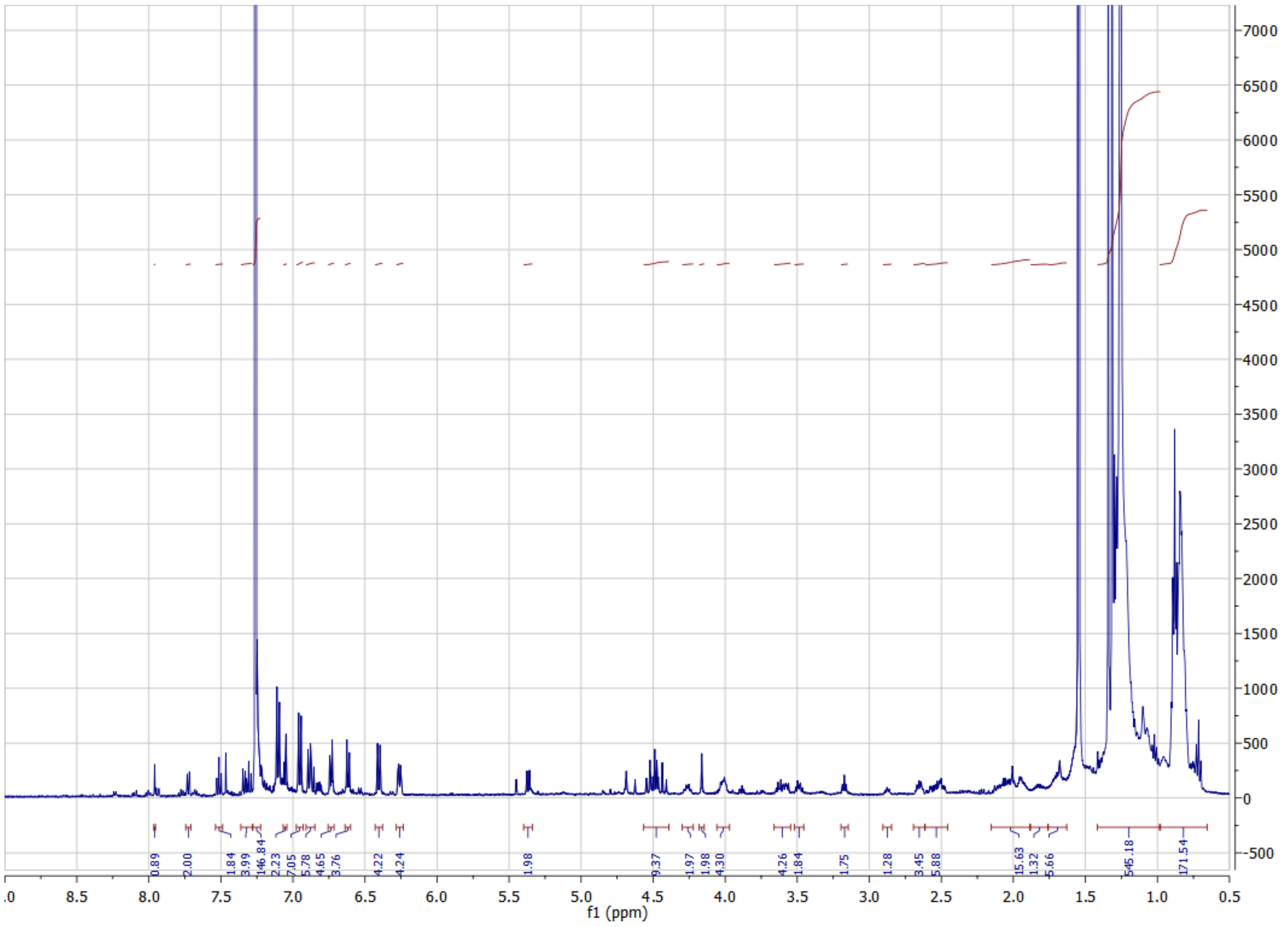

[3]Rotaxane 9b: ^{13}C NMR ($CDCl_3$, 125 MHz, 300 K)

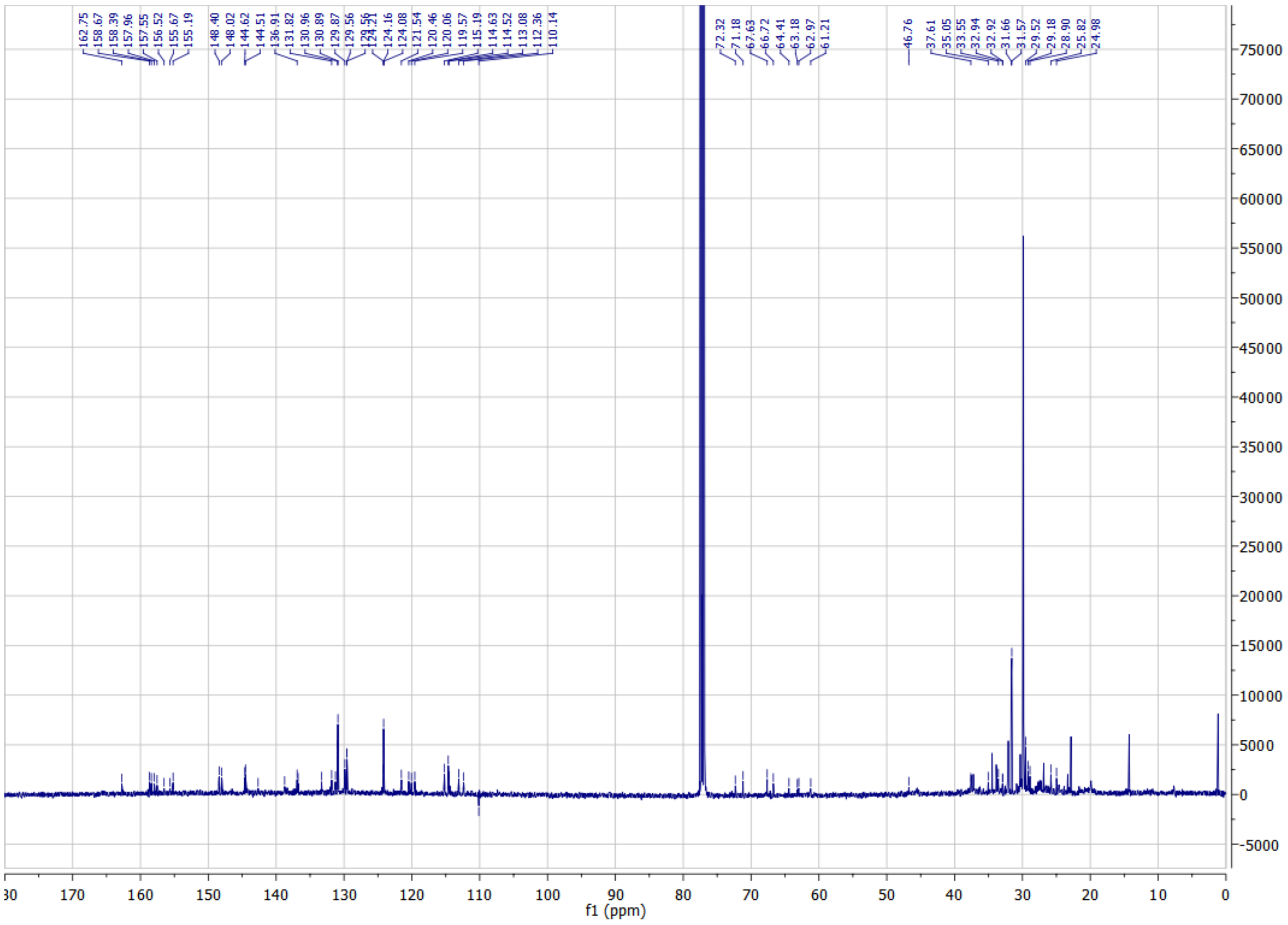

[3]Rotaxane 9b: COSY NMR ($CDCl_3$, 500 MHz, 300 K)

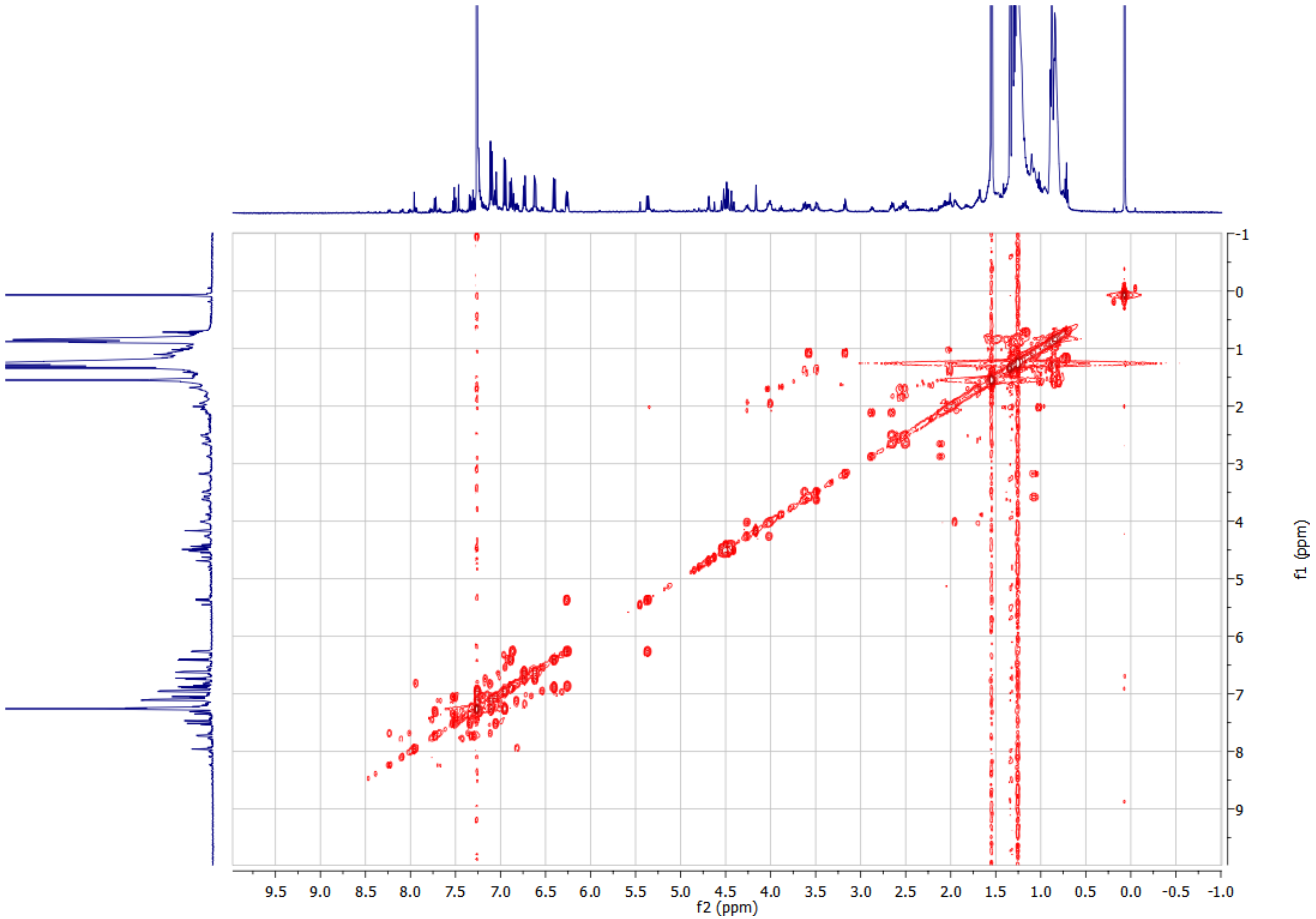

[3]Rotaxane 9b: HSQC NMR ($CDCl_3$, 500 MHz, 300 K)

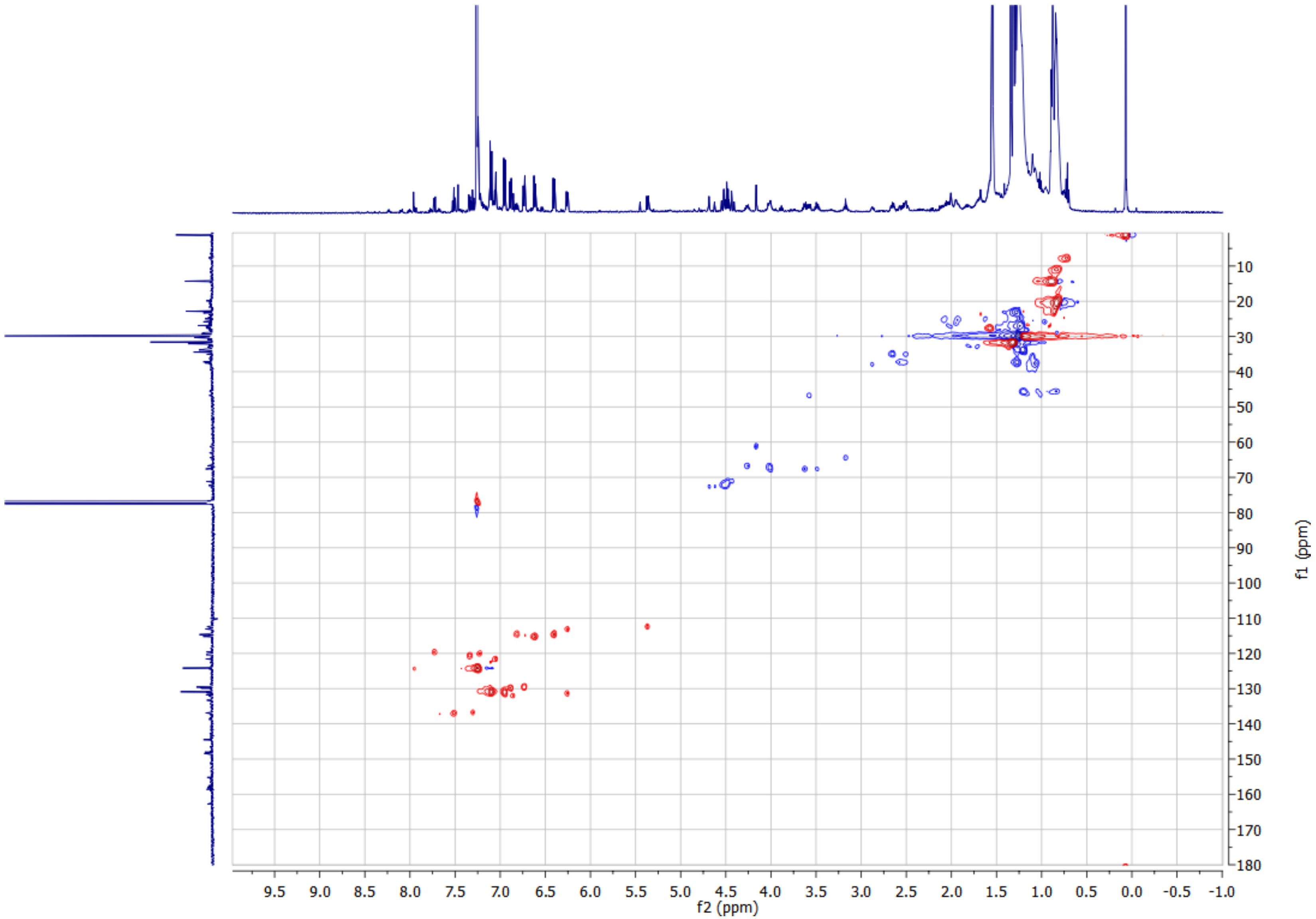

[3]Rotaxane 9b: HMBC NMR ($CDCl_3$, 500 MHz, 300 K)

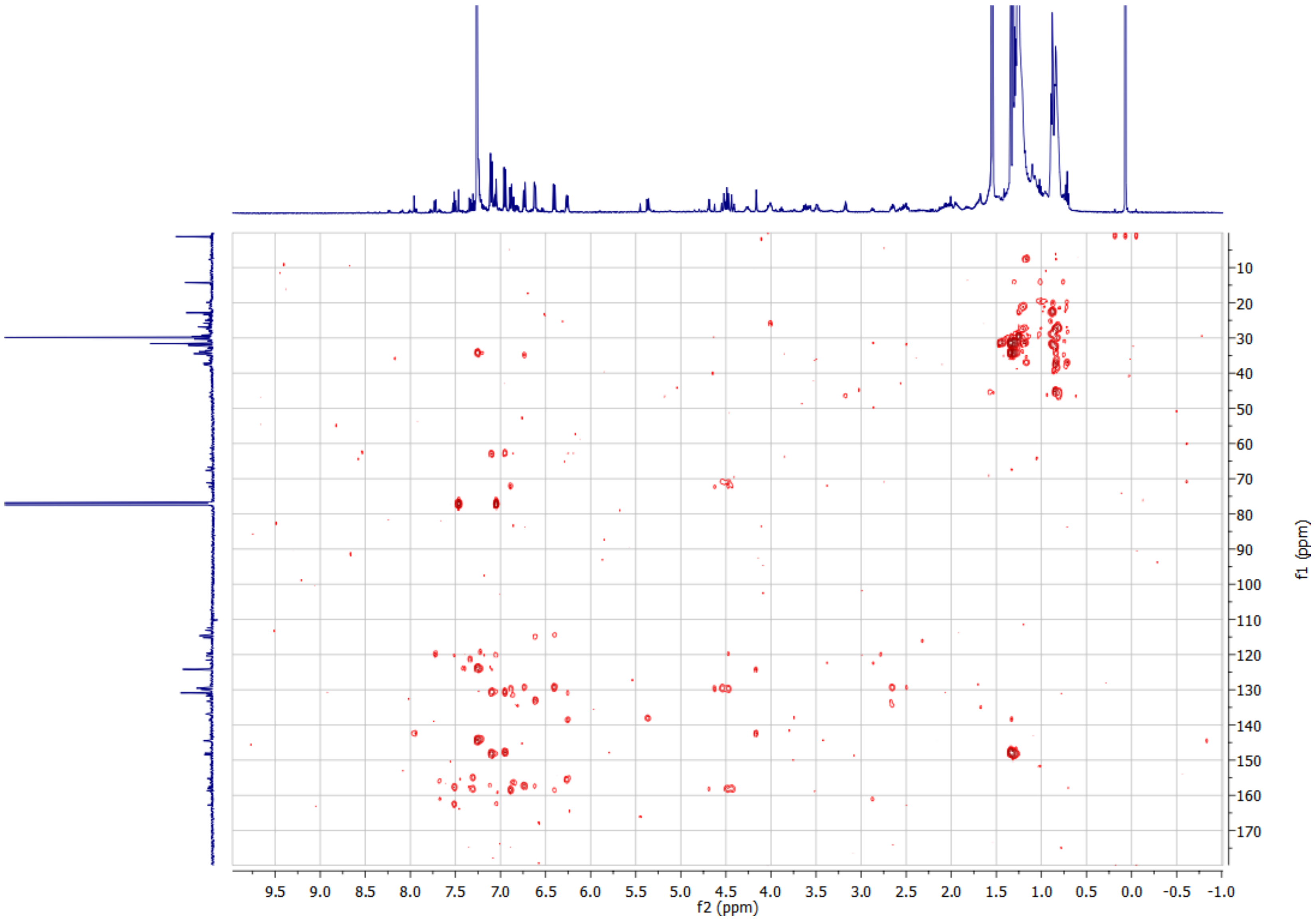

[3]Rotaxane 9b: ROESY NMR ($CDCl_3$, 500 MHz, 300 K)

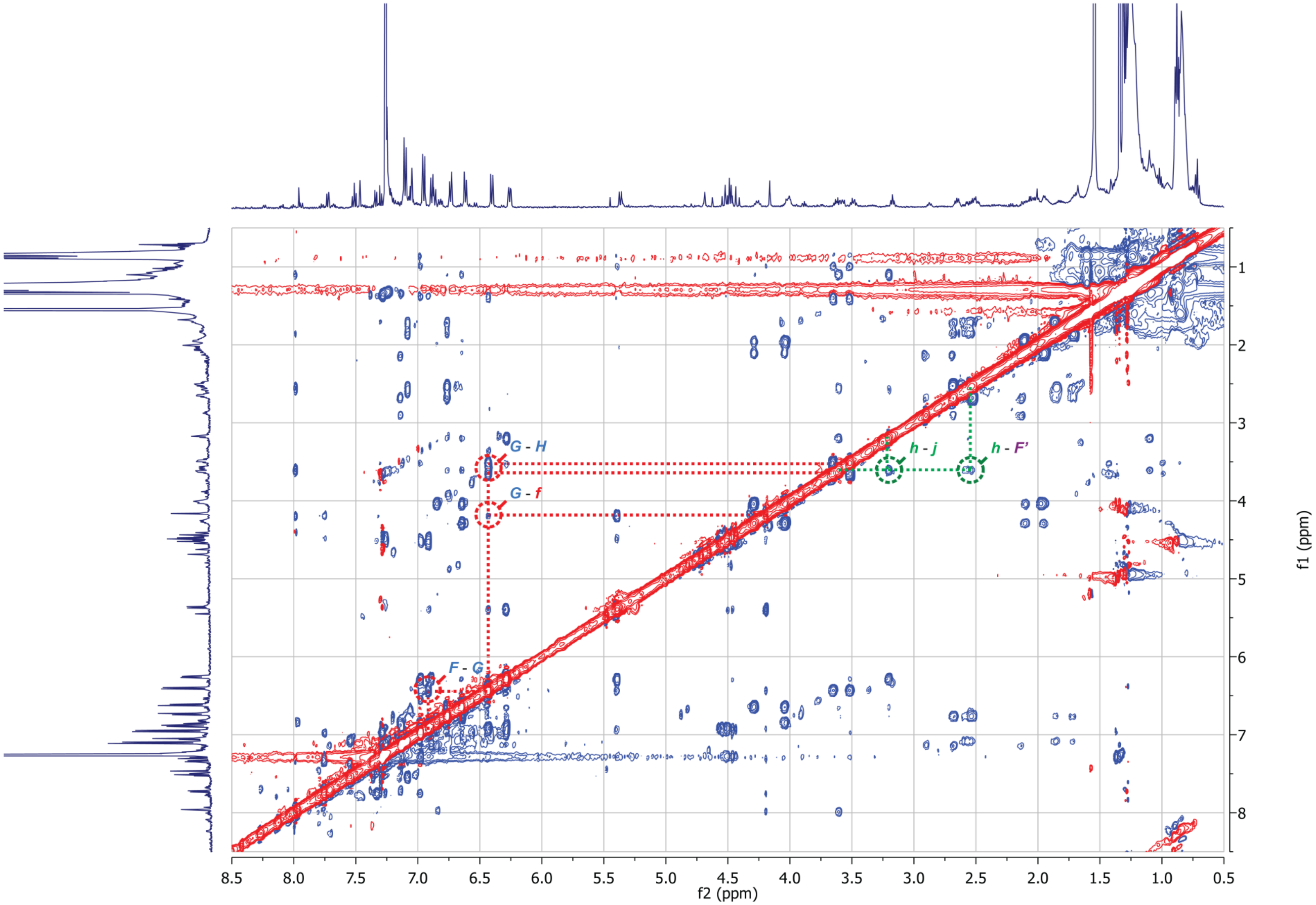

[3]Rotaxane 9c: ^{1}H NMR ($CDCl_3$, 600 MHz, 300 K)

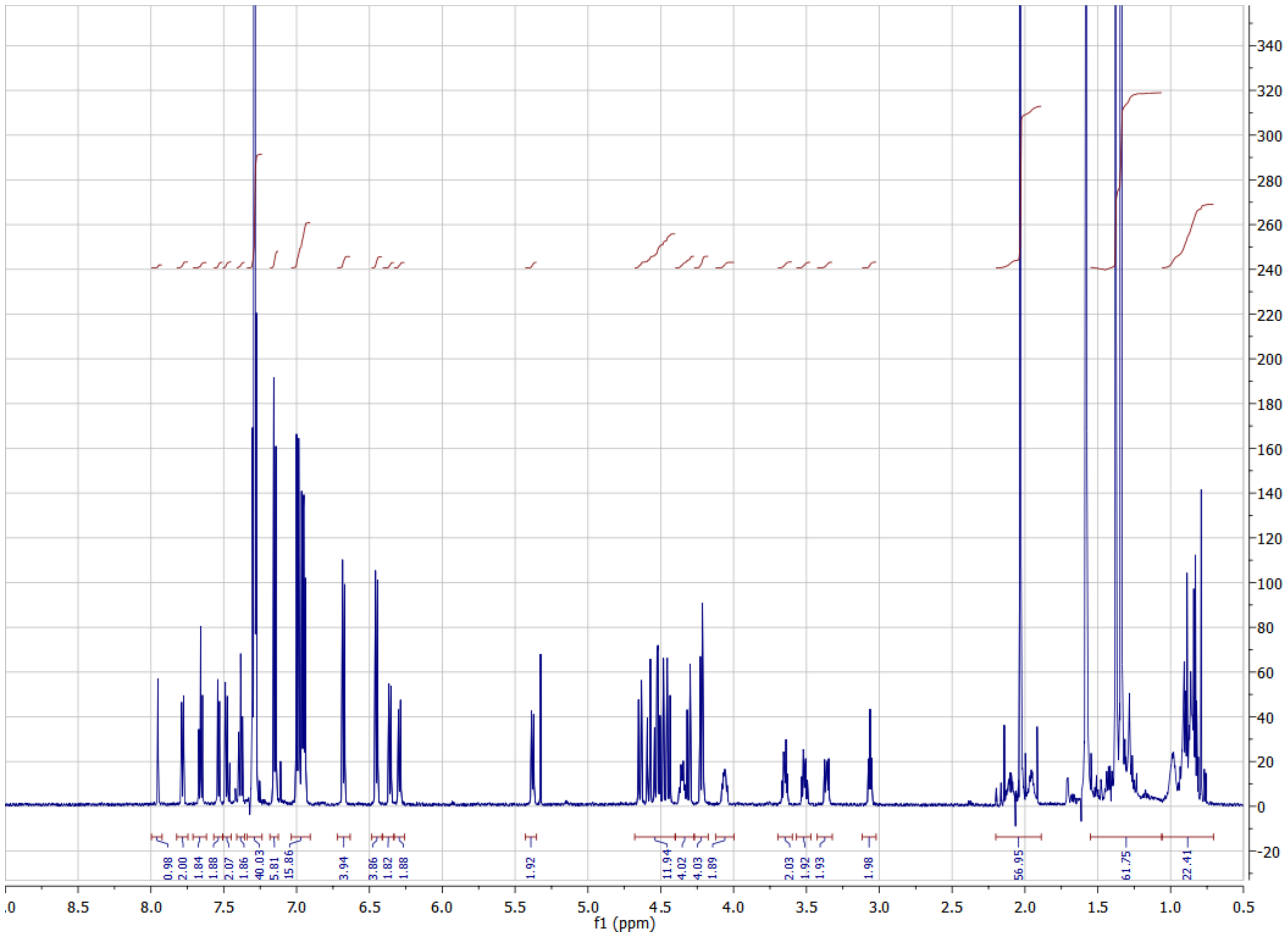

[3]Rotaxane 9c: ^{13}C NMR ($CDCl_3$, 125 MHz, 300 K)

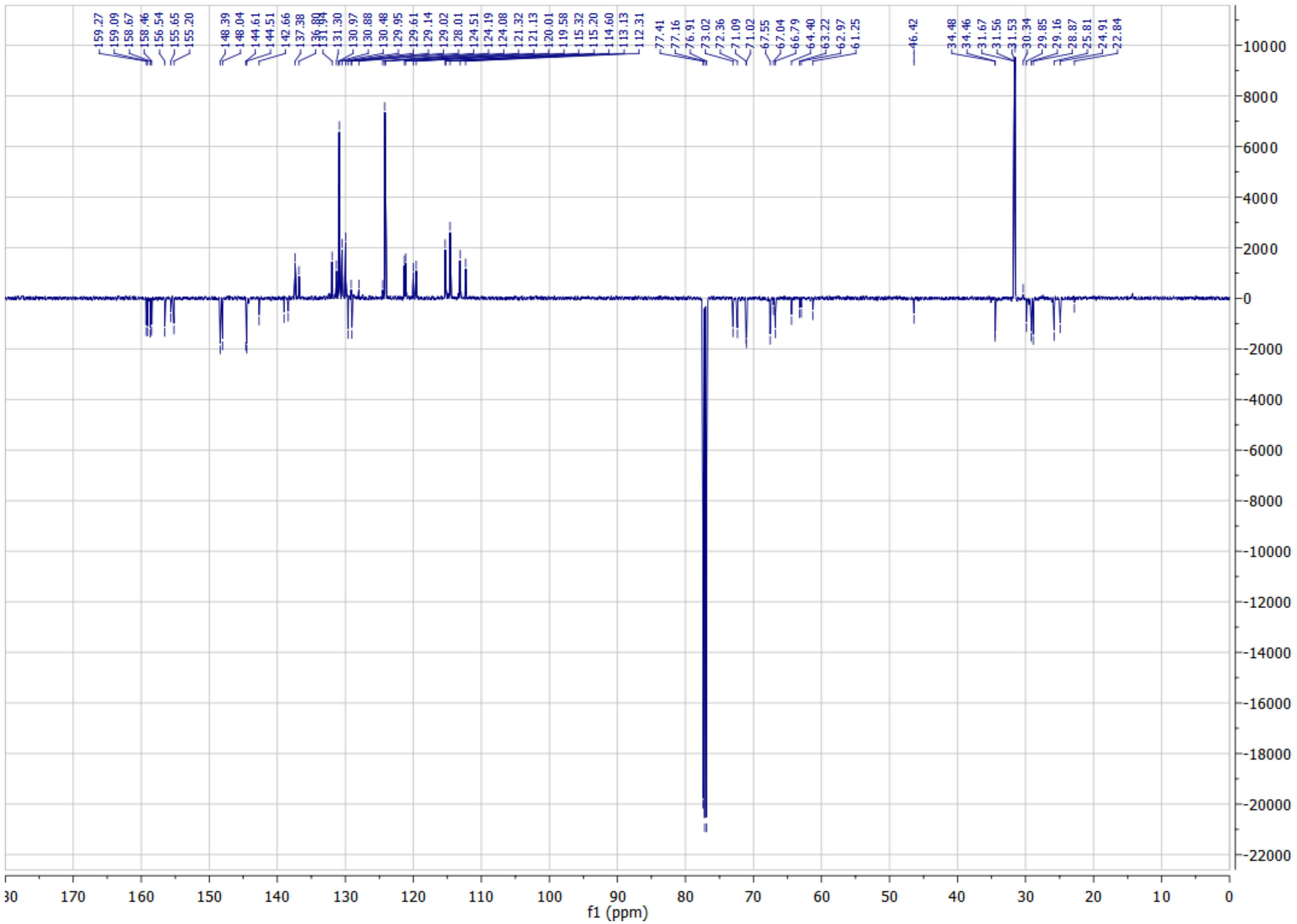

[3]Rotaxane 9c: COSY NMR ($CDCl_3$, 600 MHz, 300 K)

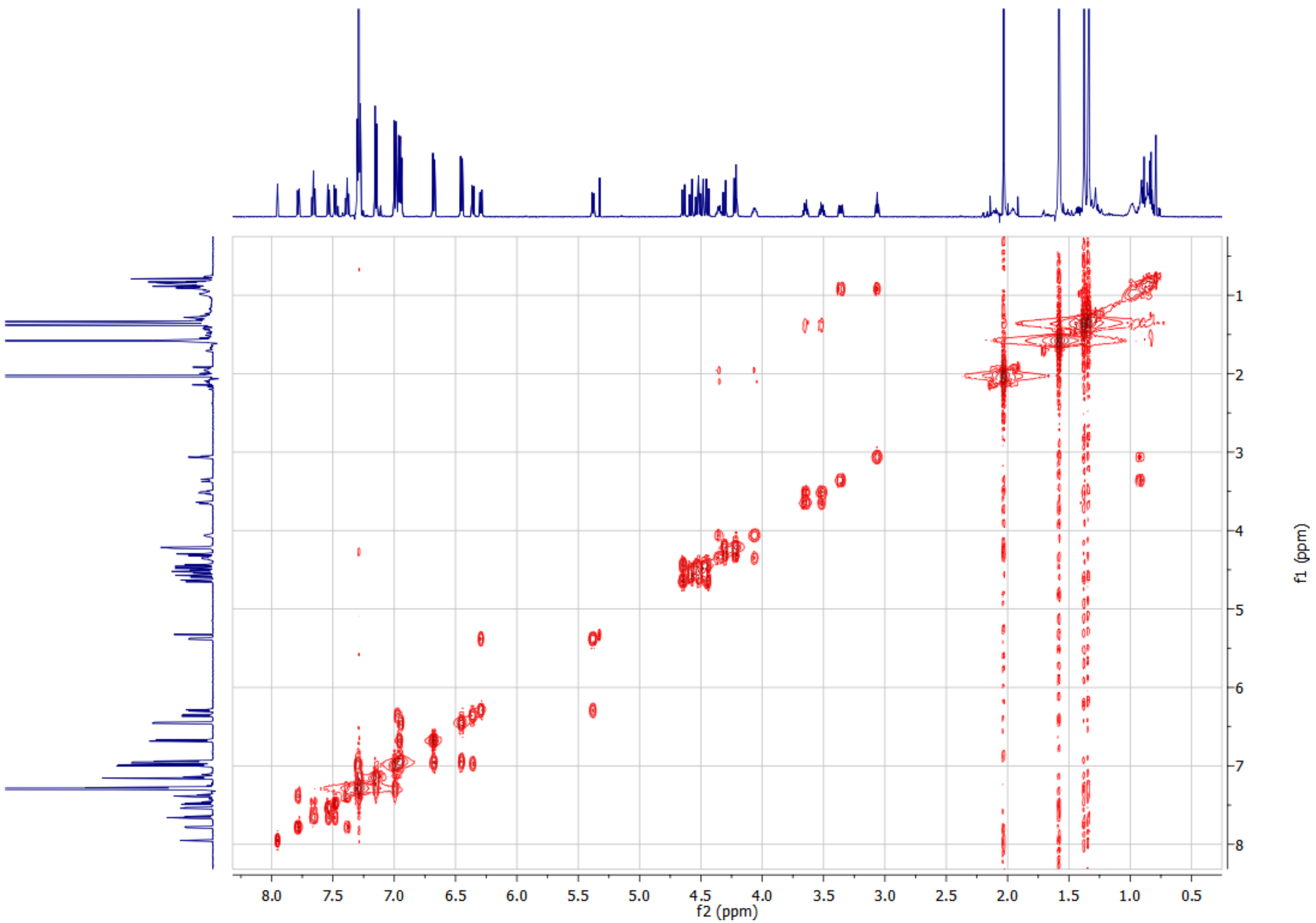

[3]Rotaxane 9c: HSQC NMR ($CDCl_3$, 600 MHz, 300 K)

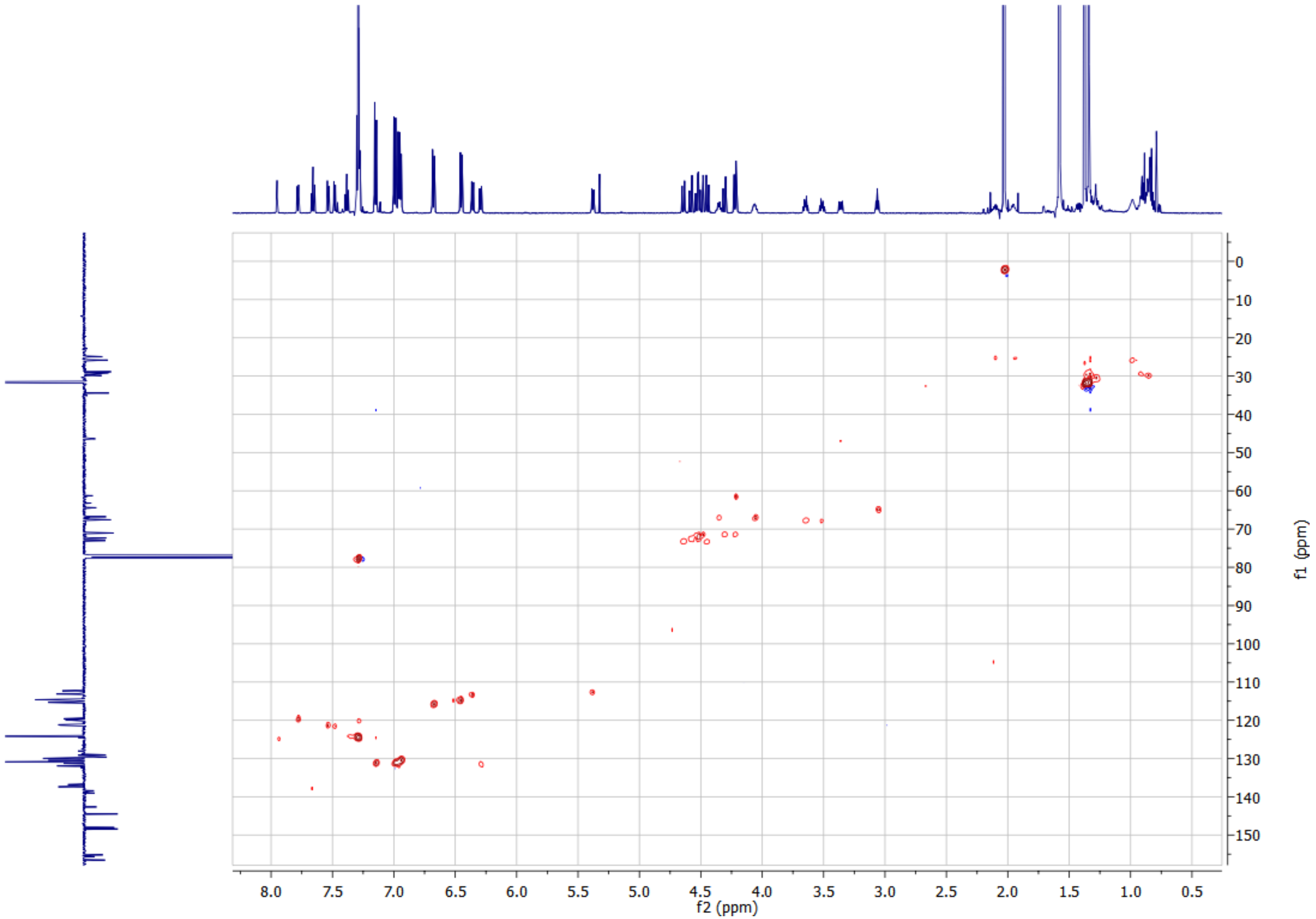

[3]Rotaxane 9c: HMBC NMR ($CDCl_3$, 600 MHz, 300 K)

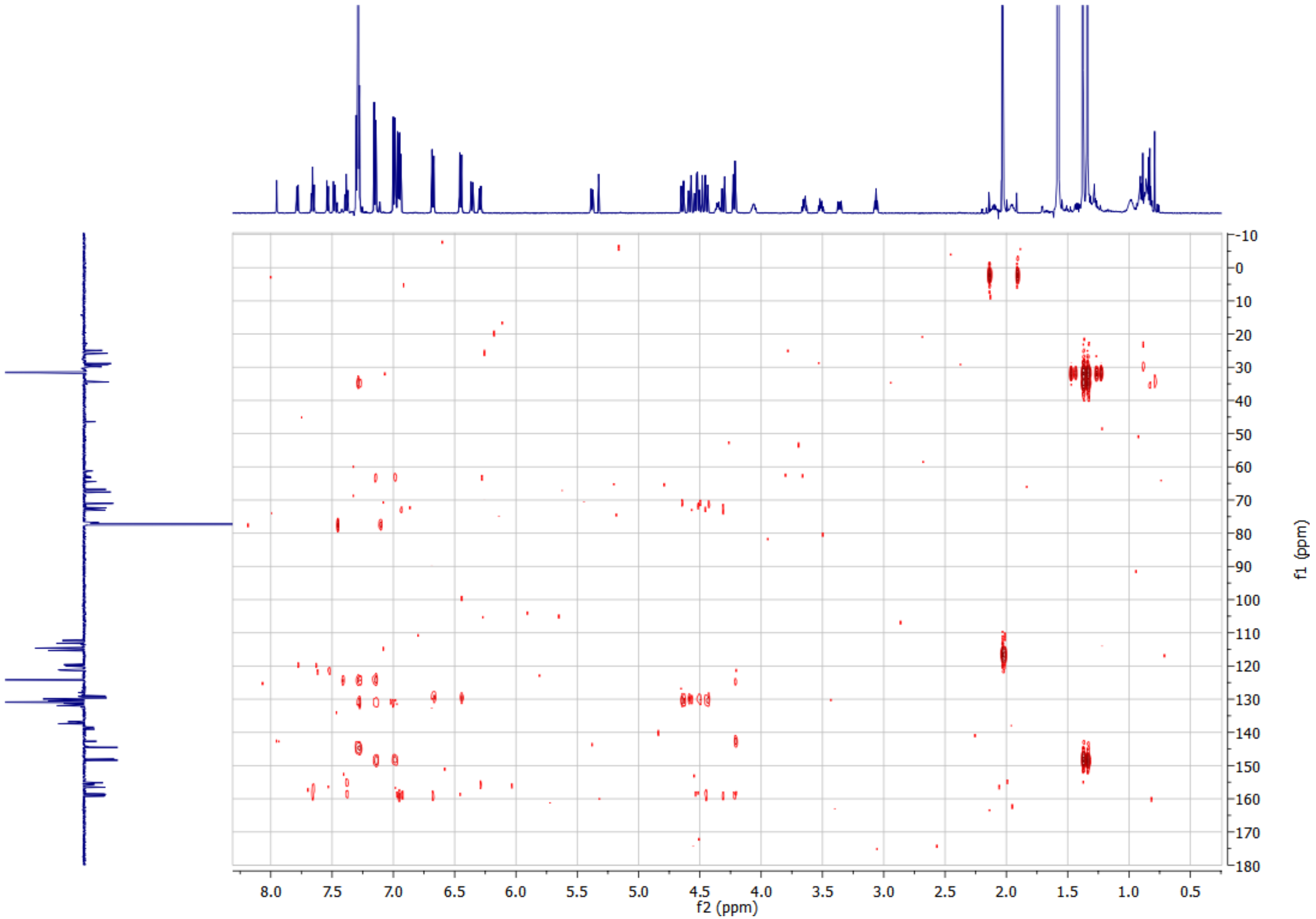

[3]Rotaxane 9c: ROESY NMR ($CDCl_3$, 600 MHz, 300 K)

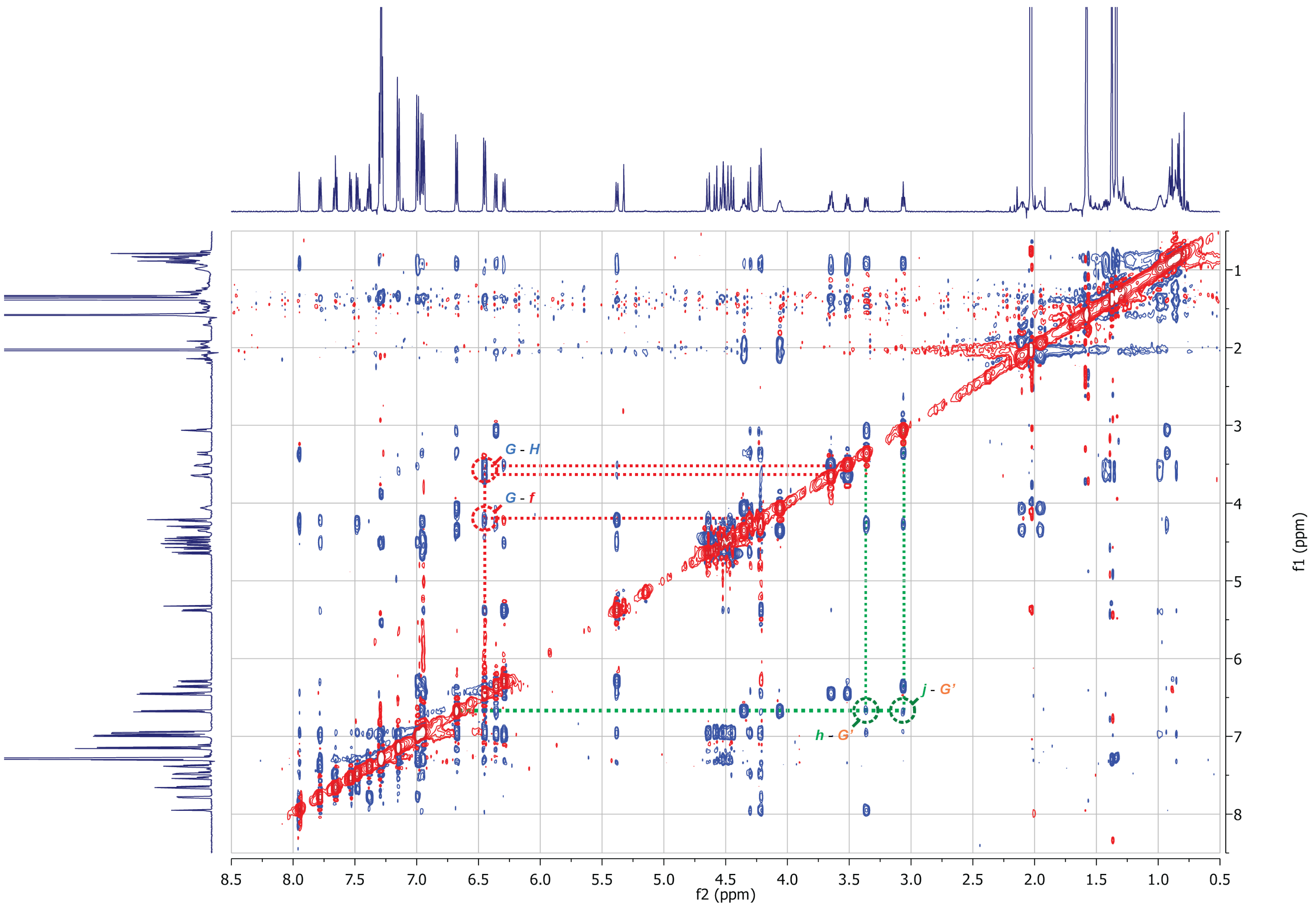

Dimethyl 5-(3-bromopropoxy)isophthalate (S14): ^1H NMR ($CDCl_3$, 500 MHz, 300 K)

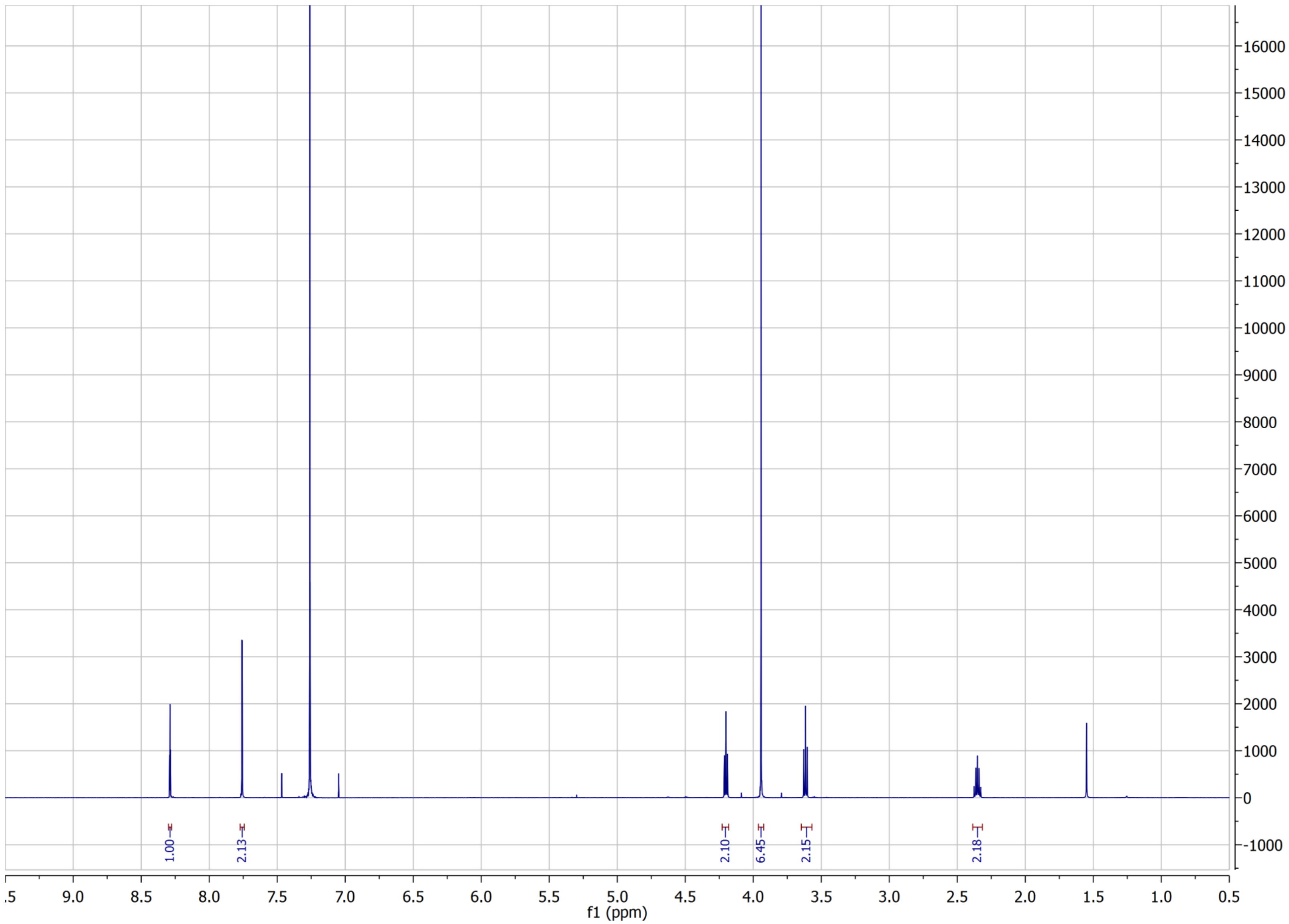

Dimethyl 5-(3-bromopropoxy)isophthalate (S14): ^{13}C NMR ($CDCl_3$, 125 MHz, 300 K)

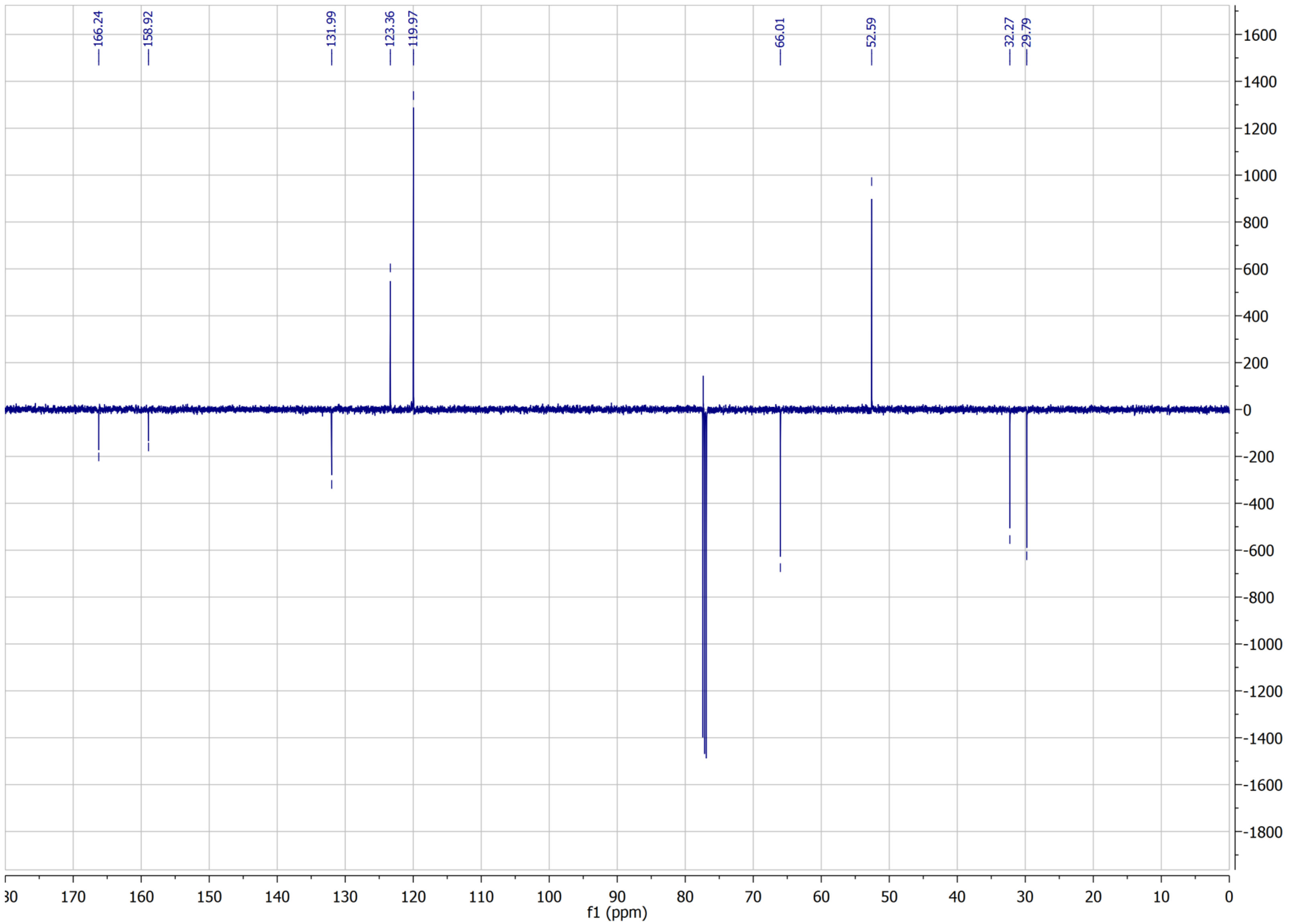

Azide 11c: ^{1}H NMR ($CDCl_3$, 500 MHz, 300 K)

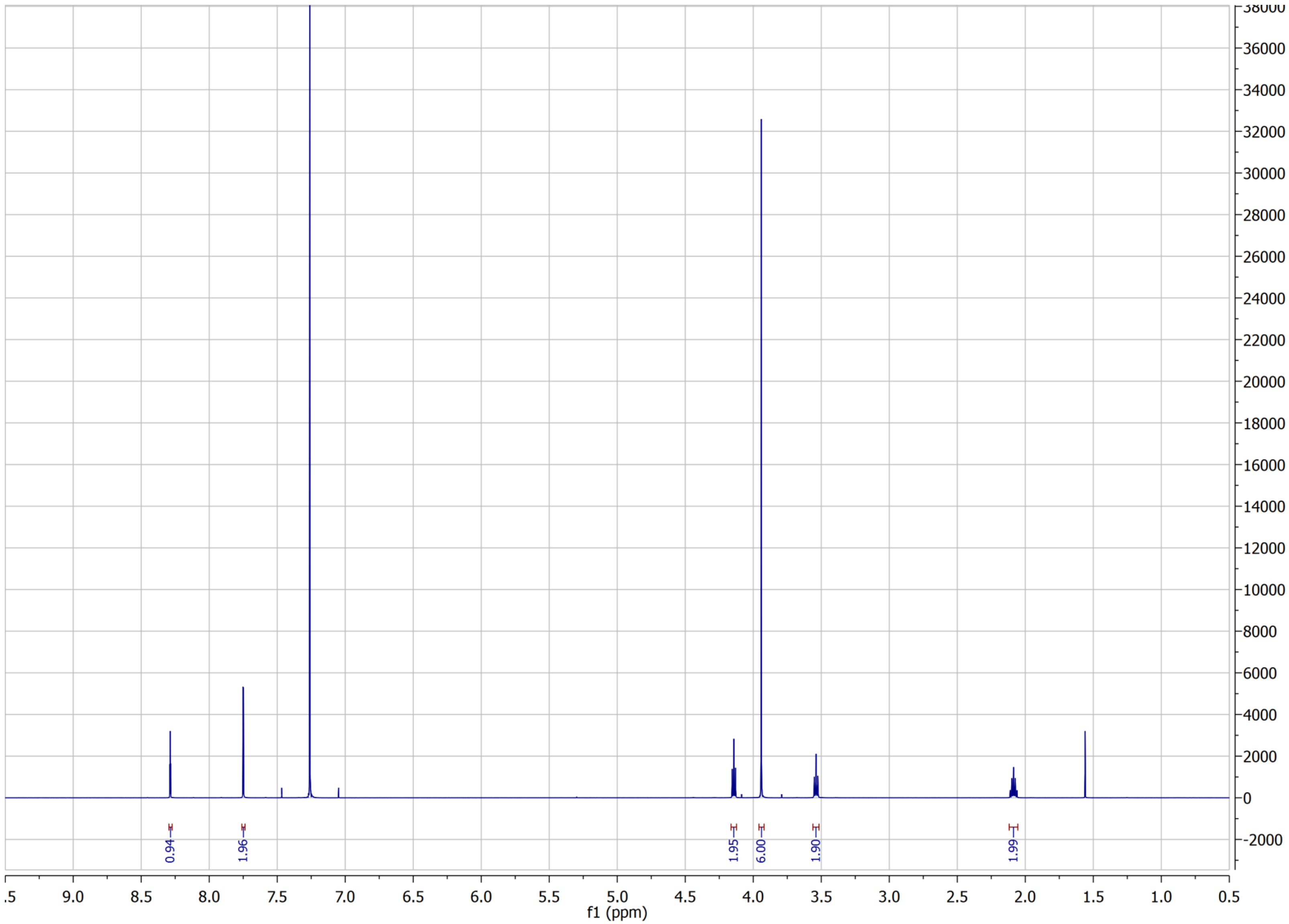

Azide 11c: ^{13}C NMR ($CDCl_3$, 125 MHz, 300 K)

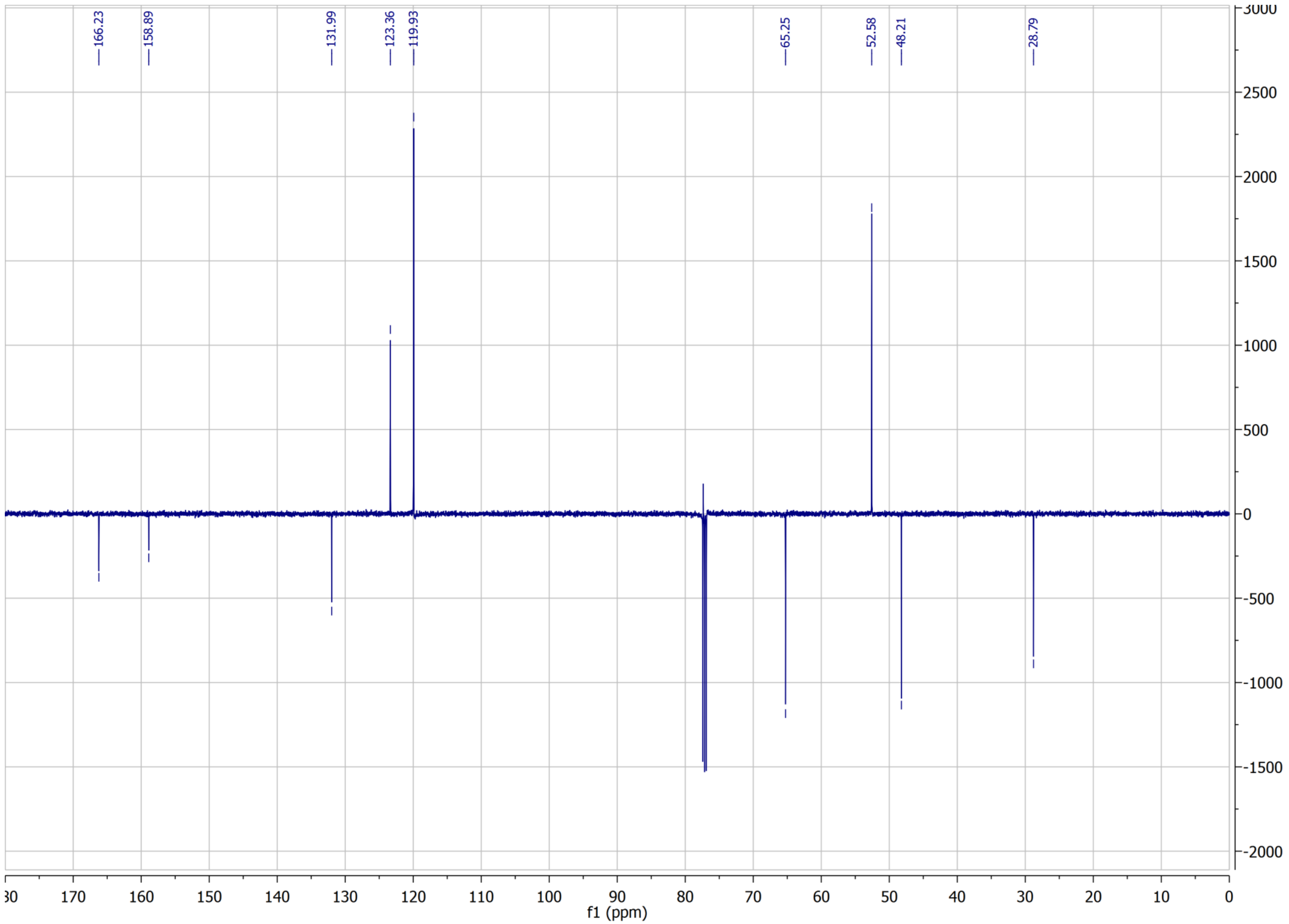

[2]Rotaxane S3: ^{1}H NMR ($CDCl_3$, 500 MHz, 300 K)

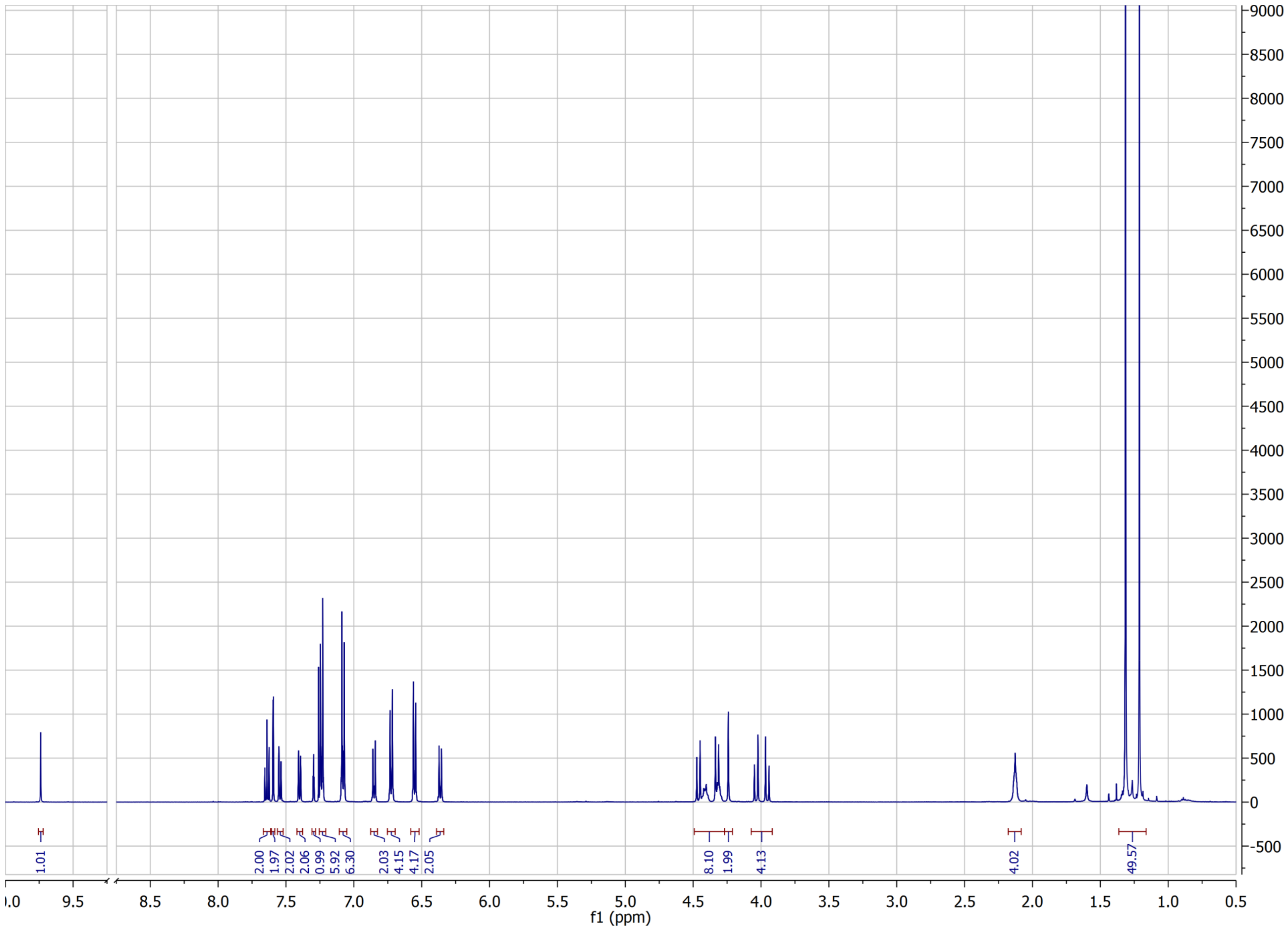

[2]Rotaxane S3: ^{13}C NMR ($CDCl_3$, 125 MHz, 300 K)

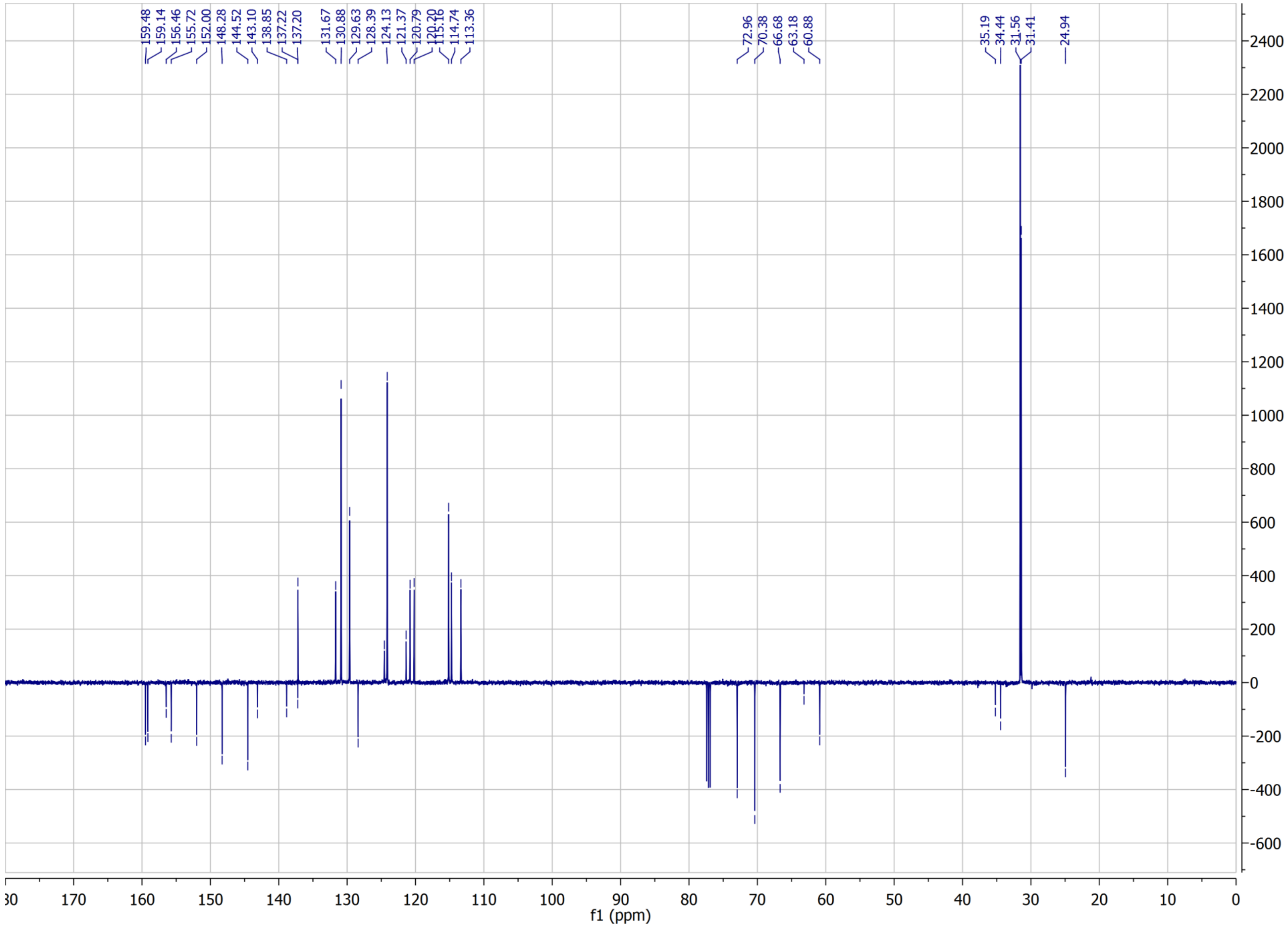

[2]Rotaxane S3: COSY NMR ($CDCl_3$, 500 MHz, 300 K)

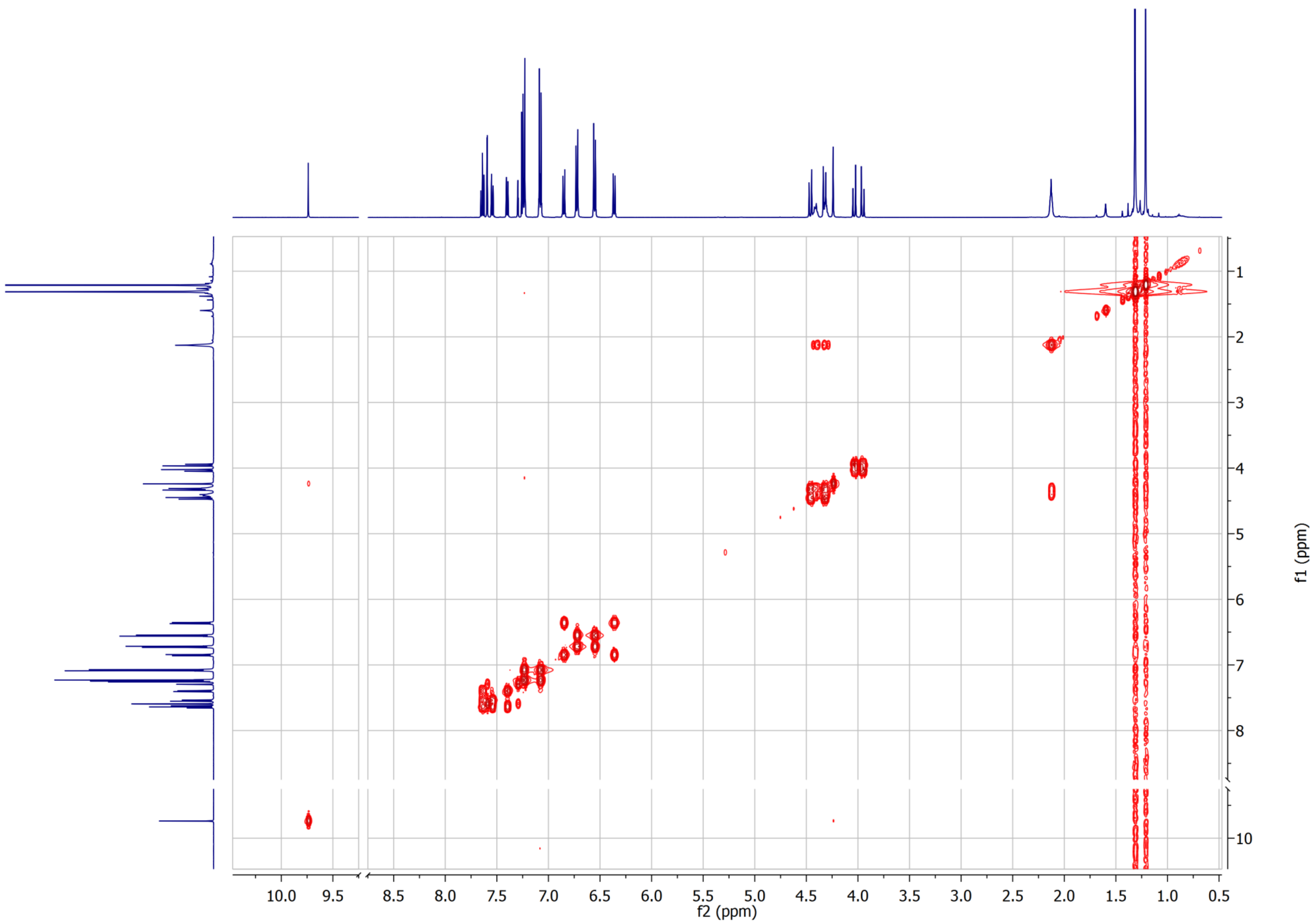

[2]Rotaxane S3: HSQC NMR ($CDCl_3$, 500 MHz, 300 K)

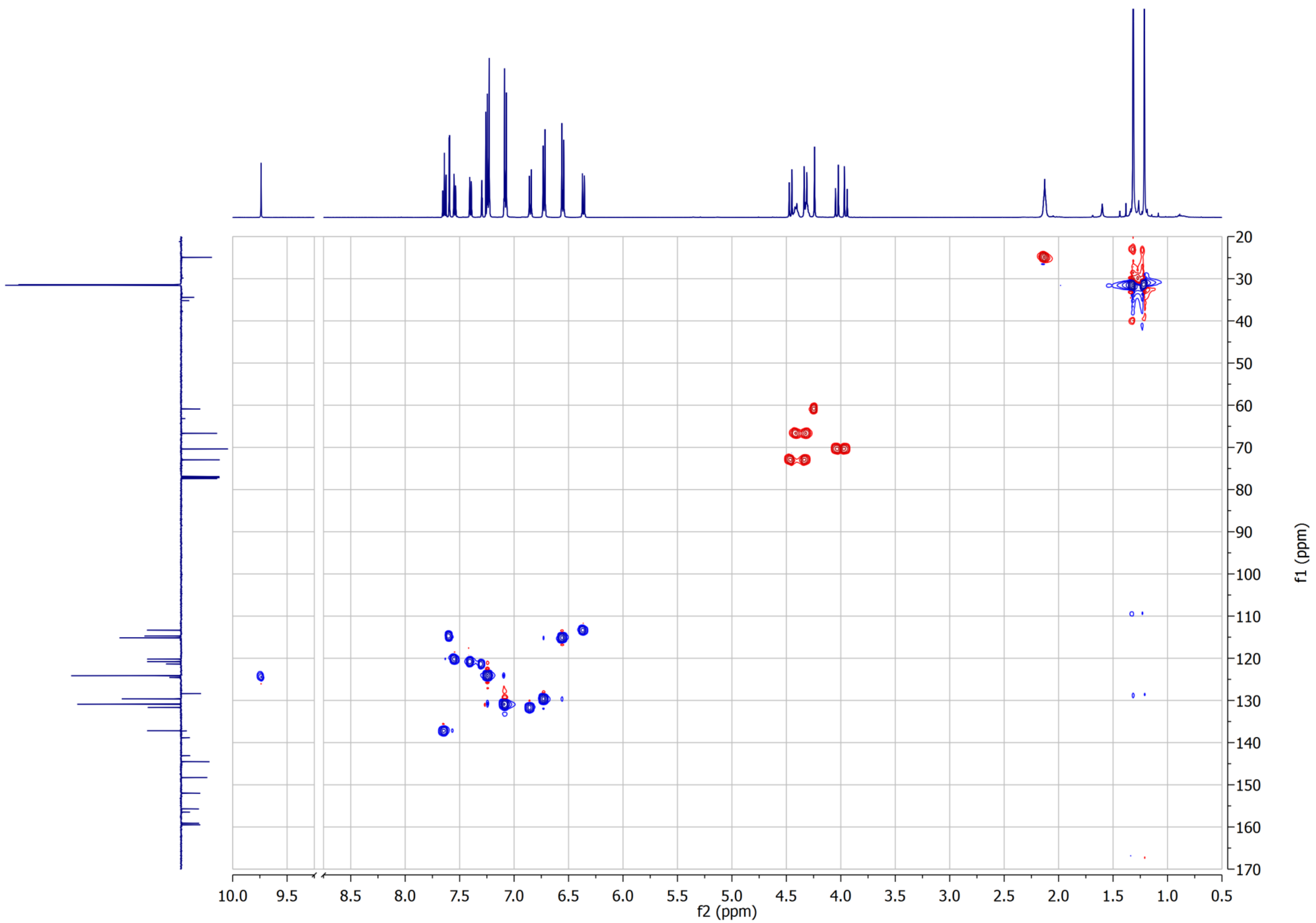

[2]Rotaxane S3: HMBC NMR ($CDCl_3$, 500 MHz, 300 K)

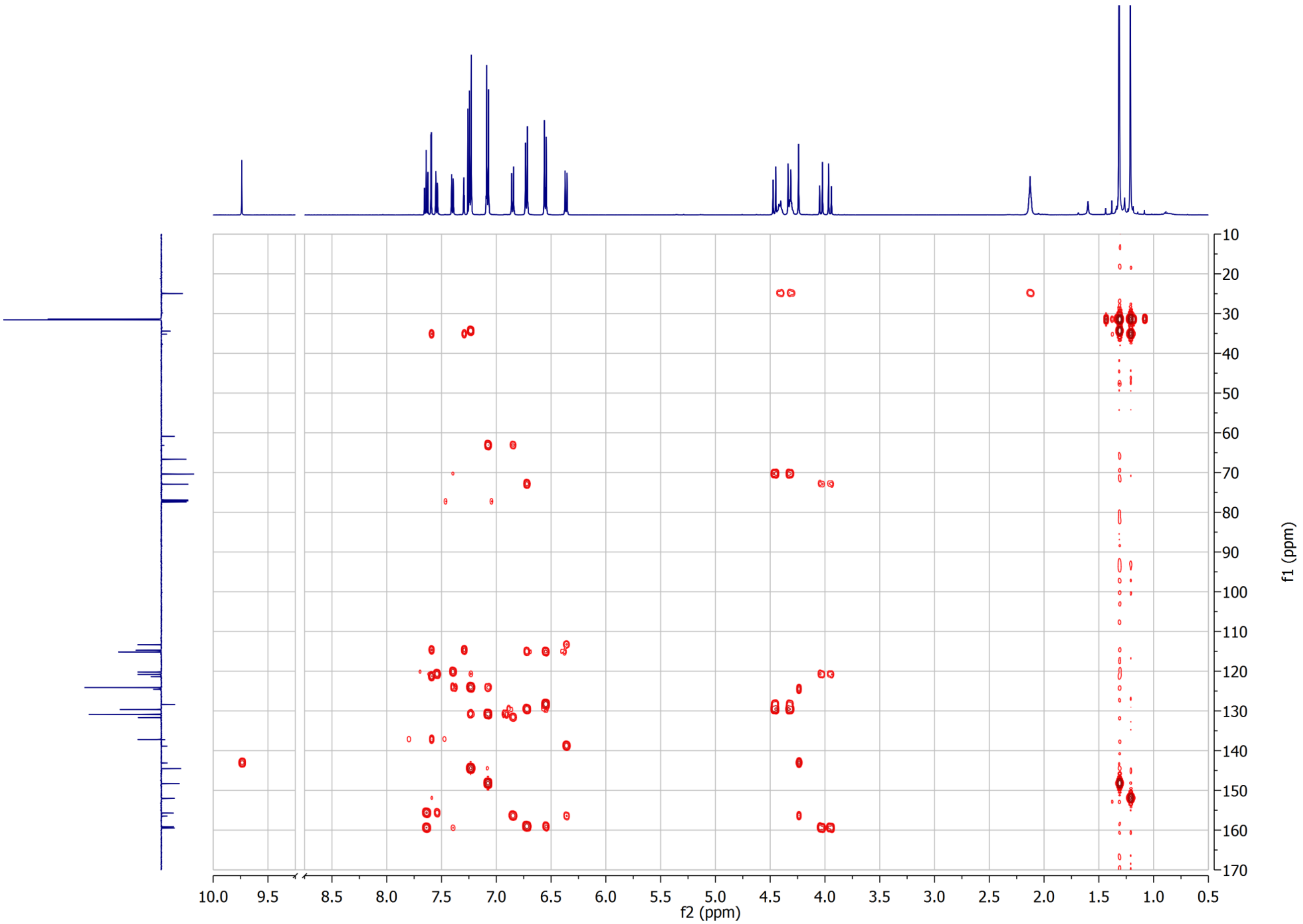

[2]Rotaxane S4: ^{1}H NMR ($CDCl_3$, 500 MHz, 300 K)

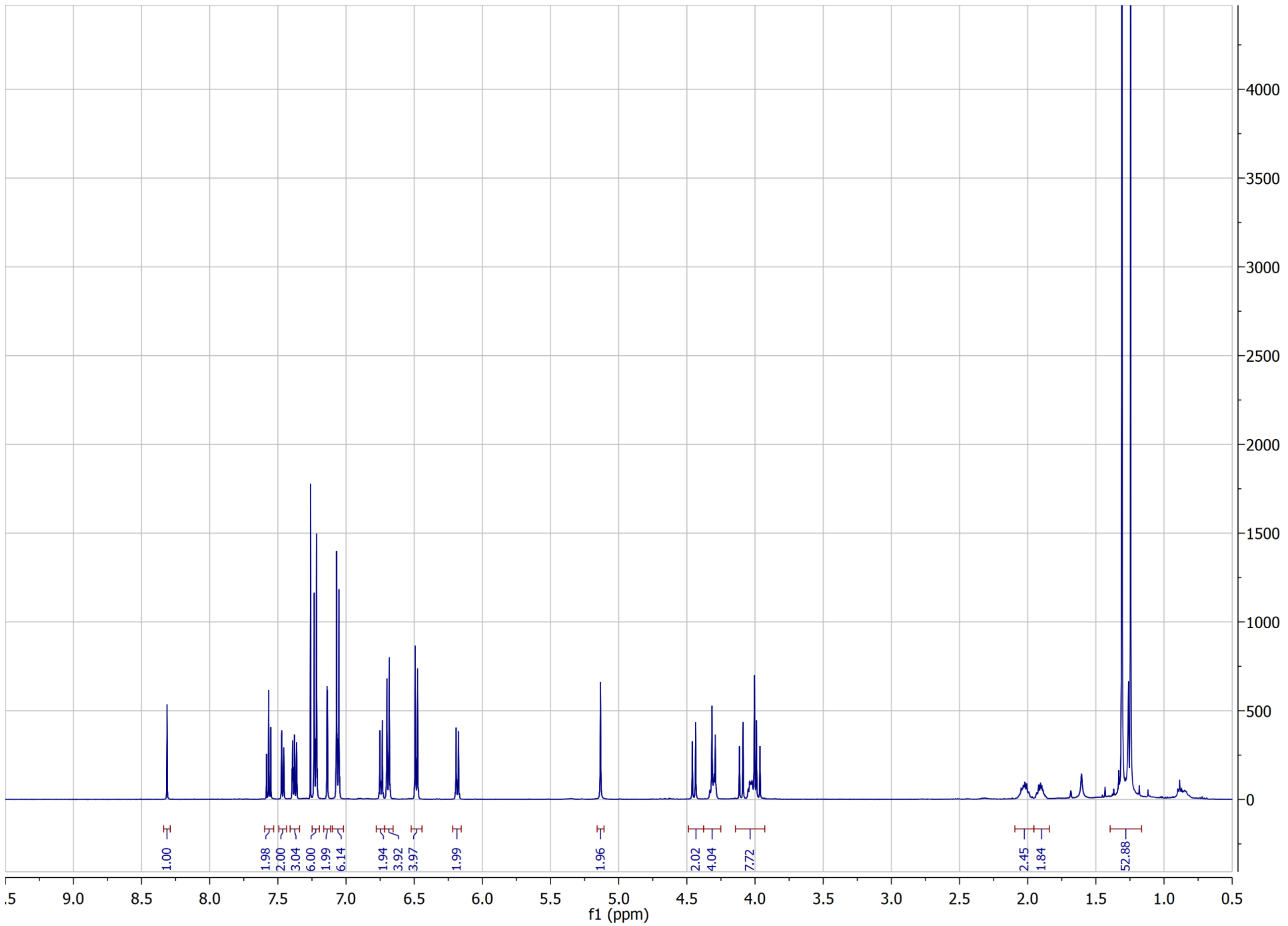

[2]Rotaxane S4: ^{13}C NMR ($CDCl_3$, 125 MHz, 300 K)

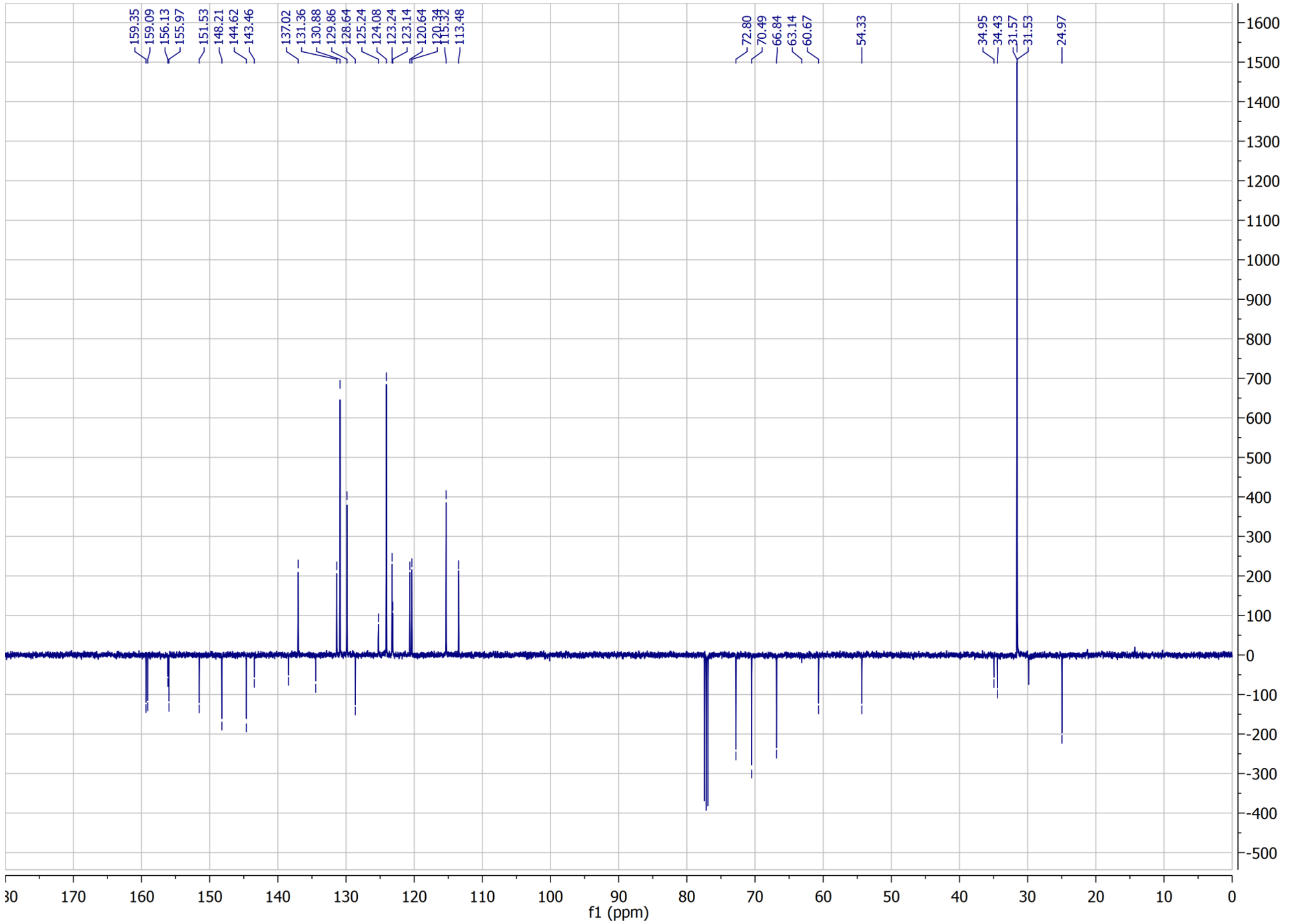

[2]Rotaxane S4: COSY NMR ($CDCl_3$, 500 MHz, 300 K)

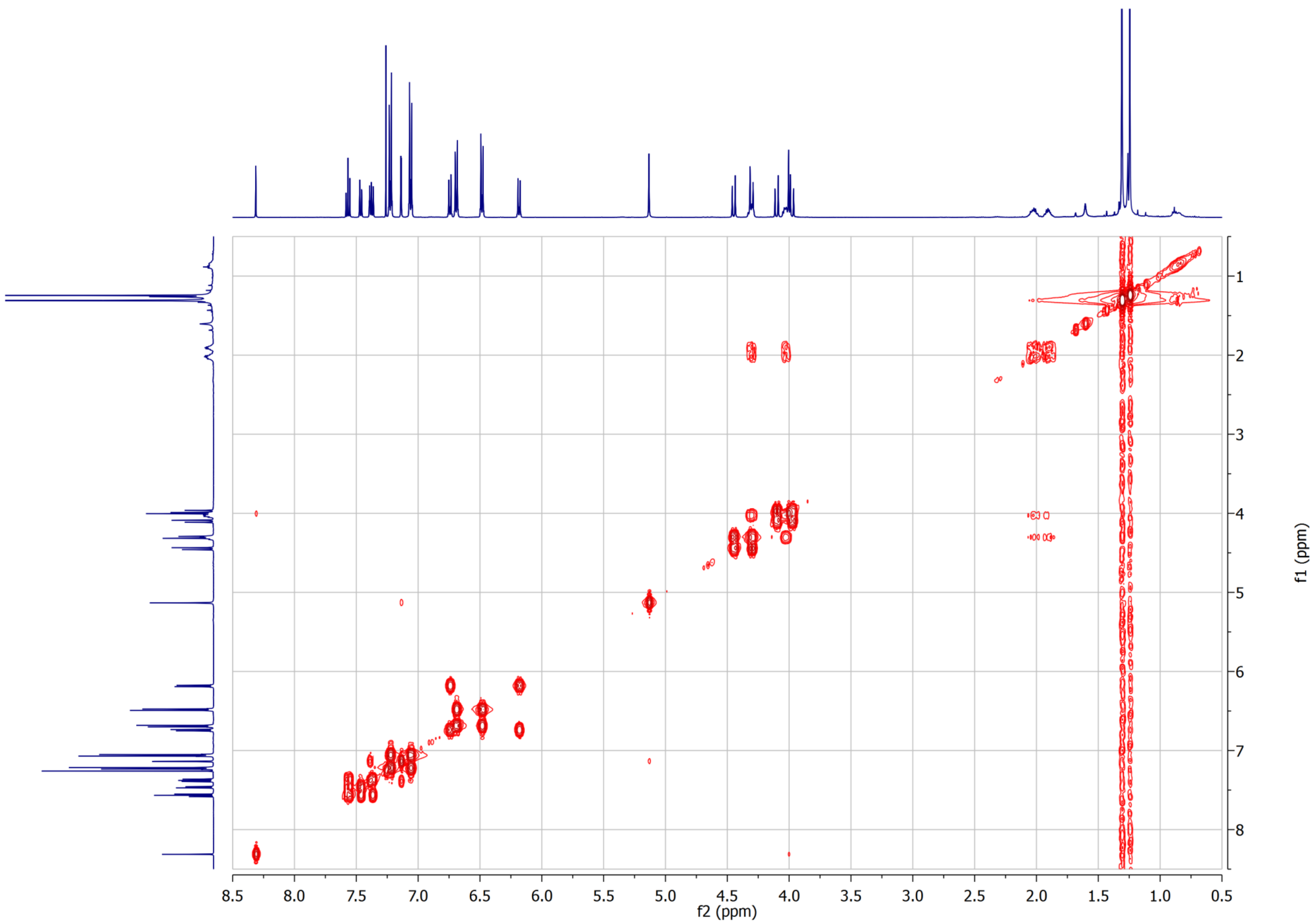

[2]Rotaxane S4: HSQC NMR ($CDCl_3$, 500 MHz, 300 K)

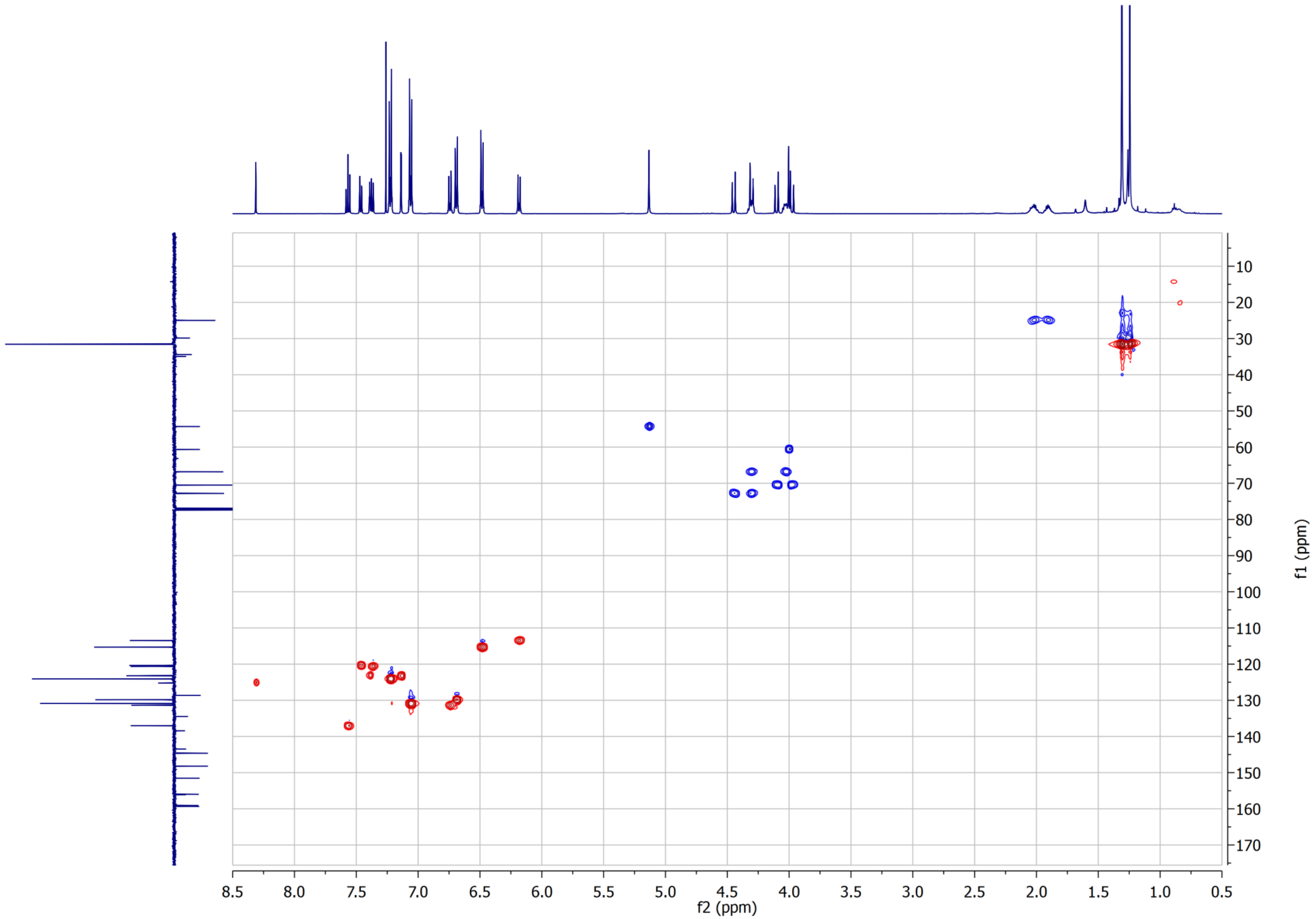

[2]Rotaxane S4: HMBC NMR ($CDCl_3$, 500 MHz, 300 K)

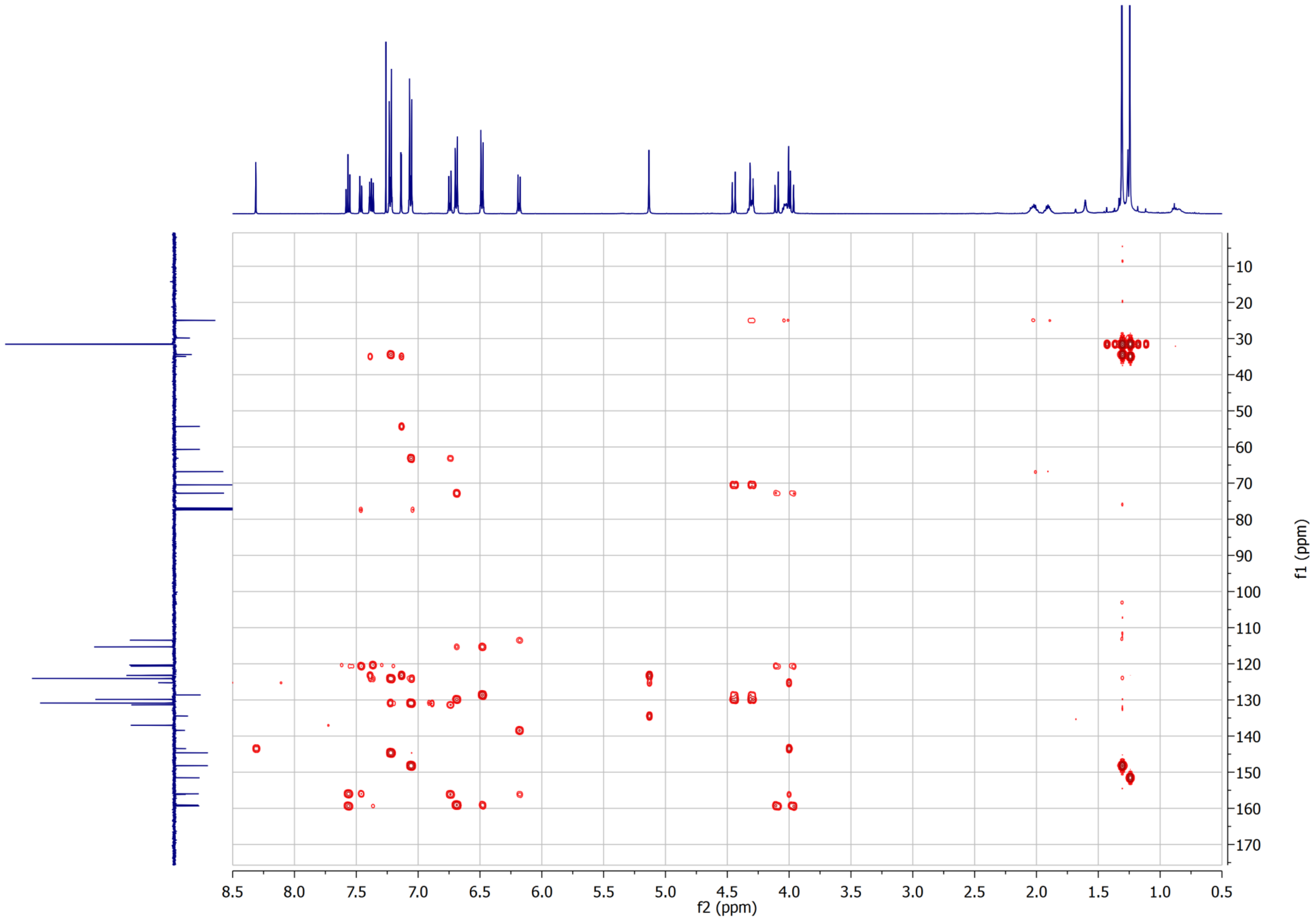

[2]Rotaxane S5: ^{1}H NMR ($CDCl_3$, 500 MHz, 300 K)

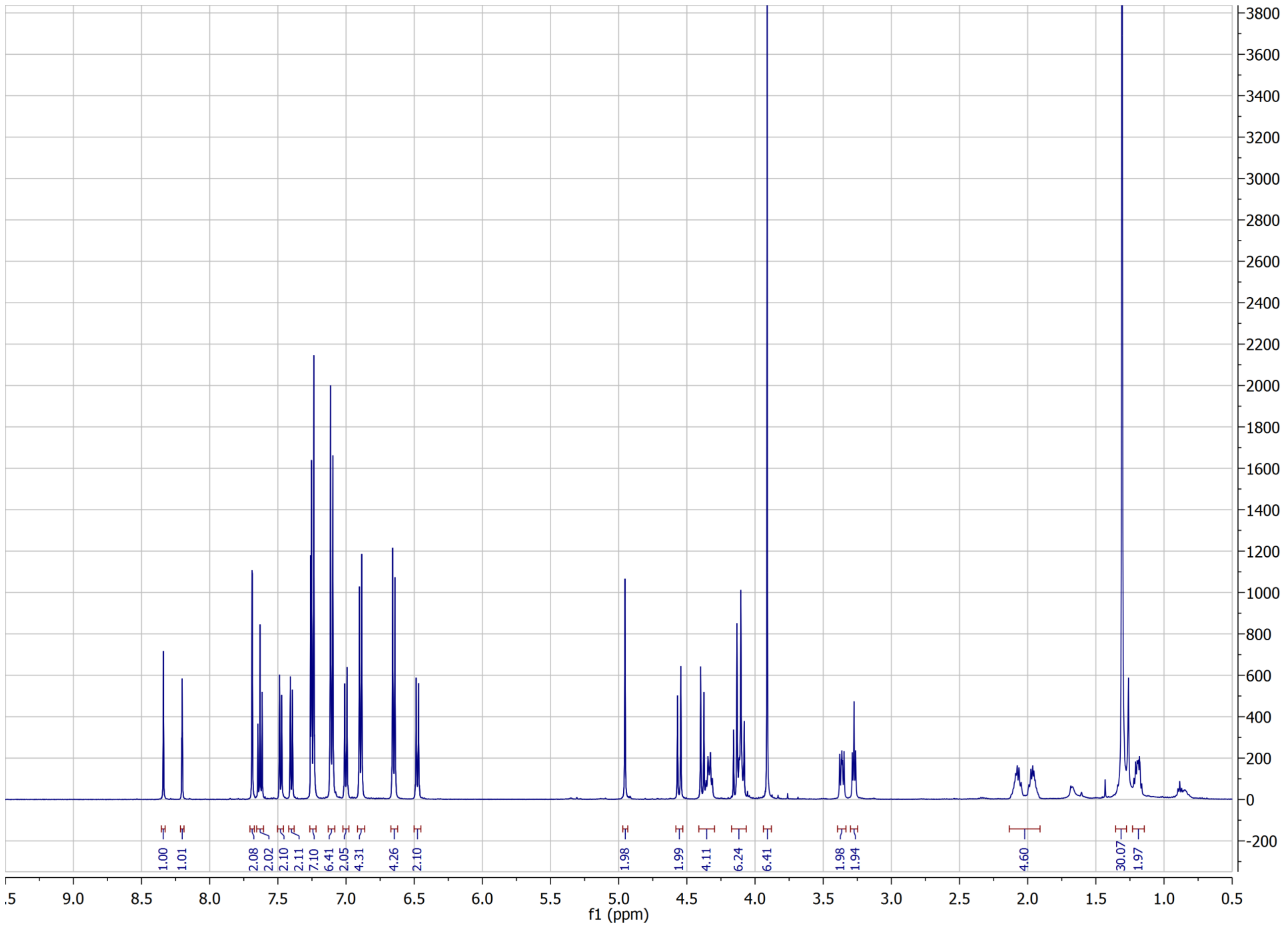

[2]Rotaxane S5: ^{13}C NMR ($CDCl_3$, 125 MHz, 300 K)

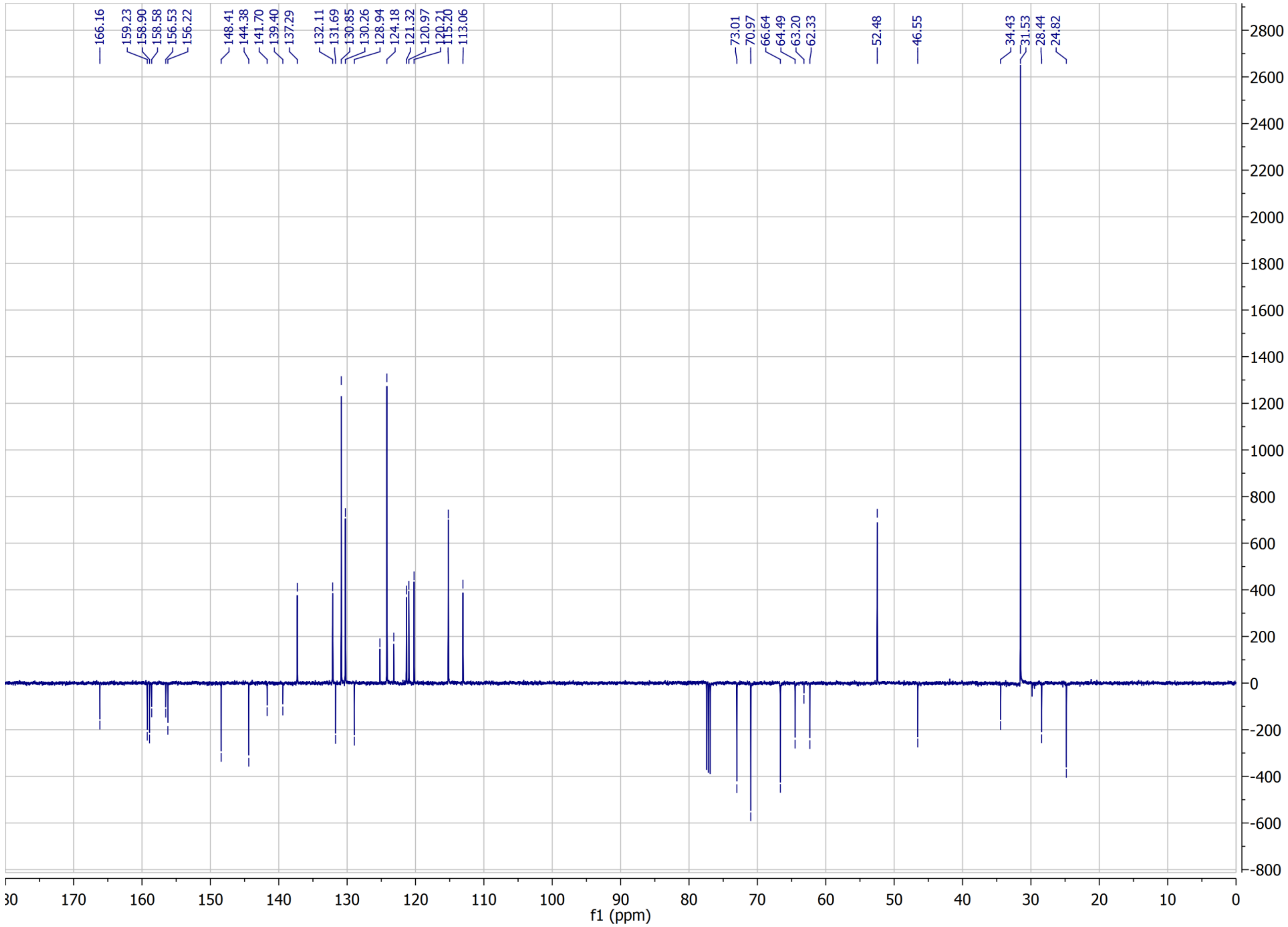

[2]Rotaxane S5: COSY NMR ($CDCl_3$, 500 MHz, 300 K)

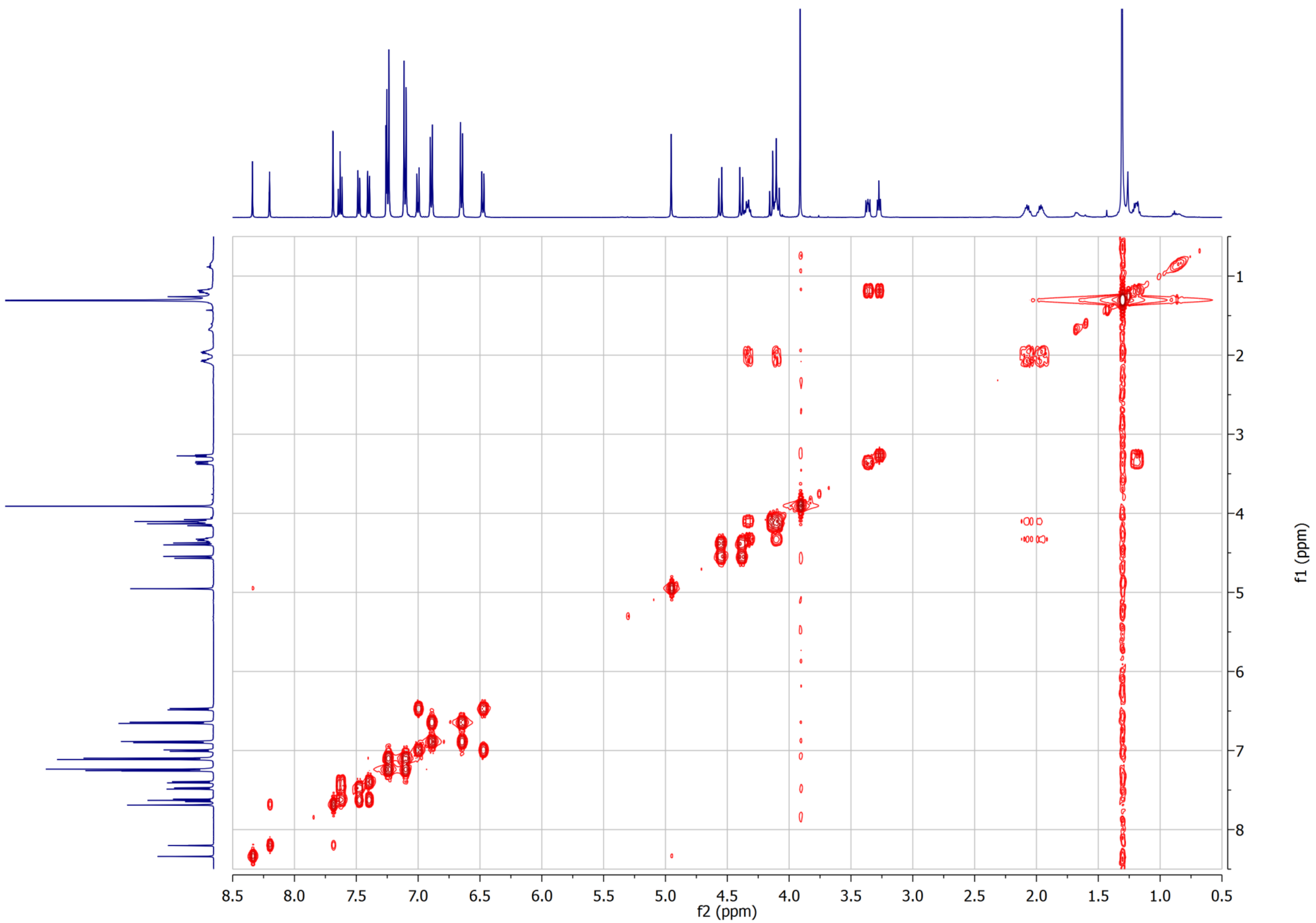

[2]Rotaxane S5: HSQC NMR ($CDCl_3$, 500 MHz, 300 K)

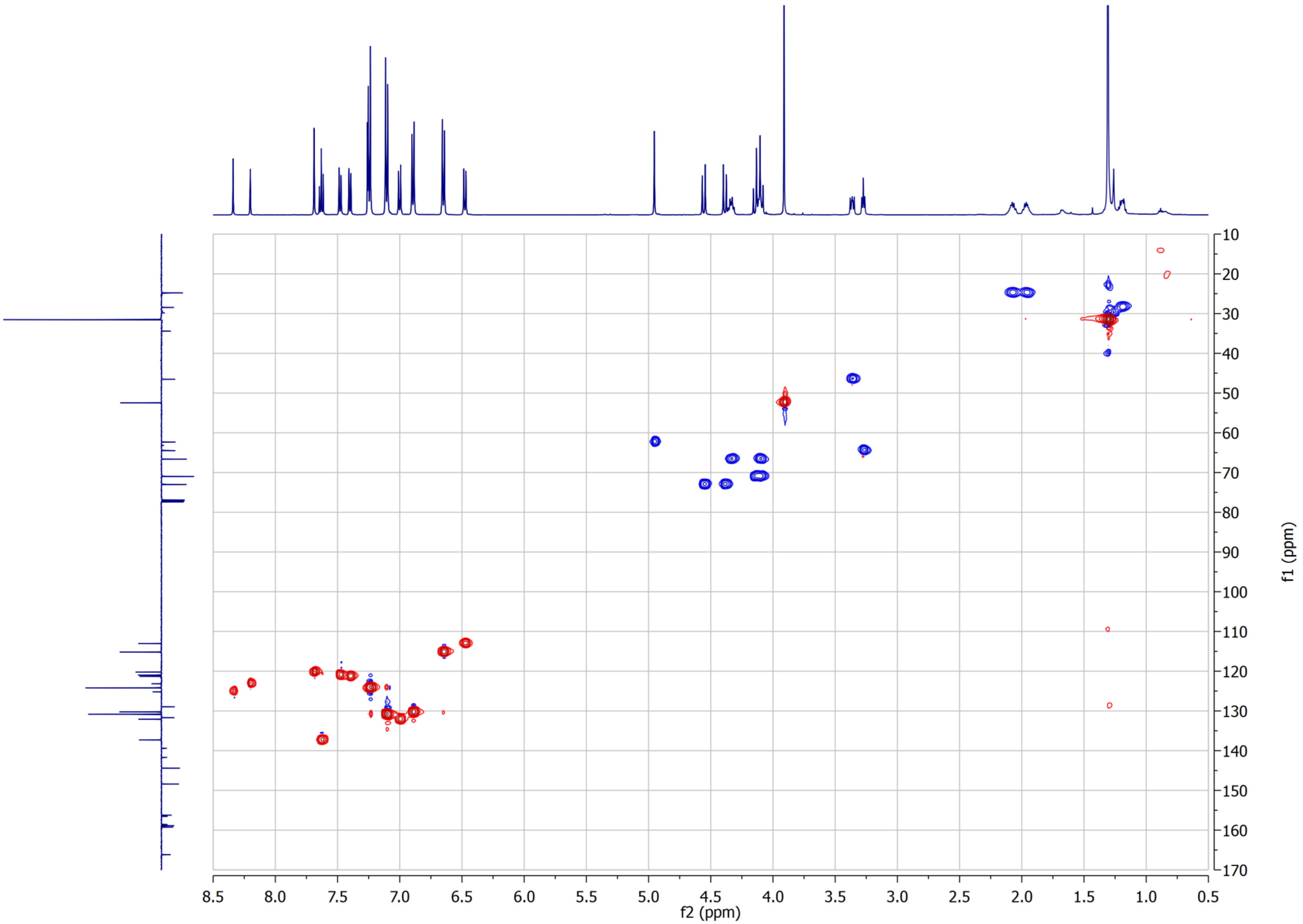

[2]Rotaxane S5: HMBC NMR ($CDCl_3$, 500 MHz, 300 K)

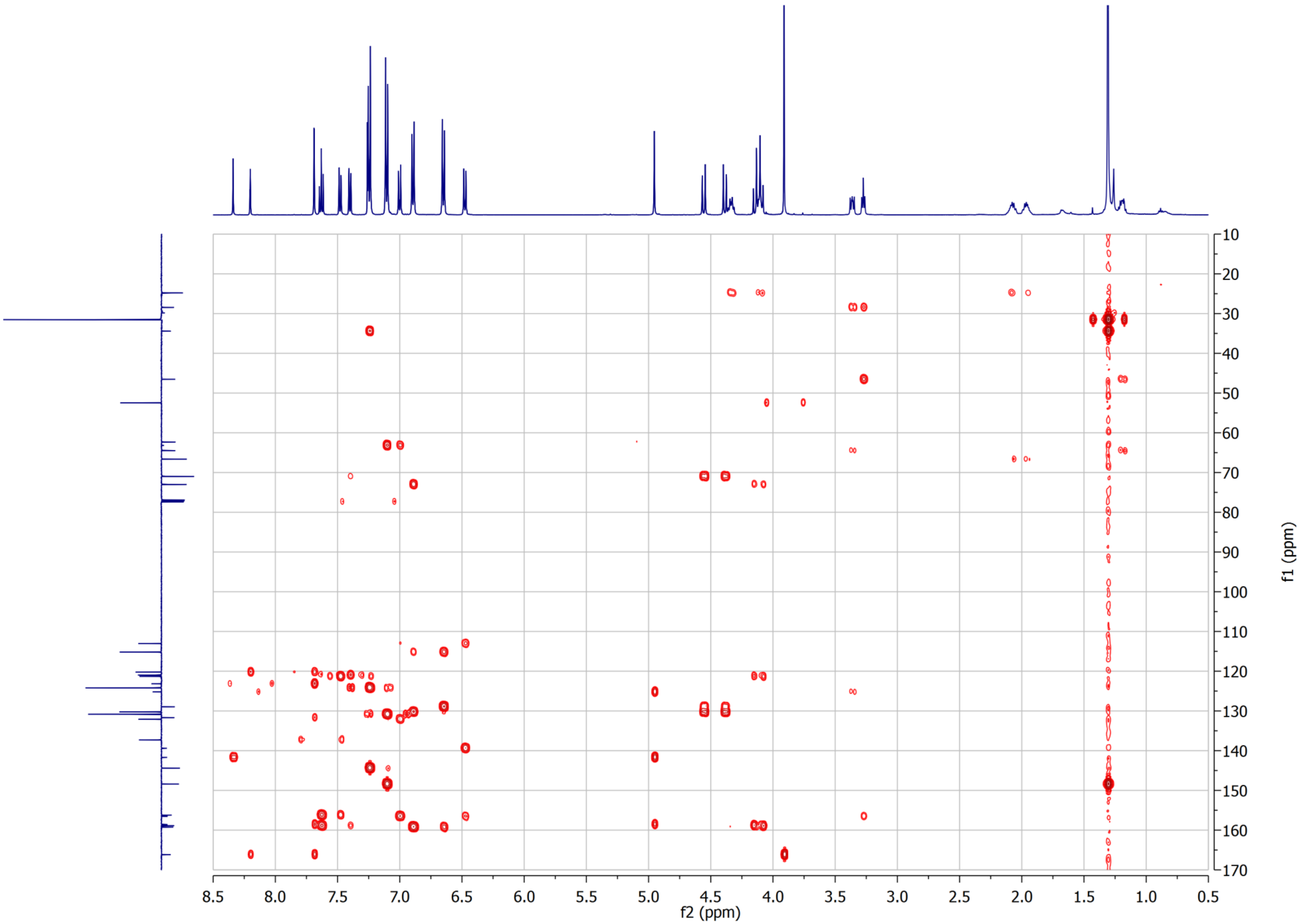

Thread S6: ^{1}H NMR ($CDCl_3$, 500 MHz, 300 K)

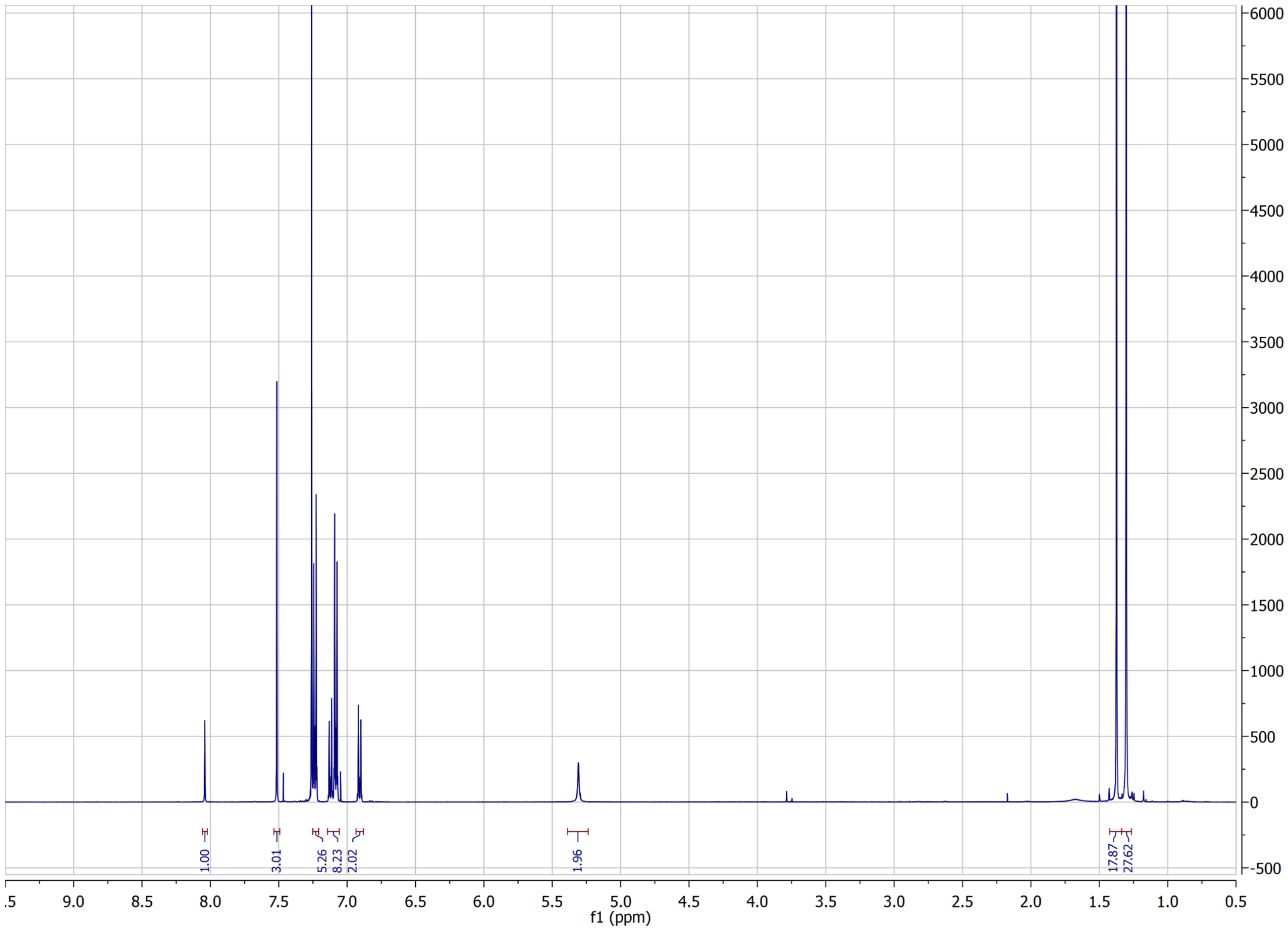

Thread S6: ^{13}C NMR ($CDCl_3$, 125 MHz, 300 K)

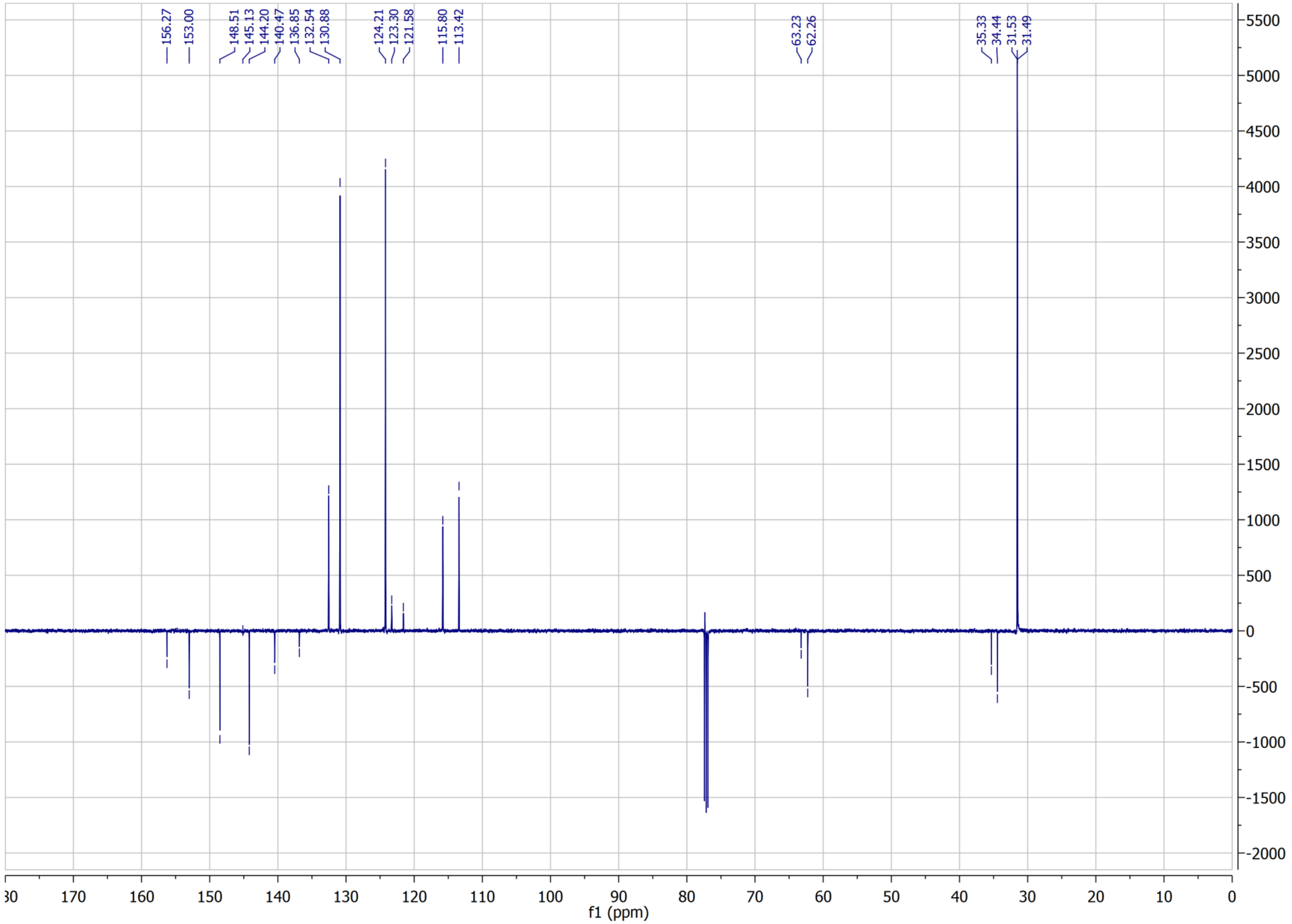

Thread S7: ^{1}H NMR ($CDCl_3$, 500 MHz, 300 K)

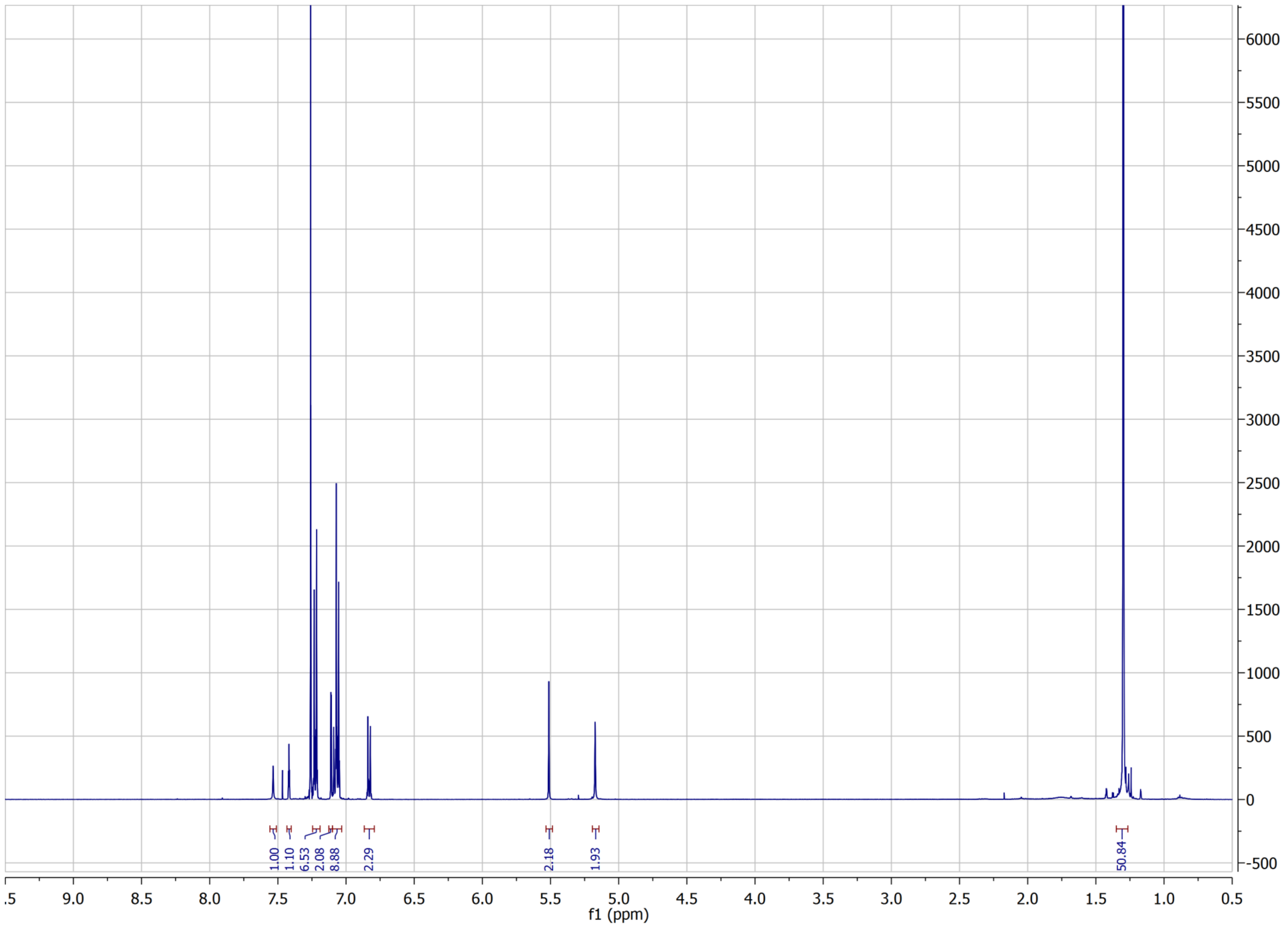

Thread S7: ^{13}C NMR ($CDCl_3$, 125 MHz, 300 K)

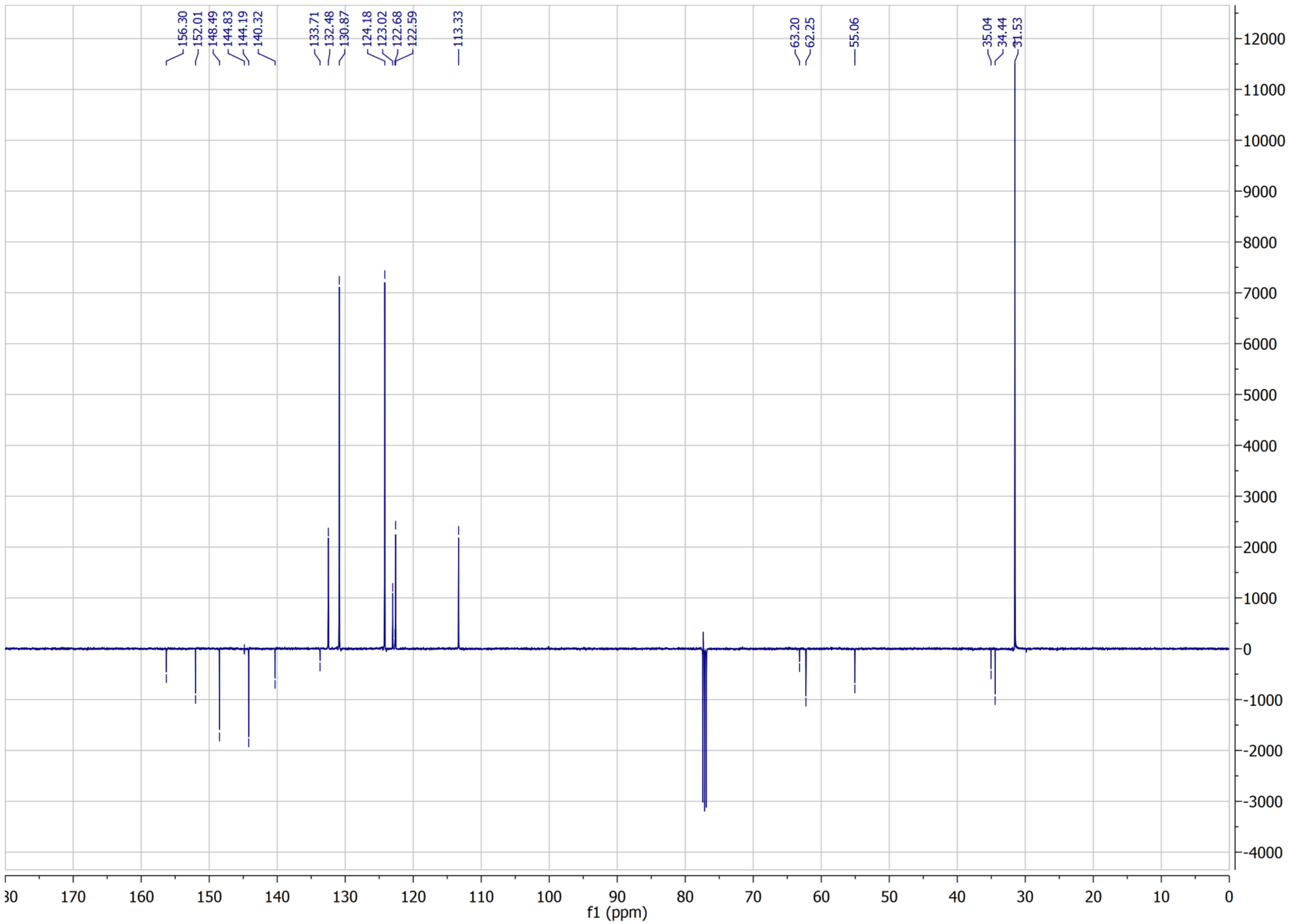

Thread S8: ^{1}H NMR ($CDCl_3$, 500 MHz, 300 K)

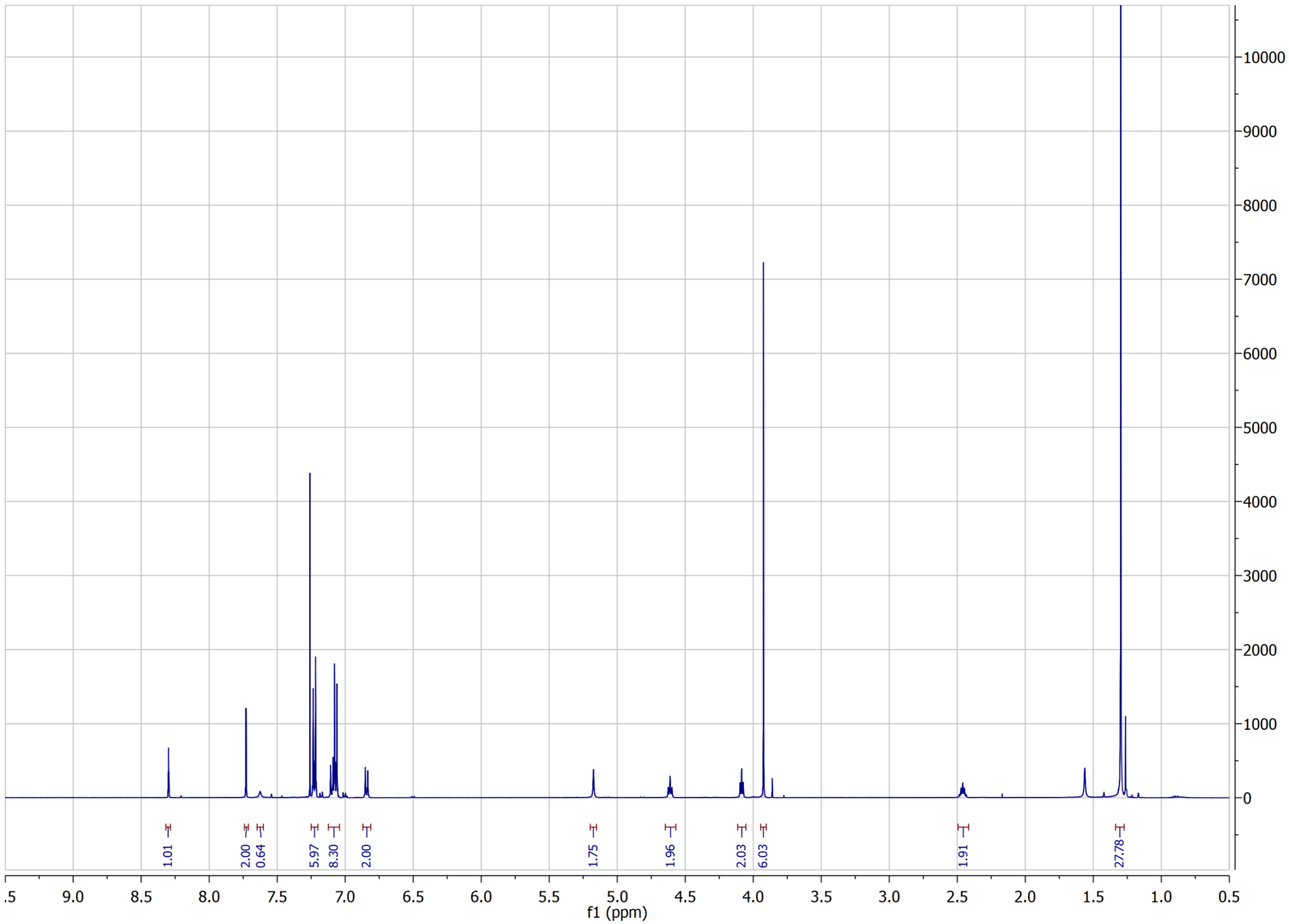

Thread S8: ^{13}C NMR ($CDCl_3$, 125 MHz, 300 K)

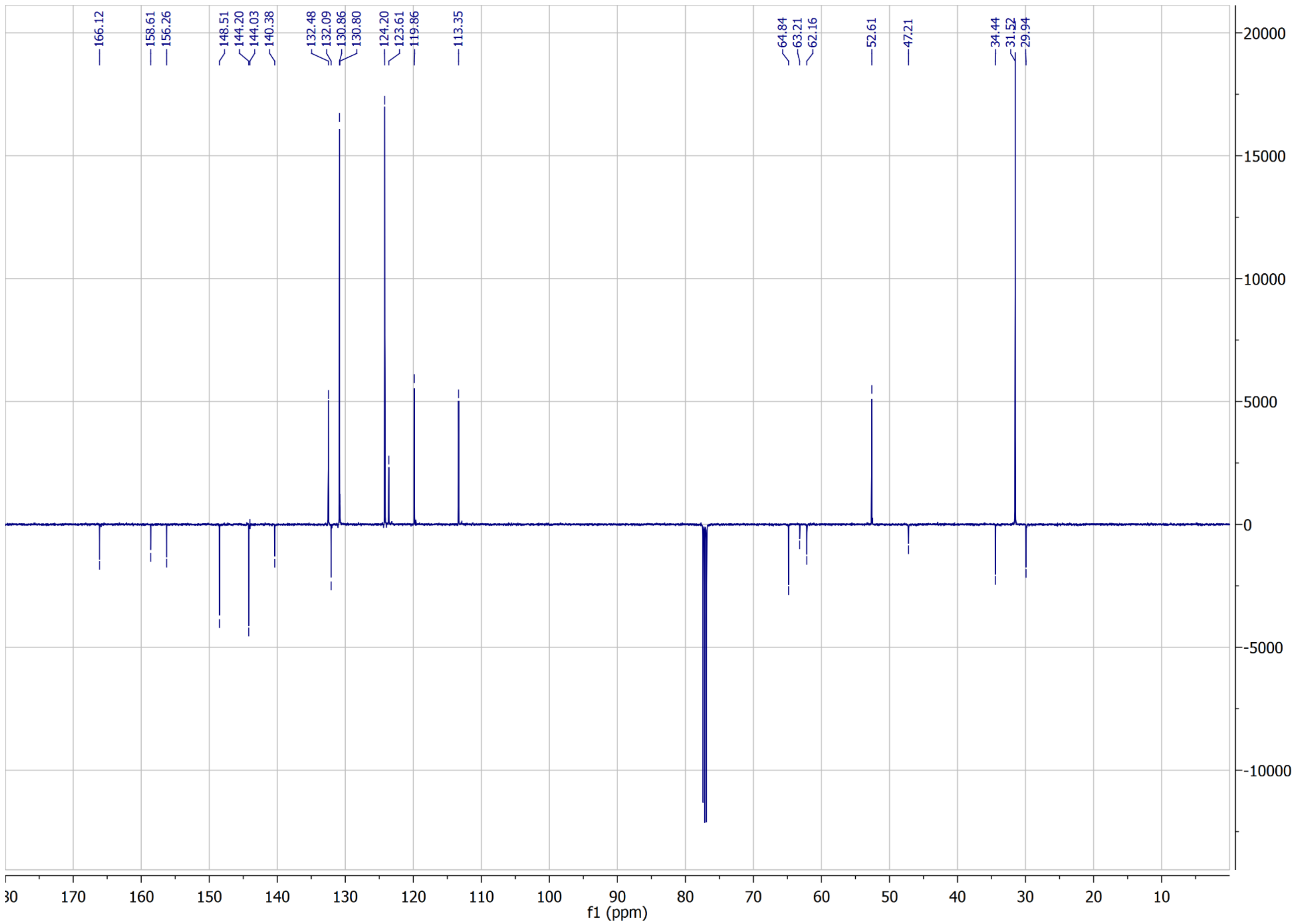

[3]Rotaxane 15c: ^{1}H NMR ($CDCl_3$, 500 MHz, 300 K)

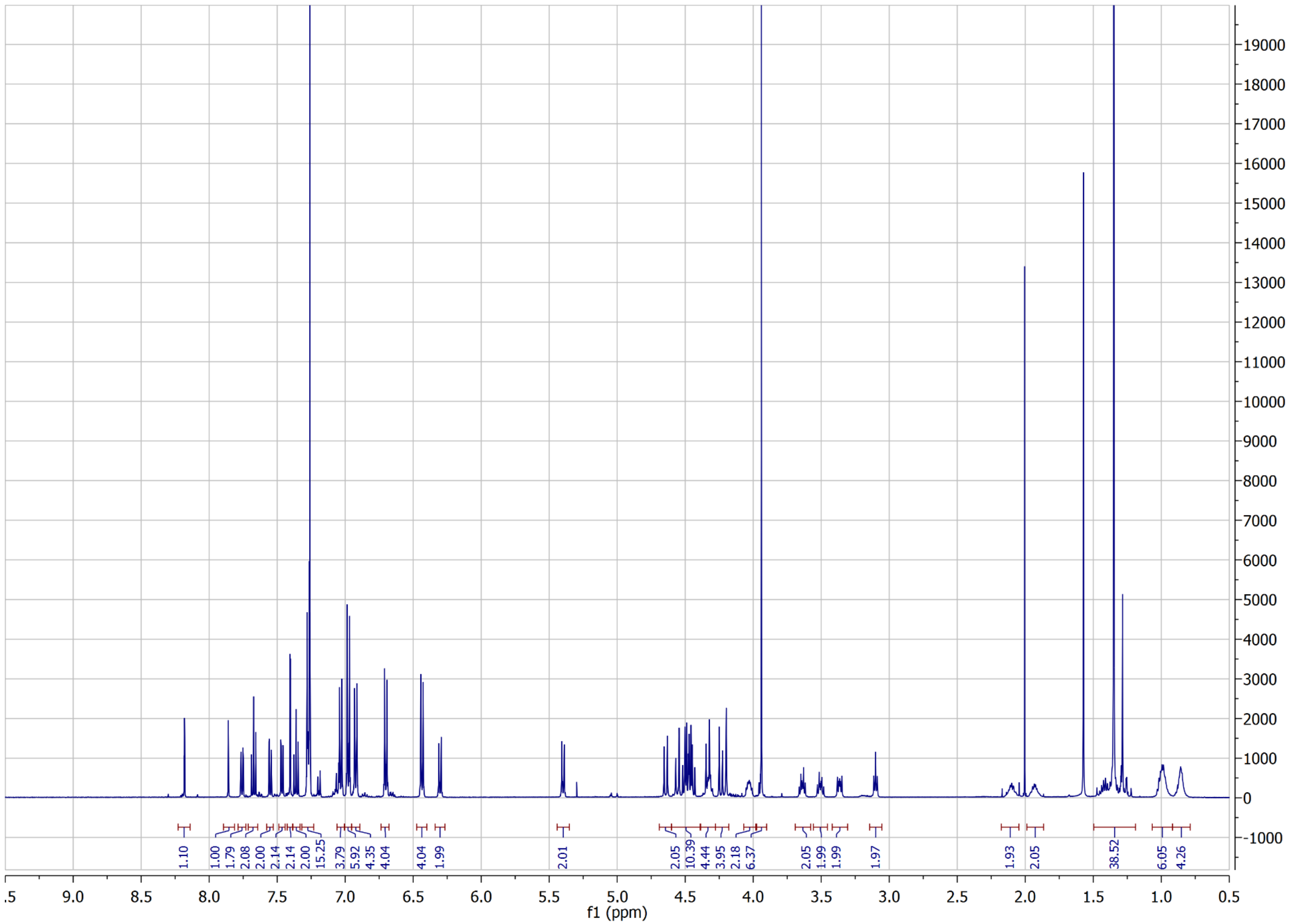

[3]Rotaxane 15c: ^{13}C NMR ($CDCl_3$, 125 MHz, 300 K)

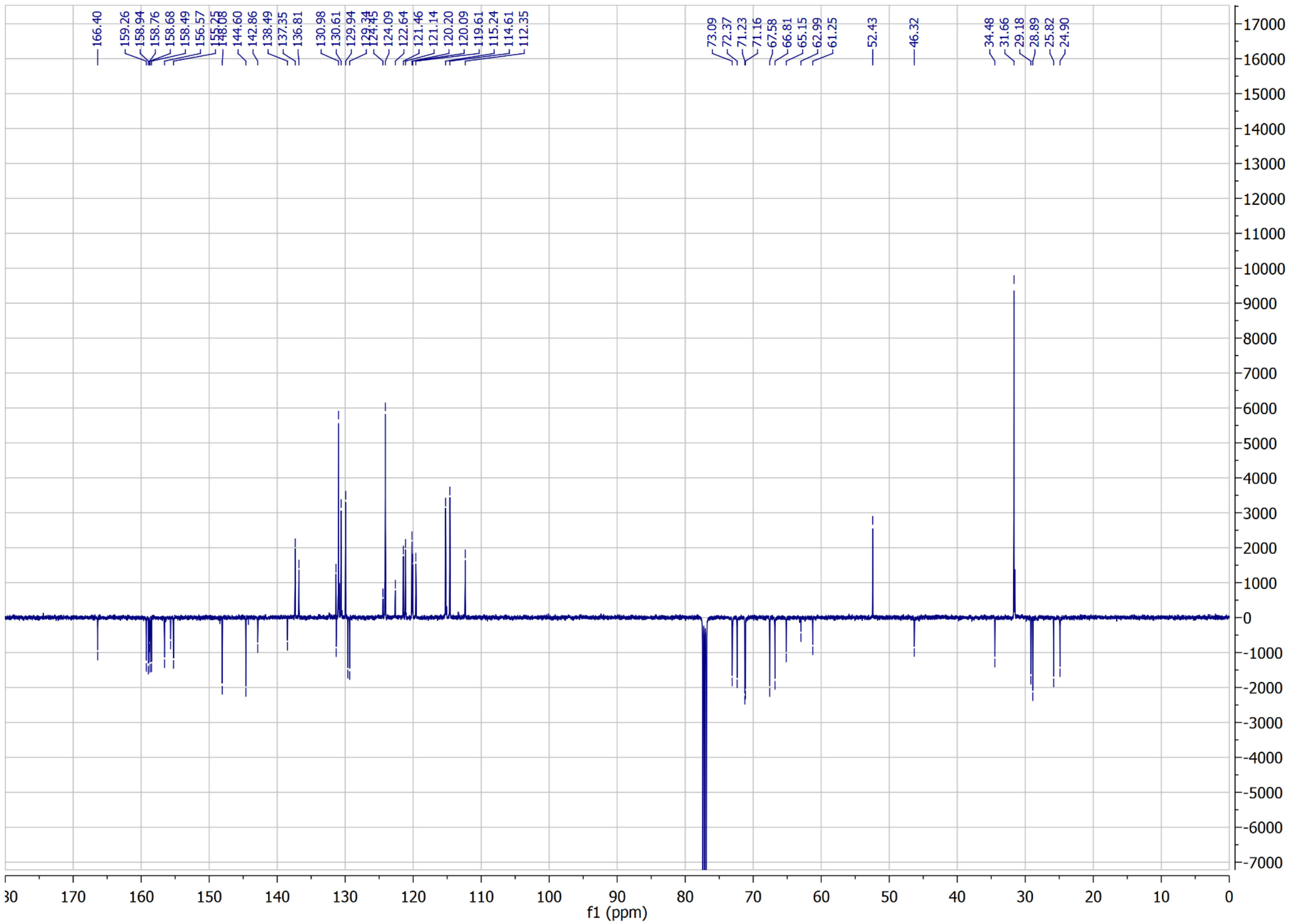

[3]Rotaxane 15c: COSY NMR ($CDCl_3$, 500 MHz, 300 K)

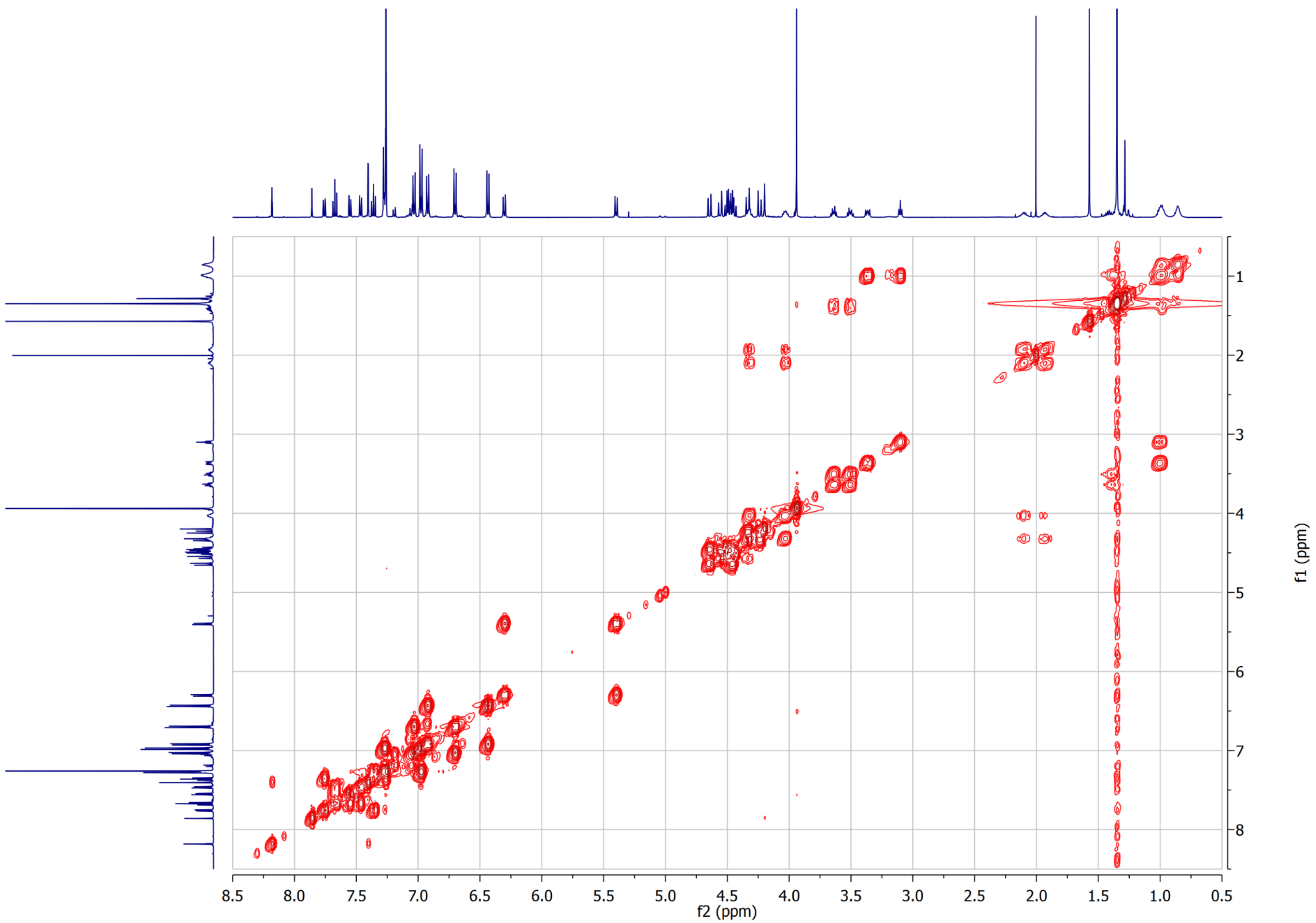

[3]Rotaxane 15c: HSQC NMR ($CDCl_3$, 500 MHz, 300 K)

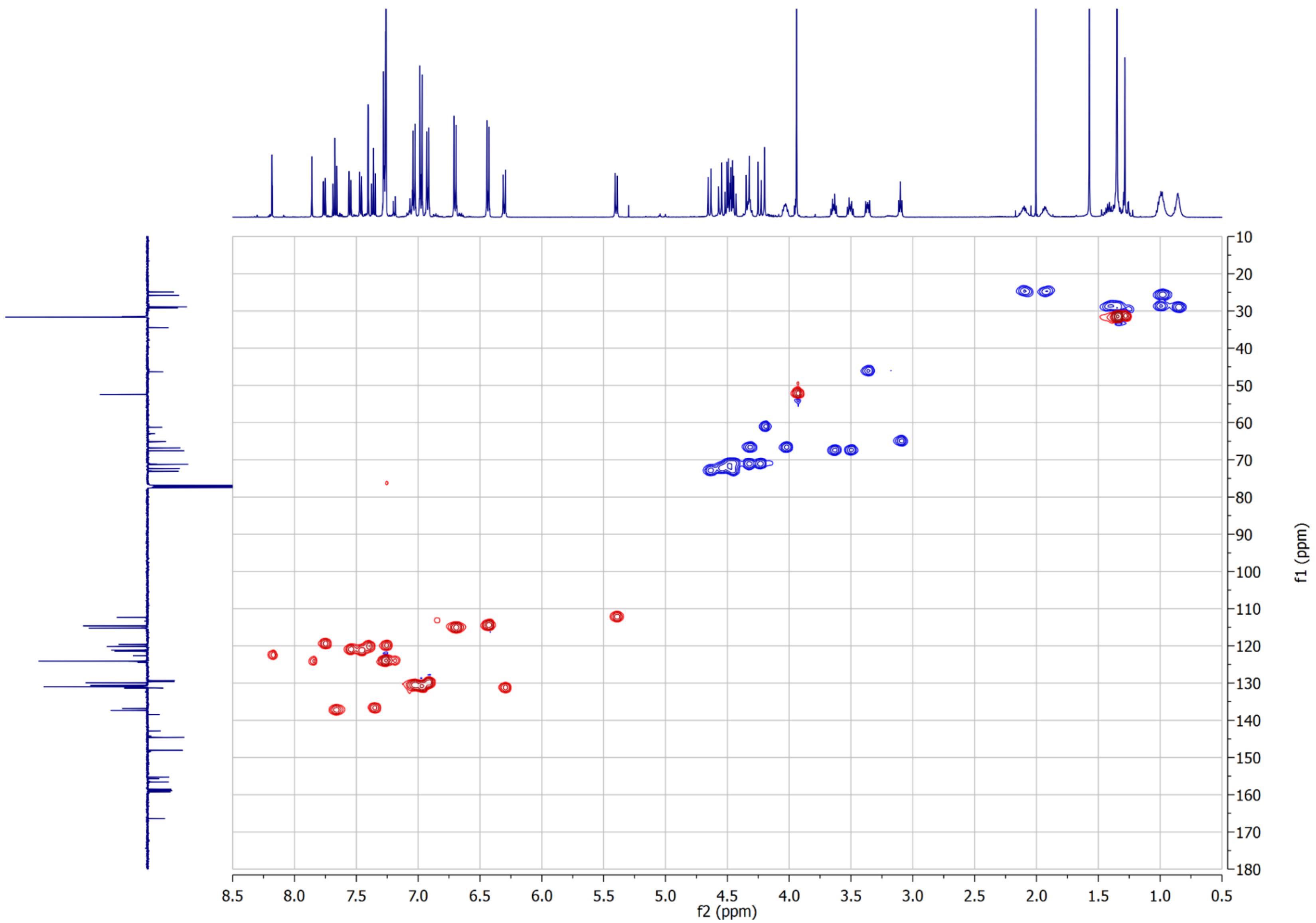

[3]Rotaxane 15c: HMBC NMR ($CDCl_3$, 500 MHz, 300 K)

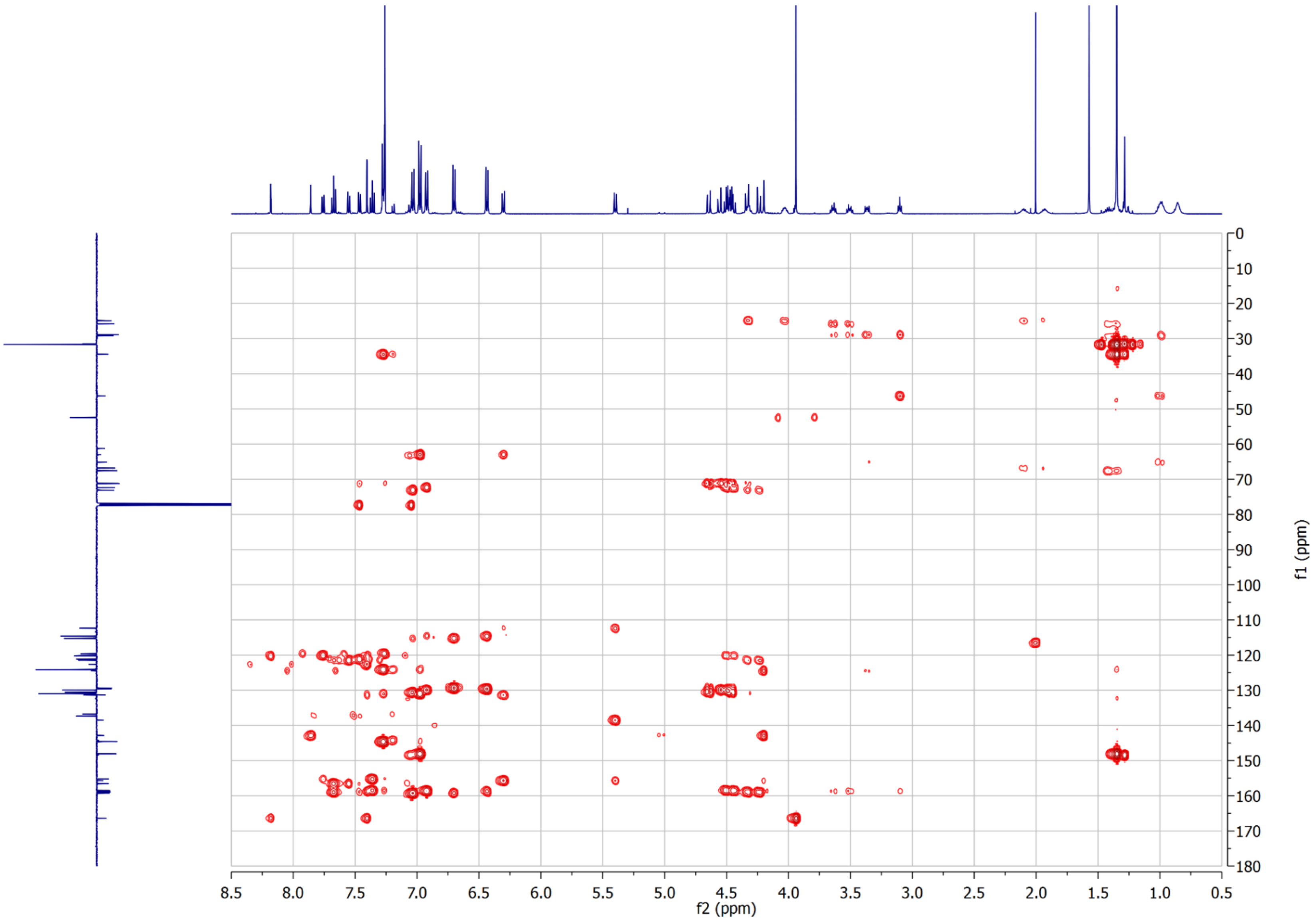

[3]Rotaxane 15c: ROESY NMR ($CDCl_3$, 500 MHz, 300 K)

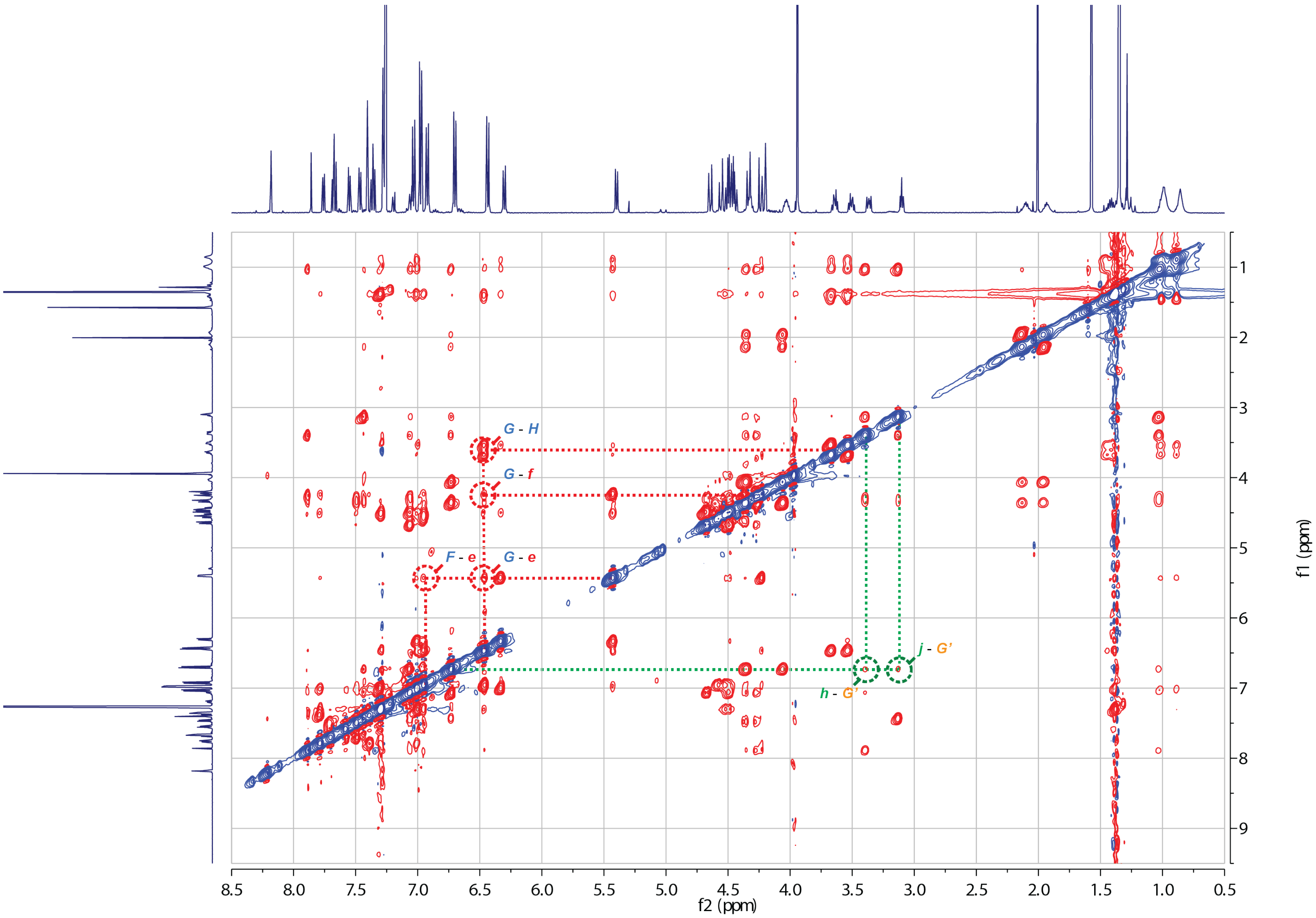

Alkyne S10: ^{1}H NMR (500 MHz, $CDCl_3$)

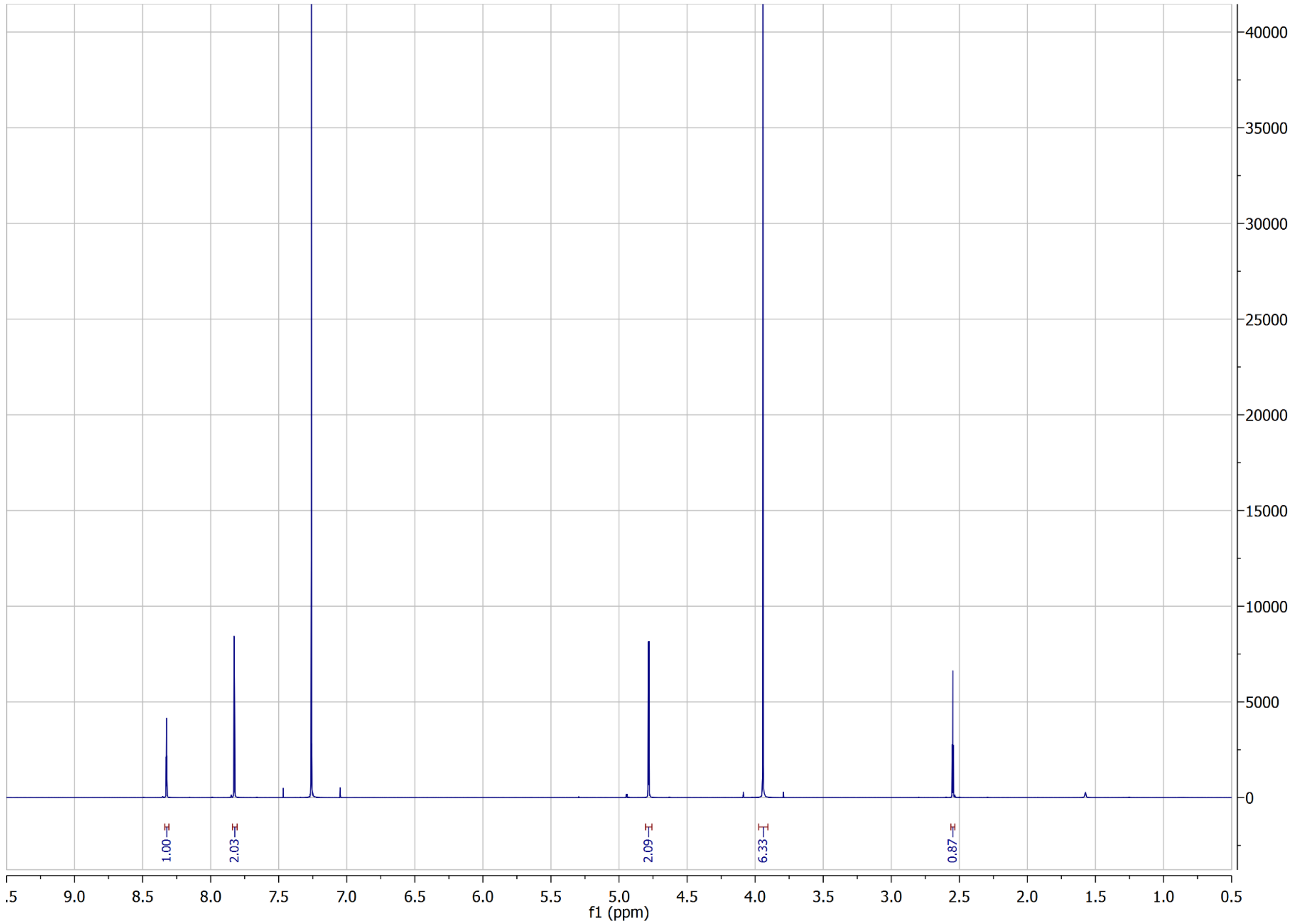

Alkyne S10: ^{13}C NMR (125 MHz, $CDCl_3$)

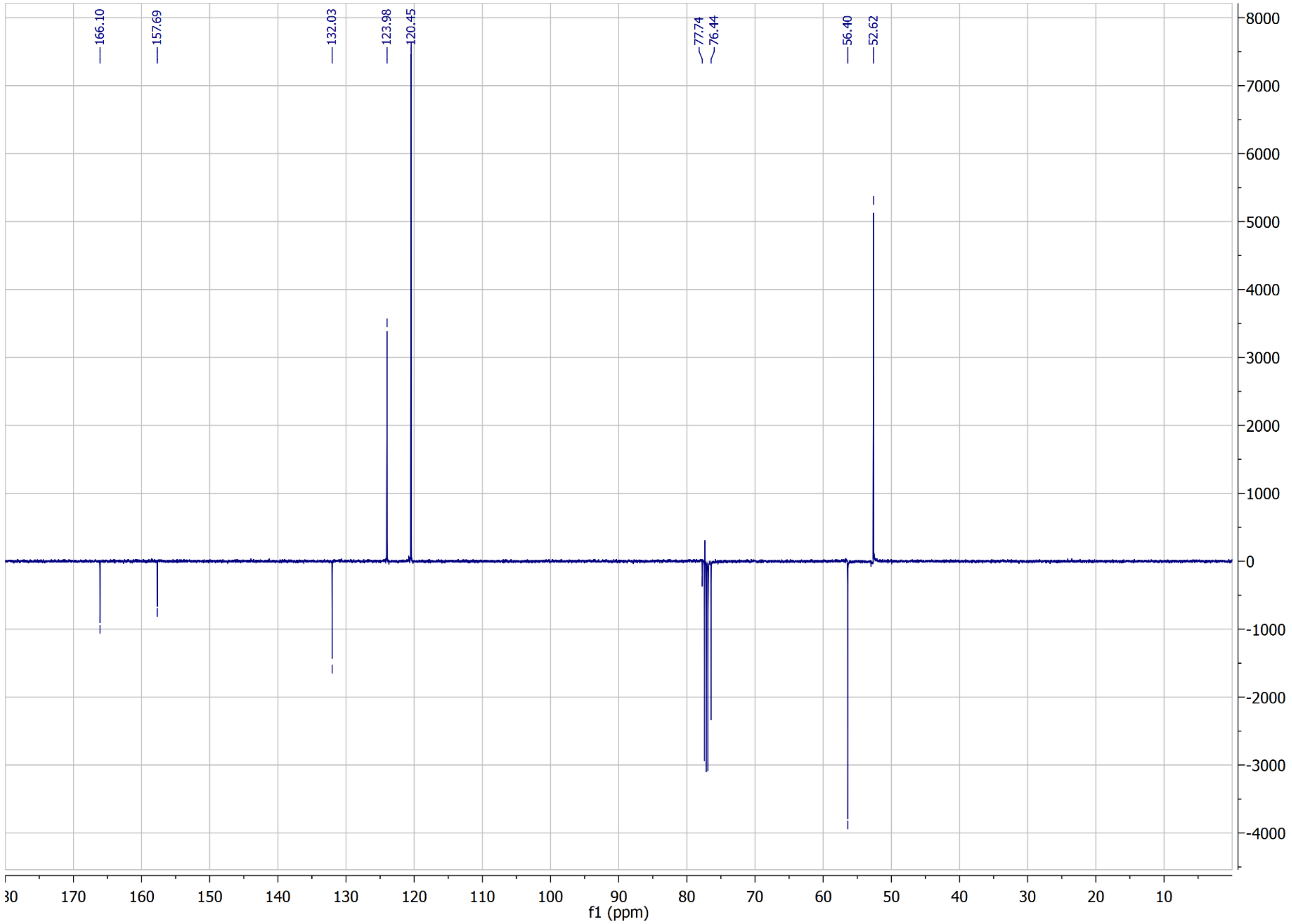

[2]Rotaxane S11: ^{1}H NMR (500 MHz, $CDCl_3$)

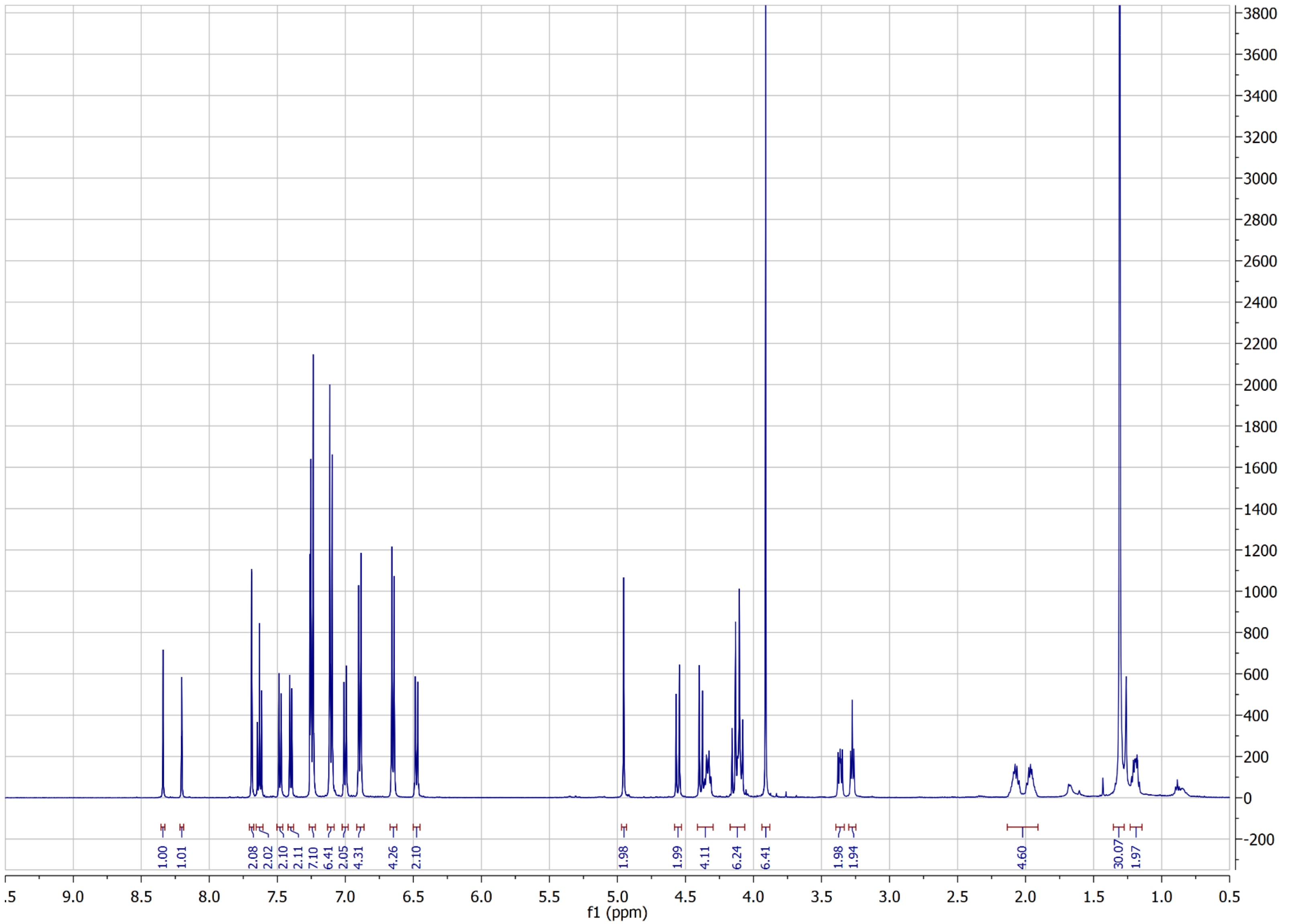

[2]Rotaxane S11: ^{13}C NMR (125 MHz, $CDCl_3$))

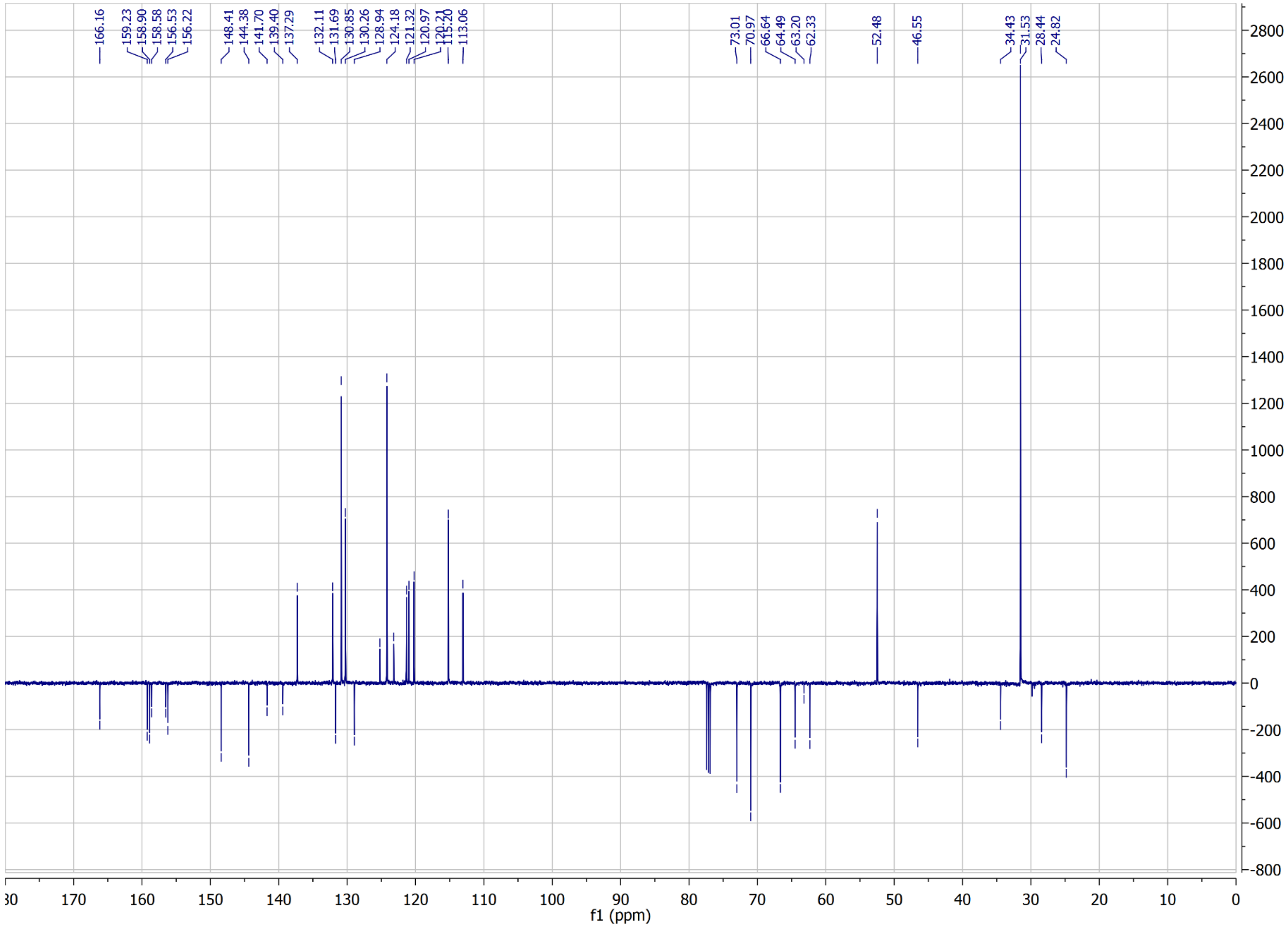

[2]Rotaxane S11: COSY NMR (500 MHz, $CDCl_3$)

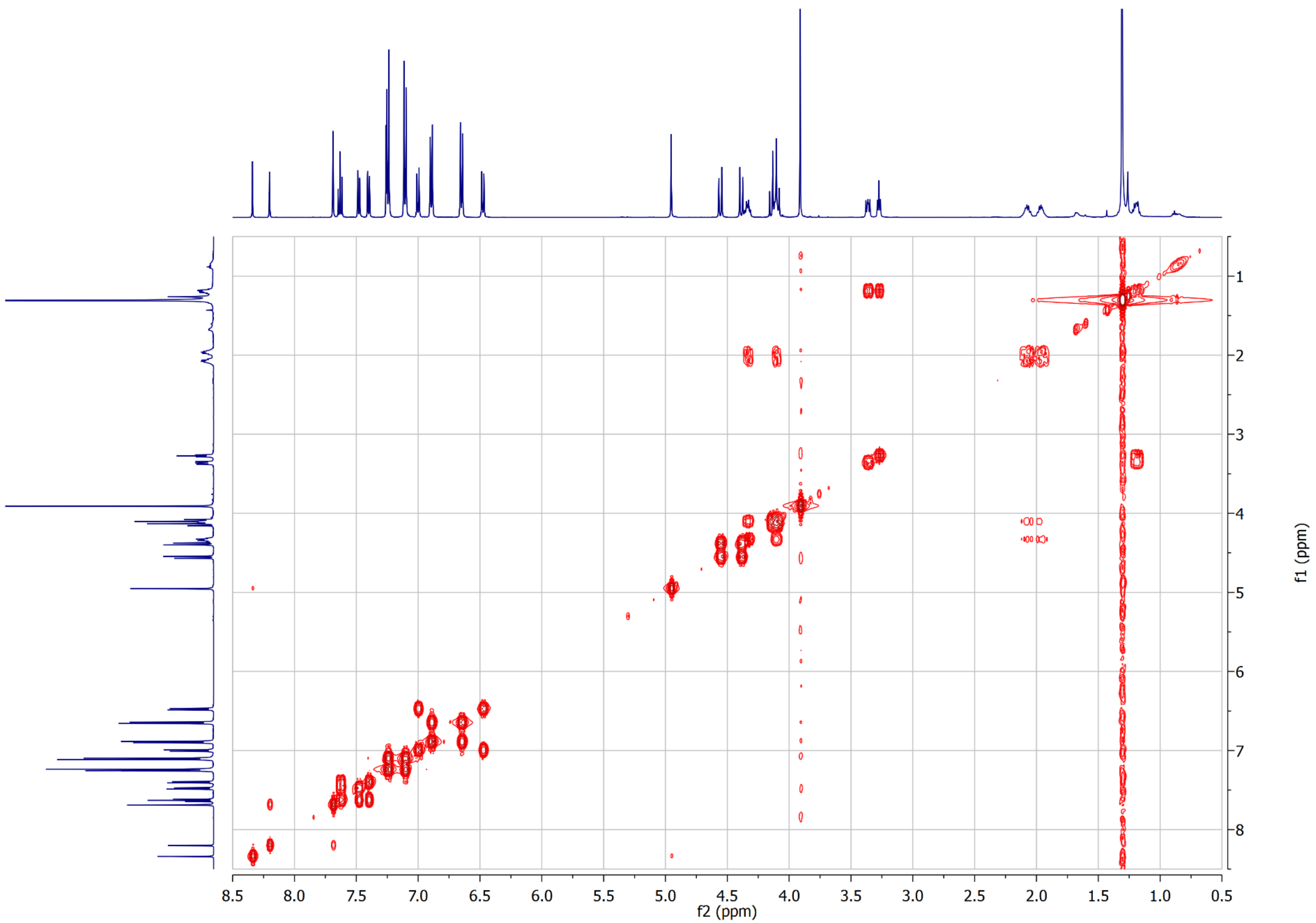

[2]Rotaxane S11: HSQC NMR (500 MHz, $CDCl_3$)

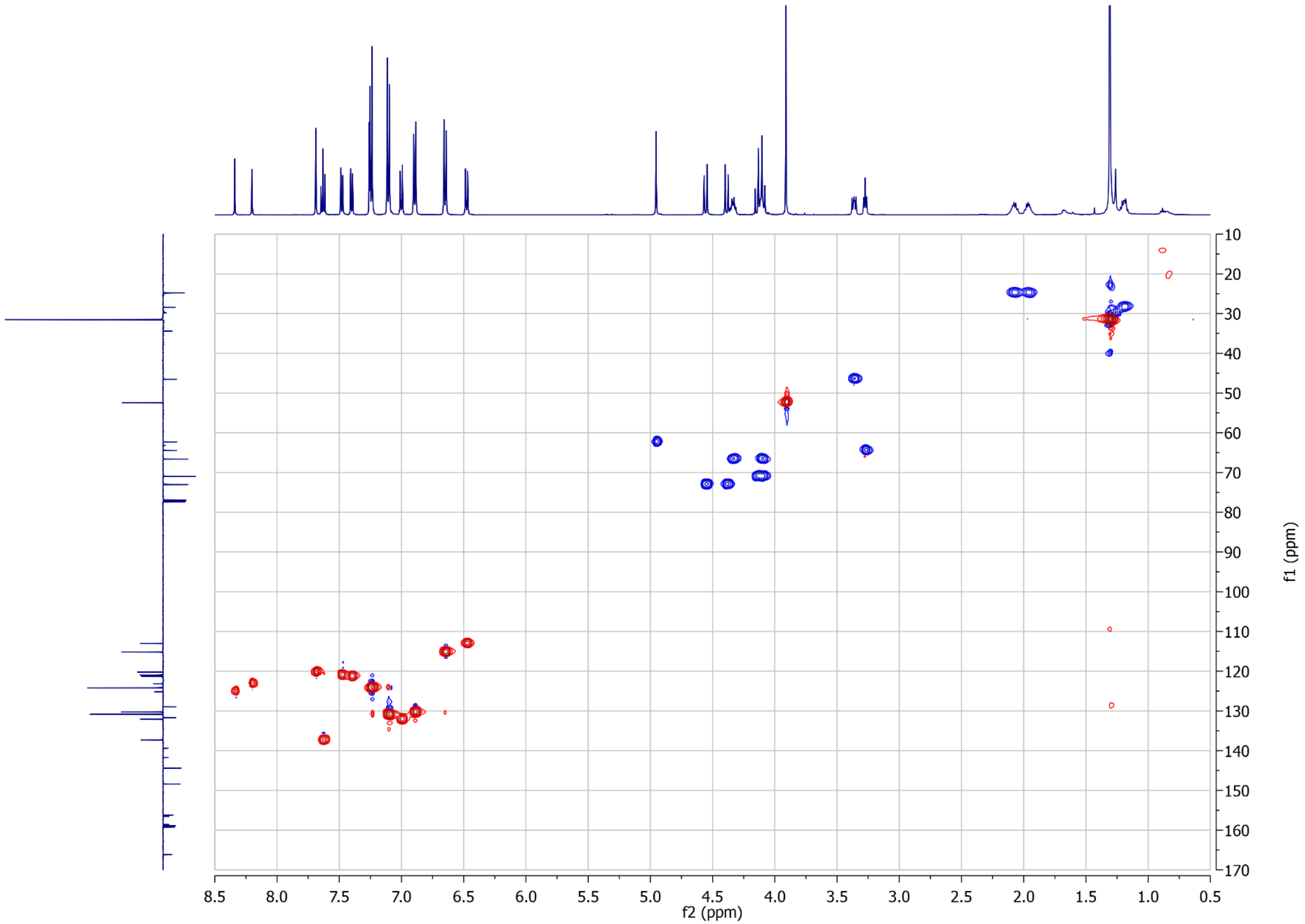

[2]Rotaxane S11: HMBC NMR (500 MHz, $CDCl_3$)

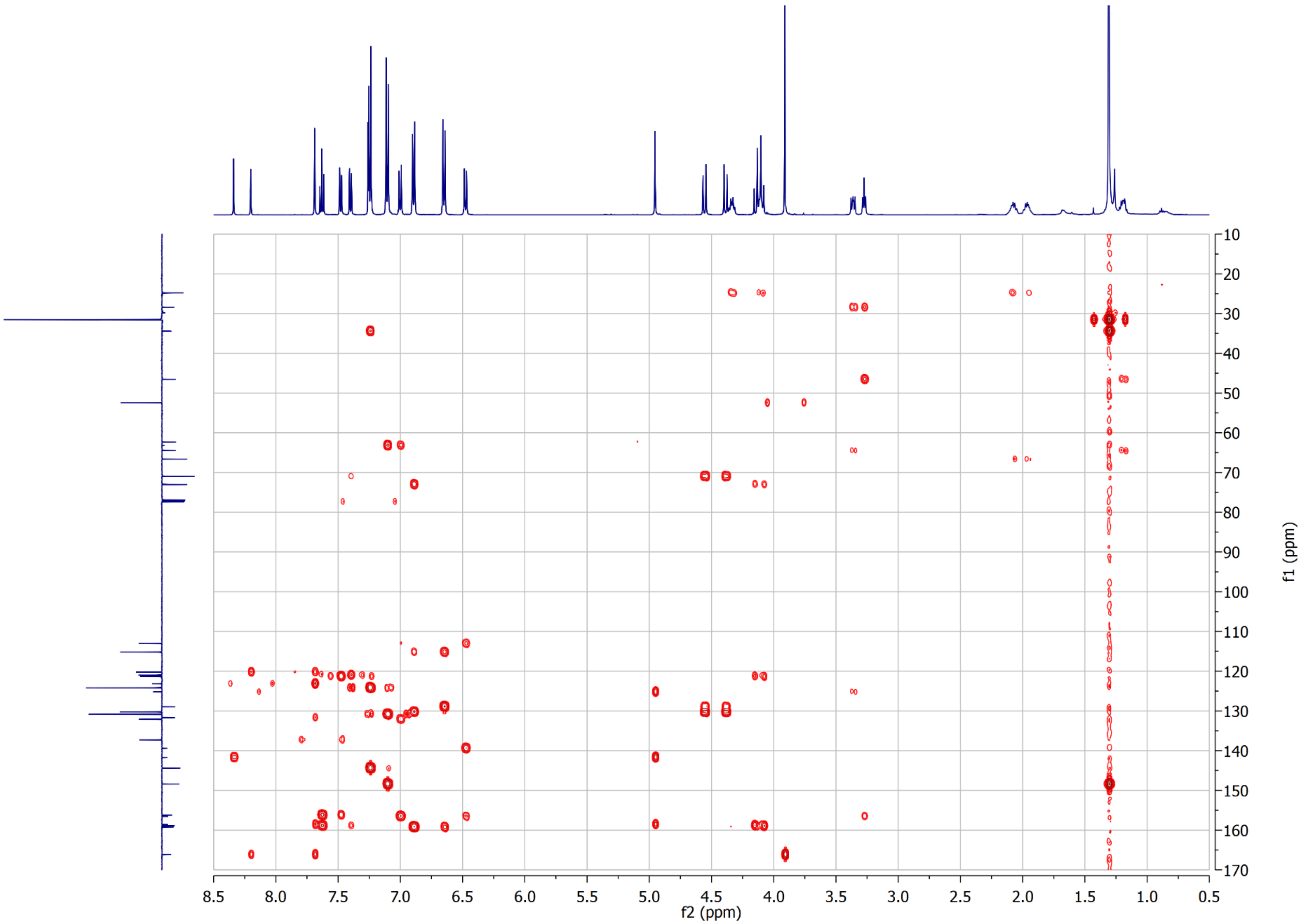

Thread S13: ^{1}H NMR (400 MHz, $CDCl_3$)

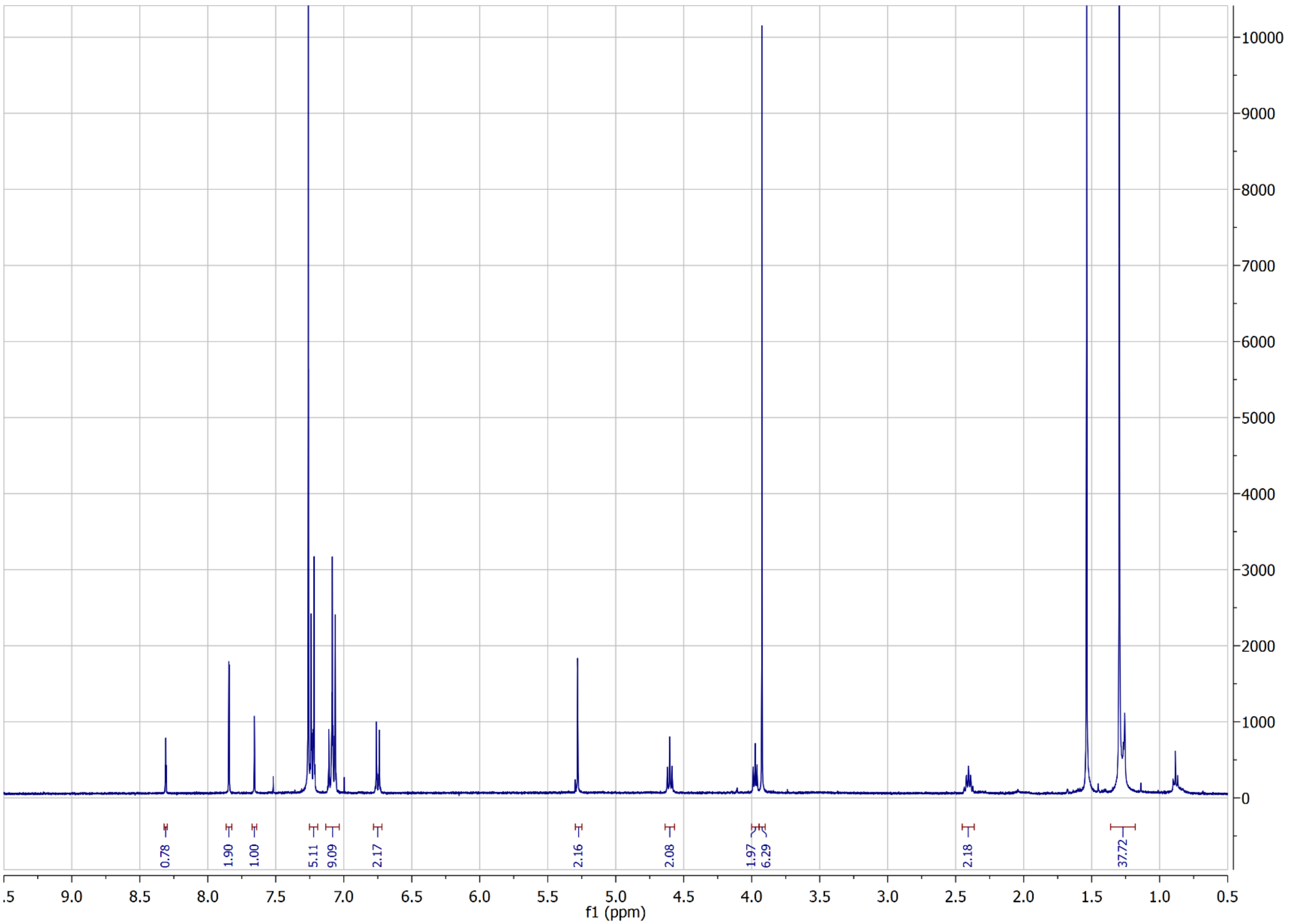

Thread S13: ^{13}C NMR (100 MHz, $CDCl_3$)

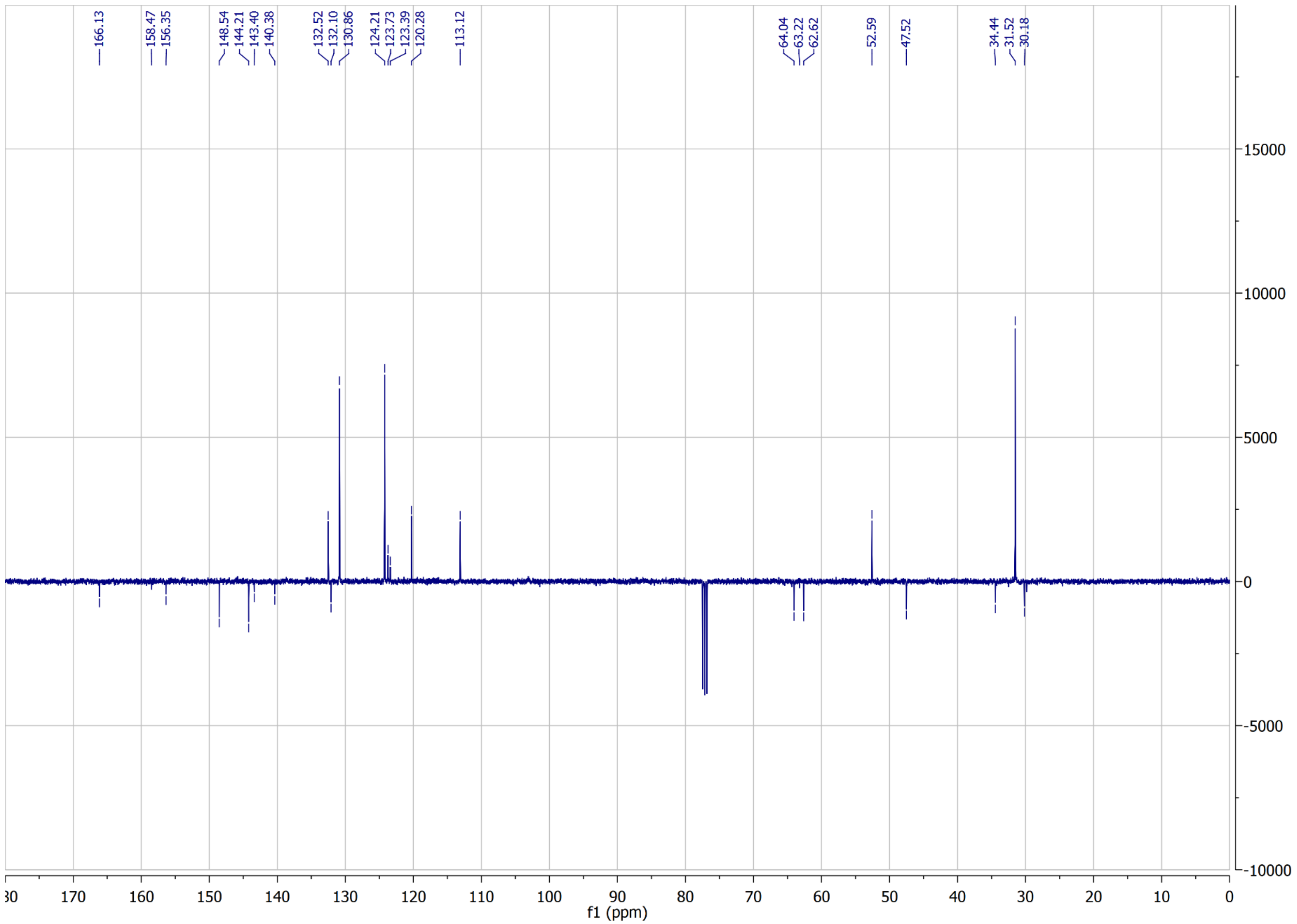

7. References

1. H. Lahlali, K. Jobe, M. Watkinson and S. M. Goldup, *Angew. Chem. Int. Ed.*, 2011, **50**, 4151-4155.
2. E. A. Neal and S. M. Goldup, *Chem. Sci.*
3. J. Winn, A. Pinczewska and S. M. Goldup, *J. Am. Chem. Soc.*, 2013, **135**, 13318-13321.
4. V. Aucagne, K. D. Hänni, D. A. Leigh, P. J. Lusby and D. B. Walker, *J. Am. Chem. Soc.*, 2006, **128**, 2186-2187.
5. W. Zhu and D. Ma, *Chem. Commun.*, 2004, 888-889.
6. J. J. Gassensmith, L. Barr, J. M. Baumes, A. Paek, A. Nguyen and B. D. Smith, *Org. Lett.*, 2008, **10**, 3343-3346.
7. V. Aucagne, J. Berná, J. D. Crowley, S. M. Goldup, K. D. Hänni, D. A. Leigh, P. J. Lusby, V. E. Ronaldson, A. M. Z. Slawin, A. Viterisi and D. B. Walker, *J. Am. Chem. Soc.*, 2007, **129**, 11950-11963.
